# Understanding protein evolutionary rate by integrating gene co-expression with protein interactions

**DOI:** 10.1186/1752-0509-4-179

**Published:** 2010-12-30

**Authors:** Kaifang Pang, Chao Cheng, Zhenyu Xuan, Huanye Sheng, Xiaotu Ma

**Affiliations:** 1 Department of Computer Science and Engineering, Shanghai Jiao Tong University, Shanghai, 200240, China; 2 Program in Computational Biology and Bioinformatics, Yale University, New Haven, CT 06520, USA; 3 Department of Molecular and Cell Biology, Center for Systems Biology, University of Texas at Dallas, Richardson, TX 75080, USA

## Abstract

**Background:**

Among the many factors determining protein evolutionary rate, protein-protein interaction degree (PPID) has been intensively investigated in recent years, but its precise effect on protein evolutionary rate is still heavily debated.

**Results:**

We first confirmed that the correlation between protein evolutionary rate and PPID varies considerably across different protein interaction datasets. Specifically, because of the maximal inconsistency between yeast two-hybrid and other datasets, we reasoned that the difference in experimental methods contributes to our inability to clearly define how PPID affects protein evolutionary rate. To address this, we integrated protein interaction and gene co-expression data to derive a co-expressed protein-protein interaction degree (ePPID) measure, which reflects the number of partners with which a protein can permanently interact. Thus, irrespective of the experimental method employed, we found that (1) ePPID is a better predictor of protein evolutionary rate than PPID, (2) ePPID is a more robust predictor of protein evolutionary rate than PPID, and (3) the contribution of ePPID to protein evolutionary rate is statistically independent of expression level. Analysis of hub proteins in the Structural Interaction Network further supported ePPID as a better predictor of protein evolutionary rate than the number of distinct binding interfaces and clarified the slower evolution of co-expressed multi-interface hub proteins over that of other hub proteins.

**Conclusions:**

Our study firmly established ePPID as a robust predictor of protein evolutionary rate, irrespective of experimental method, and underscored the importance of permanent interactions in shaping the evolutionary outcome.

## Background

Among the many factors determining protein evolutionary rate [1-5], protein-protein interaction degree (PPID), defined as the number of interaction partners a protein has in a protein interaction network, is an important predictor. A negative correlation between protein evolutionary rate and PPID was first reported in [6], which is consistent with the "functional density" hypothesis [7] that protein evolutionary rate is primarily determined by the proportion of residues involved in specific functions. Since then, several differing conclusions have been drawn. The controversies mainly focus on whether the correlation between PPID and protein evolutionary rate (1) is an artefact of biased protein interaction datasets [8-12], (2) is linked to experimental setup that favors counting more interactions for abundant proteins [13-15], or (3) is confounded by other genomic variables [16,17].

The relationship between protein evolutionary rate and PPID is mostly studied through hub proteins, i.e., proteins with a large number of interaction partners, from many different aspects [18-23]. For example, hub proteins can be classified into date and party hubs [24], singlish-interface and multi-interface hubs [22], singlish-iMotif and multi-iMotif hubs [23]. It was found that multi-interface hubs are mostly party hubs and singlish-interface hubs are mostly date hubs [22]. It was also found that party hubs evolve more slowly than date hubs [18,20] and multi-interface hubs evolve more slowly than singlish-interface hubs [22], but these findings are also challenged [19,21]. Furthermore, it was found that multi-iMotif hubs do not evolve more slowly than singlish-iMotif hubs [23]. These lines of evidence suggest a profound lack of consensus about the evolutionary rate differences between different types of hub proteins.

Therefore, in this paper, we first re-investigated the relationship between protein evolutionary rate and protein-protein interaction degree (PPID) and confirmed that the correlation between protein evolutionary rate and PPID varies considerably across different protein interaction datasets. We then integrated protein interaction and gene co-expression data to derive a co-expressed protein-protein interaction degree (ePPID) measure, which reflects the number of partners with which a protein can permanently interact. Our results demonstrated that ePPID is a more robust predictor of protein evolutionary rate than PPID. It was further found that the contribution of ePPID to protein evolutionary rate is statistically independent of expression level. Finally, we established that ePPID could predict protein evolutionary rate better than the number of distinct binding interfaces for hub proteins in the Structural Interaction Network and clarified the slower evolution of co-expressed multi-interface hub proteins over that of other hub proteins.

## Results

### Controversial correlations between PPID and protein evolutionary rate

**Figure 1:**
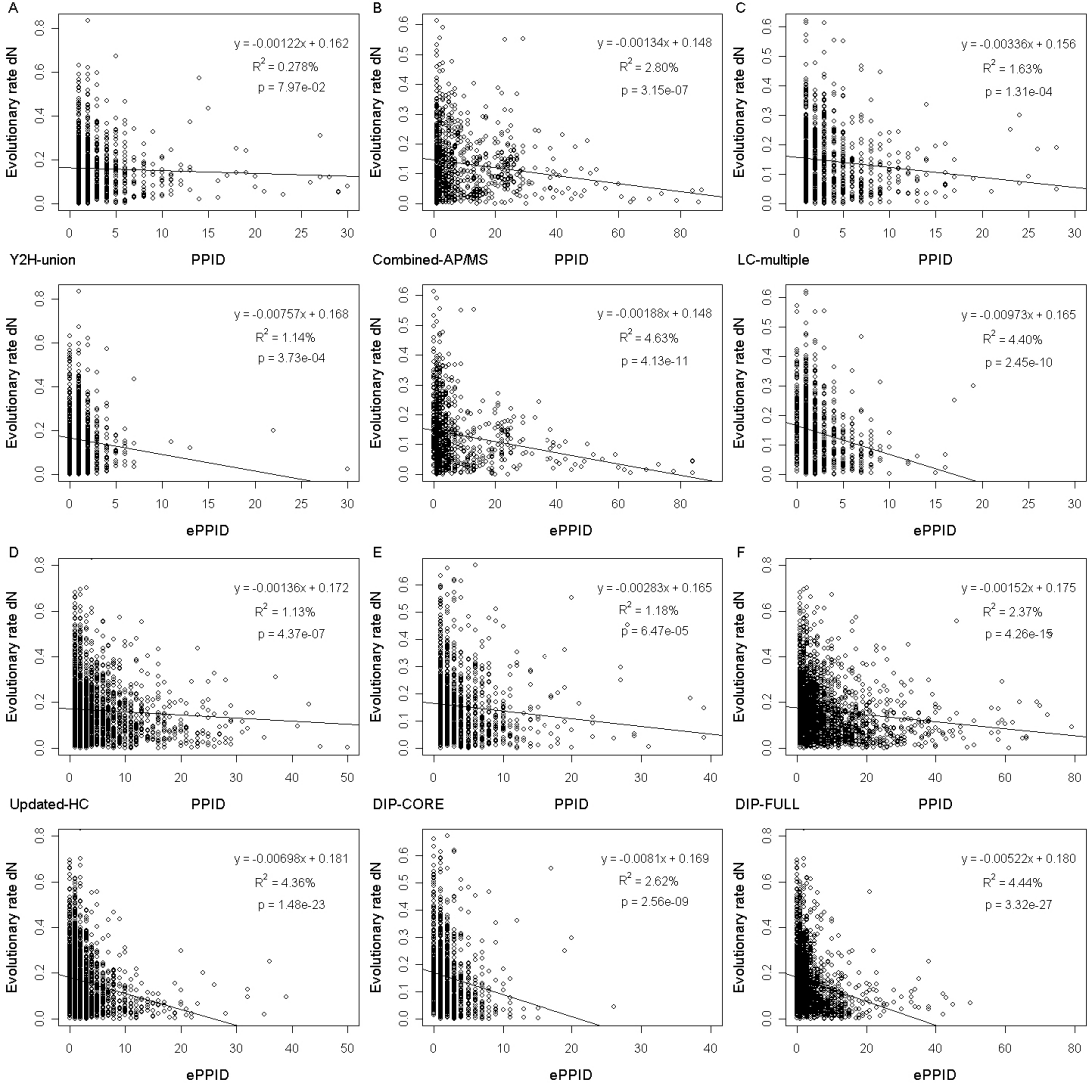
**Relationship of protein evolutionary rate dN with PPID and ePPID**. Scatter plots of protein evolutionary rate dN versus PPID (upper panel) and ePPID (bottom panel) together with the linear regression fit in the six protein interaction datasets: (A) "Y2H-union" (B) "Combined-AP/MS" (C) "LC-multiple" (D) "Updated-HC" (E) "DIP-CORE" (F) "DIP-FULL".

Researchers have found very different correlations between PPID and protein evolutionary rate [6,8-17]. To address this variation, we first obtained the non-synonymous substitution rate (dN) data on yeast [25] for protein evolutionary rate (see Methods). Next, to account for experimental bias, reliability and completeness [26-32], nine yeast protein interaction datasets were compiled from different sources (see Methods). We analyzed six protein interaction datasets in the main text and the analysis results of the other three were provided in Additional file 1, Text S1. Scatter plots of protein evolutionary rate dN versus PPID, together with linear regression fit, are shown in the upper panels of Figure 1A-C for the "Y2H-union", "Combined-AP/MS" and "LC-multiple" datasets, and in the upper panels of Figure 1D-F for the "Updated-HC", "DIP-CORE" and "DIP-FULL" datasets. For the six protein interaction datasets, negative correlation coefficients between PPID and protein evolutionary rate are observed. However, the statistical significance of these correlation coefficients varies considerably across different protein interaction datasets, which is consistent with previous results [6,8-17]. Specifically, significant results are observed for protein interaction datasets that include "Combined-AP/MS", "LC-multiple", "Updated-HC", "DIP-CORE" and "DIP-FULL" (Figure 1B-F). The "Combined-AP/MS" and "LC-multiple" datasets were compiled from the affinity purifications followed by the mass spectrometry (AP/MS) method and literature curation, respectively, while the other three datasets were compiled from diverse data sources. On the other hand, an insignificant result is observed for the "Y2H-union" dataset (Figure 1A), which is only compiled from yeast two-hybrid (Y2H) assays. Moreover, the percent variance of evolutionary rate explained by PPID is also the lowest in the "Y2H-union" dataset (Figure 1 and column 3 of Additional file 1, Table S1). To account for the non-normality of the distribution of PPID, we also computed Spearman rank correlation between PPID and protein evolutionary rate and found that the correlations are highly significant in all the six datasets except "Y2H-union" (column 3 of Table 1). These results suggest that the differing results reached by previous investigators may be related to the difference between Y2 H and other experimental methods, possibly because Y2 H datasets do not have abundance bias and/or are enriched for transient protein interactions [26,32].

**Table 1:** Spearman correlation of PPID, ePPID and betweenness with protein evolutionary rate.

Protein interaction datasets	*n*	PPID vs. dN	ePPID vs. dN	betweenness vs. dN
	
		*rho*(*p*)	*rho*(*p*)	*rho*(*p*)
Y2H-union	1,104	-0.0487(1.06e-01)	**-0.142(2.25e-06)**	-0.0365(2.26e-01)

Combined-AP/MS	922	**-0.158(1.46e-06)**	**-0.251(1.03e-14)**	**-0.241(1.21e-13)**

LC-multiple	894	**-0.172(2.46e-07)**	**-0.267(4.72e-16)**	**-0.186(2.05e-08)**

Updated-HC	2,245	**-0.183(2.62e-18)**	**-0.242(2.58e-31)**	**-0.128(1.30e-09)**

DIP-CORE	1,342	**-0.152(2.33e-08)**	**-0.254(3.69e-21)**	**-0.111(4.53e-05)**

DIP-FULL	2,572	**-0.233(4.56e-33)**	**-0.271(1.40e-44)**	**-0.188(5.90e-22)**

dN represents protein evolutionary rate measured by non-synonymous substitutions. *n *is the number of proteins for which both PPID and protein evolutionary rate are available. *rho *is Spearman rank correlation coefficient, and *p *is the corresponding statistical significance. Bold indicates that *p *is significant at the statistical significance level of 0.05.

We next studied if the protein abundance effect may account for the above significant difference between the Y2 H and other datasets. We computed Spearman rank correlations between PPID and protein abundance [33] and found that the PPID measure may contain independent information for protein evolutionary rate (see Additional file 1, Text S2). Then, we studied the percentage variance of protein evolutionary rate explained by PPID when protein abundance is controlled for (column 4 of Table 2). As can be seen, considerable percent variances of evolutionary rate explained by PPID remain in all the six protein interaction datasets. In addition, the partial Spearman correlation coefficients between PPID and protein evolutionary rate are still significant (though marginally significant in the "Combined-AP/MS" dataset) after controlling for protein abundance (column 4 of Table 3), with the exception of "Y2H-union" datasets, suggesting that PPID contains independent information for evolutionary rate (similar results were obtained when using other expression-related data [34-36], data not shown). Thus, we next wanted to study how to better understand evolutionary rate by integrating PPID with gene expression data.

**Table 2:** The variance of protein evolutionary rate explained by PPID and ePPID when controlling for protein abundance.

Protein interaction datasets	*n*	Percent variance explained in dN			
		
		PPID(*p*)	PPID control for Log(abundance)(*p*)	ePPID(*p*)	ePPID control for Log(abundance)(*p*)
Y2H-union	793	0.110(3.52e-01)	0.145(2.84e-01)	**0.659(2.22e-02)**	**0.547(3.73e-02)**

Combined-AP/MS	763	**2.50(1.13e-05)**	**0.906(8.52e-03)**	**4.25(9.08e-09)**	**0.999(5.73e-03)**

LC-multiple	680	**1.29(3.01e-03)**	**1.17(4.72e-03)**	**4.11(9.62e-08)**	**1.70(6.59e-04)**

Updated-HC	1,587	**0.722(7.04e-04)**	**0.547(3.19e-03)**	**3.53(4.39e-14)**	**1.32(4.60e-06)**

DIP-CORE	968	0.308(8.42e-02)	0.109(3.05e-01)	**1.27(4.44e-04)**	0.109(3.05e-01)

DIP-FULL	1,792	**1.99(2.03e-09)**	**0.996(2.32e-05)**	**3.99(1.39e-17)**	**1.36(7.21e-07)**

dN represents protein evolutionary rate measured by non-synonymous substitutions. *n *is the number of proteins for which PPID, protein evolutionary rate and abundance data are all available. *p *in column 3 and 5 is the statistical significance of the linear regression of protein evolutionary rate against PPID and ePPID, respectively. *p *in column 4 and 6 is the statistical significance of the linear regression of protein evolutionary rate against PPID and ePPID when controlling for protein abundance, respectively. Bold indicates that *p *is significant at the statistical significance level of 0.05.

**Table 3:** Spearman correlation and partial Spearman correlation of PPID and ePPID with protein evolutionary rate.

Protein interaction datasets	*n*	PPID vs. dN	PPID vs. dN control for abundance	ePPID vs. dN	ePPID vs. dN control for abundance
		
		*rho*(*p*)	*rho*(*p*)	*rho*(*p*)	*rho*(*p*)
Y2H-union	793	-0.0249(4.85e-01)	-0.0260(4.64e-01)	**-0.116(1.06e-03)**	**-0.0930(8.66e-03)**

Combined-AP/MS	763	**-0.135(1.85e-04)**	-0.0666(6.59e-02)	**-0.229(1.51e-10)**	**-0.103(4.35e-03)**

LC-multiple	680	**-0.149(9.75e-05)**	**-0.134(4.16e-04)**	**-0.260(6.16e-12)**	**-0.179(2.14e-06)**

Updated-HC	1,587	**-0.140(2.27e-08)**	**-0.0992(7.24e-05)**	**-0.208(5.21e-17)**	**-0.121(1.14e-06)**

DIP-CORE	968	**-0.108(7.72e-04)**	**-0.0679(3.45e-02)**	**-0.214(1.72e-11)**	**-0.110(5.65e-04)**

DIP-FULL	1,792	**-0.223(1.24e-21)**	**-0.162(3.31e-12)**	**-0.252(2.77e-27)**	**-0.156(2.69e-11)**

dN represents protein evolutionary rate measured by non-synonymous substitutions. *n *is the number of proteins for which PPID, protein evolutionary rate and abundance data are all available. *rho *is Spearman rank correlation coefficient, and *p *is the corresponding statistical significance. Bold indicates that *p *is significant at the statistical significance level of 0.05.

### Co-expressed protein-protein interaction degree (ePPID) predicts protein evolutionary rate better than PPID

Proteins with higher PPID are assumed to have a greater proportion of residues involved in interactions and thus evolve more slowly than proteins with lower PPID [6,9]. This may be true for a protein with many permanent interaction partners, because the protein tends to form a permanent complex with its partners through multiple distinct binding interfaces and may have a greater proportion of interface residues [22]. However, a protein with many transient interaction partners may transiently interact with its different partners through the same binding interface (though it is possible that the protein may form a transient complex with its partners through multiple distinct binding interfaces), thus the PPID of the protein may not well reflect the proportion of its interface residues [22]. Furthermore, interface residues of permanent interactions are found to evolve more slowly than those of transient interactions [37,38]. In other words, permanent interactions are more likely to exert higher selective constrains on protein evolution [18,20,22,37-39] and protein evolutionary rate may be more reflective of the proportion of residues involved in permanent interactions. On the other hand, permanent interactions tend to show significant co-expression [32,40], so we speculate that the number of a protein's co-expressed interaction partners may well reflect the proportion of its residues involved in permanent interactions and thus better predict its evolutionary rate.

**Figure 2:**
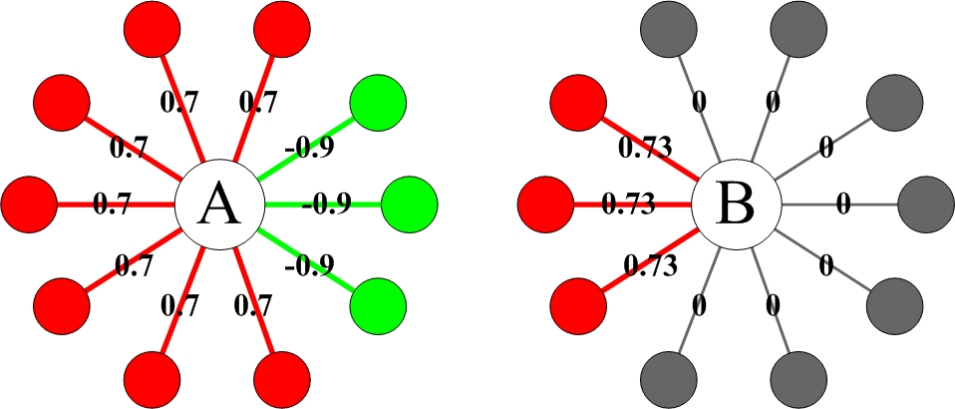
**Difference between ePPID and APCC measures**. Two hypothetical proteins with the same APCC score ((0.7 × 7 - 0.9 × 3) ÷ 10 = 0.22 for protein A and (0.73 × 3 + 0 × 7) ÷ 10 = 0.22 for protein B) but different numbers of significantly co-expressed interaction partners, thus differing ePPID (see the main text for details) scores (seven versus three). Significantly highly co-expressed interactions are indicated using red, while non-co-expressed and highly anti-co-expressed interactions are indicated using gray and green, respectively.

Several studies have addressed the difference in selective constraints between permanent and transient interactions on protein evolution [18,20,22,39]. For example, Han et al. [24] used the average Pearson correlation coefficient (APCC) between the expression profiles of a protein and its interaction partners to classify hub proteins into date (with lower APCC score) and party (with higher APCC score) hubs. Date hubs interact with their partners transiently, while party hubs interact with their partners permanently by co-expression. Thus, party hubs have a lower evolutionary rate than date hubs since selective constraints from permanent interactions on party hubs are higher than those from transient interactions on date hubs [18,20]. However, there are at least three drawbacks in using APCC scoring to account for transient protein interactions. First, while there was a bimodal distribution of the APCC scores in the "FYI" dataset [24], no clear bimodal distribution was found in the "DIP-CORE" dataset [41-43], a complete lack of bimodality was observed in several larger high-confidence datasets [19,21], and no robust bimodal distribution was found in the Online Predicted Human Interaction Database [44,45]. Thus, it is difficult to set the APCC threshold to distinguish party hubs from date hubs. Second, as an average measure, a high variance of Pearson correlation coefficient (PCC) scores between a hub and its interaction partners will make its APCC score less informative. For example, the APCC score of protein A in Figure 2 is 0.22, and a moderate APCC score cutoff would classify this protein as a date hub, which is clearly not our intention. Third, the APCC score only measures the average co-expression strength between a hub and its interaction partners, rather than the actual number of interaction partners with which the hub significantly co-expresses. For example, the APCC scores of protein A and B in Figure 2 are the same (0.22), but protein A and B have different numbers of significantly co-expressed interaction partners (seven versus three), which is again not our intention. In fact, several real proteins with low APCC score but high number of co-expressed interaction partners are exemplified in Additional file 1, Text S3. Another attempt was made by Kim et al. [22], who used the number of distinct binding interfaces of a hub to filter out transient protein interactions. However, since protein structure data are limited, it is impossible to project all structural information onto protein interaction datasets, and, as a result, the number of distinct binding interfaces of a hub may be underestimated. Furthermore, they did not clearly distinguish permanent interfaces from transient interfaces. For example, although a multi-interface hub is more likely to form a permanent complex with its partners through permanent interfaces, it does not rule out the possibility that the multi-interface hub forms a transient complex with its partners through transient interfaces. For another example, the number of distinct binding interfaces of a protein only implies the total number of partners with which it can potentially interact. In nature, however, it is possible that a multi-interface hub transiently interacts with its individual partner through the corresponding binding interface at different spatial-temporal conditions.

Therefore, we proposed a co-expressed protein-protein interaction degree (ePPID), defined as the maximal number of co-expressed interaction partners of a given protein in all gene expression datasets we used (in fact, other variations of such definition yield similar results, see Additional file 1, Text S4), to estimate the number of partners with which a protein can permanently interact (see Methods). It can be seen from the bottom panels of Figure 1A-C and Figure 1D-F that the ePPID measure has statistically significant negative correlation coefficients with protein evolutionary rate across all protein interaction datasets we studied. As shown in Table 1 (column 4 versus column 3), it is clear that the statistical significance obtained by ePPID is better than that obtained by PPID. Accordingly, across all protein interaction datasets, ePPID explains a higher percentage variance of protein evolutionary rate than PPID (Figure 1 and Additional file 1, Table S1). These results indicate that ePPID is a better predictor of protein evolutionary rate than PPID. In addition, our further analysis indicated that ePPID predicts evolutionary rate better than betweenness [46-48], another network centrality measure (the last column of Table 1).

We then found that Spearman rank correlations between ePPID and protein abundance are all statistically significant (see Additional file 1, Text S2), suggesting that protein abundance might be a confounding factor for the high correlations between ePPID and protein evolutionary rate. To address this question, we studied the percentage variance of protein evolutionary rate explained by ePPID when protein abundance is controlled for (the last column of Table 2). As can be seen, considerable percent variances of evolutionary rate explained by ePPID remain in all the six protein interaction datasets, with the exception of "DIP-CORE". In addition, partial Spearman correlation coefficient and corresponding statistical significance between ePPID and evolutionary rate (by controlling for protein abundance; the last column of Table 3) are reduced as compared to the original correlations (column 5 of Table 3) in all protein interaction datasets. However, the fact that these partial correlations all remain highly significant (the last column of Table 3) also suggests that ePPID makes an independent contribution to protein evolutionary rate. Moreover, with the exception of the "DIP-FULL" dataset, the partial correlations between ePPID and protein evolutionary rate are more significant than those between PPID and protein evolutionary rate after controlling for protein abundance (Table 3, the last column versus column 4), further indicating that ePPID is a better predictor of protein evolutionary rate than PPID. In fact, similar results (Additional file 1, Table S2 and S3) were found using three other protein evolutionary rate data (corresponding to different out-group controls, including *S.cer *vs *S.par*, *S.cer *vs *S.mik *and *S.cer *vs *S.bay*, see [49] for details). Mechanistically, we believe that permanent interactions impose more selective pressure on protein evolution than transient interactions, and protein evolutionary rate is more reflective of the number of a protein's permanent interaction partners as measured by ePPID.

### The effect of transient interactions on predicting protein evolutionary rate

With the co-expression information, our ePPID measure can filter out many transient interactions. Thus, we next wanted to study why removing transient protein interactions improved the correlation. In the "Y2H-union" dataset, we noticed that ePPID explains more than four times the variance of evolutionary rate than does PPID; however, in other datasets, the improvements are generally less than three times (Additional file 1, Table S1). This result suggests that ePPID has filtered out many transient protein interactions in the "Y2H-union" dataset, which may be the reason of lower percent variance of evolutionary rate explained by PPID. On the other hand, improvements are less dramatic in other datasets because transient protein interactions are less enriched. Consistent with this notion, our study on non-co-expressed protein interactions (see Additional file 1, Text S5 for details) suggested that transient interactions are most enriched in the "Y2H-union" dataset (46.1%) while least enriched in the "Combined-AP/MS" dataset (14.4%, column 4 of Additional file 1 Table S4), which is also consistent with the fact that transient protein interactions are less co-expressed than permanent co-complex associations [32]. In addition, the number of transient interaction partners of a protein even appears to be positively correlated with protein evolutionary rate (see Additional file 1, Text S5).

Since the "Y2H-union" dataset is enriched for transient physical interactions, ePPID in this dataset mainly filters out a protein's transient physical interactions and thus reflects the number of the protein's permanent physical interactions. In the "Combined-AP/MS" dataset which is enriched for permanent co-complex associations, ePPID mainly filters out a protein's transient co-complex associations and thus reflects the number of the protein's permanent co-complex associations. In the "Combined-AP/MS" dataset, ePPID may be overestimated due to indirect non-physical interactions (co-complex associations). Despite this effect, permanent interactions do place higher selective constrains on protein evolution than transient interactions do, further illustrating why our ePPID measure could better predict protein evolutionary rate.

However, the variance of protein evolutionary rate explained by ePPID is still the lowest in the "Y2H-union" dataset, which may be explained in three ways. First, ePPID cannot filter out all transient protein interactions, partly because of noise in the gene expression datasets we used. Second, Y2 H datasets may contain co-expressed protein pairs which are localized to different cellular compartments and seldom interact in nature. Third, ePPID may be underestimated based on incompleteness of Y2 H datasets [30,32], which is also reflected by the lowest average degree in the "Y2H-union" dataset (column 5 of Additional file 1, Table S4).

### Global study of ePPID and other genomic variables for protein evolutionary rate

A number of genomic variables, such as expression level [16,25,50-52], functional dispensability [25,53] and pleiotropic effect [54,55], are proposed to be associated with protein evolutionary rate. Also, these variables may have redundancy since they are correlated with each other. Therefore, we next attempted to determine the possible confounding effect of these variables on the correlations between ePPID and protein evolutionary rate.

For this purpose, we collected two expression-related variables, mRNA abundance and protein abundance; two function-related variables, gene dispensability and gene pleiotropy, which were measured by the associated number of GO biological process terms of each gene; and two network-related variables, ePPID and betweenness (see Methods). We then carried out a principal component regression [16,56] of protein evolutionary rate dN against the six predictor variables. The results for the "Y2H-union", "Combined-AP/MS" and "LC-multiple" datasets are summarized in Tables 4, 5 and 6, and those of the other three datasets are provided in Additional file 1, Table S6A-C.

**Table 4:** Principal component regression analysis on six predictor variables and protein evolutionary rate for 752 yeast proteins in the "Y2H-union" dataset.

	Principal Components						
	
	1	2	3	4	5	6	All
Percent variance explained in dN	34.13***	0.55	0.46	0.38	0.23	0.00	35.74***

Percent contributions							

mRNA abundance	**36.2**	1.2	**49.1**	13.3	0.0	0.2	
	
protein abundance	**37.3**	1.7	**50.7**	10.2	0.0	0.0	
	
gene dispensability	10.4	2.0	0.0	**37.2**	**49.9**	0.4	
	
gene pleiotropy	11.3	0.0	0.1	**38.9**	**49.2**	0.4	
	
ePPID	3.9	**45.4**	0.0	0.3	0.4	**49.9**	
	
betweenness	0.8	**49.6**	0.1	0.1	0.4	**49.0**	

Note: ^#^P < 0.01; *P < 10^-3^; **P < 10^-6^; ***P < 10^-9^. Bold indicates that the predictor variable contributes at least 20% to the corresponding principal component.

**Table 5:** Principal component regression analysis on six predictor variables and protein evolutionary rate for 723 yeast proteins in the "Combined-AP/MS" dataset.

	Principal Components						
	
	1	2	3	4	5	6	All
Percent variance explained in dN	27.26***	7.77***	1.11*	0.92^#^	0.83^#^	0.01	37.90***

Percent contributions							

mRNA abundance	**24.3**	9.0	14.6	**47.3**	0.3	4.4	
	
protein abundance	**22.8**	15.9	13.4	**41.3**	0.1	6.5	
	
gene dispensability	8.7	2.5	**20.8**	1.0	**63.2**	3.8	
	
gene pleiotropy	0.0	**37.2**	**40.1**	0.0	**20.6**	2.1	
	
ePPID	**24.5**	16.6	4.3	7.5	0.0	**47.0**	
	
betweenness	19.7	18.9	6.8	2.8	15.8	**36.1**	

Note: ^#^P < 0.01; *P < 10^-3^; **P < 10^-6^; ***P < 10^-9^. Bold indicates that the predictor variable contributes at least 20% to the corresponding principal component.

**Table 6:** Principal component regression analysis on six predictor variables and protein evolutionary rate for 639 yeast proteins in the "LC-multiple" dataset.

	Principal Components						
	
	1	2	3	4	5	6	All
Percent variance explained in dN	27.43***	6.90***	1.40*	0.94^#^	0.32	0.26	37.24***

Percent contributions							

mRNA abundance	**21.4**	**22.0**	**50.1**	6.0	0.0	0.5	
	
protein abundance	**20.7**	**21.7**	**44.3**	6.3	1.6	5.3	
	
gene dispensability	13.0	0.0	0.4	**65.6**	19.9	1.1	
	
gene pleiotropy	2.8	**25.6**	0.8	**22.0**	**48.6**	0.2	
	
ePPID	**25.9**	10.4	3.6	0.1	6.8	**53.2**	
	
betweenness	16.3	**20.3**	0.7	0.0	**23.0**	**39.7**	

Note: ^#^P < 0.01; *P < 10^-3^; **P < 10^-6^; ***P < 10^-9^. Bold indicates that the predictor variable contributes at least 20% to the corresponding principal component.

Results show that the first principal component explains much more variance of protein evolutionary rate than the other components in all the six datasets. Thus, in the following, we focus on the first principal component to study the percentage contribution of ePPID. In the "Combined-AP/MS", "LC-multiple", "Updated-HC", "DIP-CORE" and "DIP-FULL" datasets, the contribution of ePPID to the first principal component is more than that of all other variables. In the "Y2H-union" dataset, the ePPID contribution is more than betweenness, but less than the other four variables. Consistently, the independent contribution of ePPID to the total variance of protein evolutionary rate dN explained by all the six principal components in most datasets is comparable to that of the expression-related variables of mRNA abundance and protein abundance (Additional file 1, Table S7). Similar results were obtained when using codon adaptation index (CAI) [36] instead of mRNA abundance or protein abundance to perform analysis (see Additional file 1, Text S6). Furthermore, when using three expression-related variables of mRNA abundance, protein abundance and CAI to perform analysis, ePPID still has a considerable and independent contribution to protein evolutionary rate (see Additional file 1, Text S6). We therefore concluded that ePPID has an important and independent effect on protein evolutionary rate, confirming the importance and novelty of our proposed new measure.

### Proteins with more co-expressed partners evolve more slowly than those with less co-expressed partners

**Figure 3:**
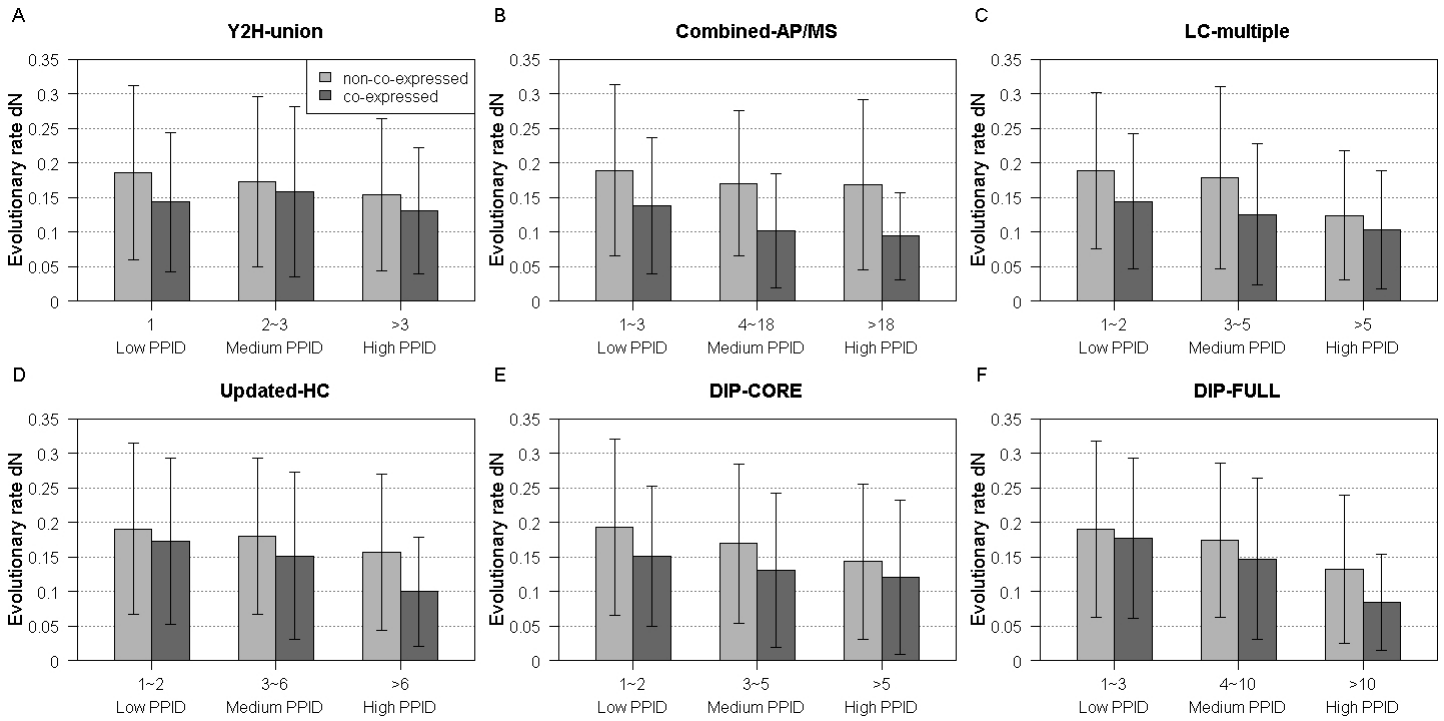
**Co-expressed proteins evolve more slowly than non-co-expressed proteins**. Protein evolutionary rate dN (y-axis) of non-co-expressed proteins (gray bars) and co-expressed proteins (dark gray bars) is shown as a function of PPID (x-axis). For the purpose of a detailed comparison, non-co-expressed and co-expressed proteins are further grouped into three bins (see Methods for details) according to their PPID values. (A) "Y2H-union" (B) "Combined-AP/MS" (C) "LC-multiple" (D) "Updated-HC" (E) "DIP-CORE" (F) "DIP-FULL".

Since the evolutionary rate differences between different types of hub proteins have also been debated [18-23], we then wanted to study this problem by integrating co-expression data. We divided proteins into low, medium and high PPID bins and classified proteins into co-expressed and non-co-expressed proteins (see Methods). As a result, co-expressed proteins were found to have a significantly lower evolutionary rate than non-co-expressed proteins in each bin (Figure 3 and Additional file 1, Table S8A-C). At the same time, it should be noted that no significant difference in the high-PPID and medium-PPID bins was observed for the "Y2H-union" dataset. However, the observed significantly lower evolutionary rate of co-expressed proteins in each bin may be confounded by PPID, but our further analysis did not support this notion (see Additional file 1, Text S7). These results further indicate that proteins with more permanent interaction partners are under higher evolutionary pressure and thus evolve more slowly.

### ePPID helps the understanding of protein evolutionary rate in the Structural Interaction Network dataset

Protein interactions can also be studied from a structural perspective. We next applied our co-expressed and non-co-expressed protein classification method to hub proteins (with ≥5 protein interaction partners) in the "SIN" dataset [22] and studied the relationship between ePPID and the number of binding interfaces. As shown in Additional file 1, Table S9, non-co-expressed hubs correspond mostly to singlish-interface hubs, whereas co-expressed hubs correspond mostly to multi-interface hubs (Fisher's exact test, P = 1.63e-3), suggesting that co-expression may be a characteristic of proteins with many distinct interfaces, which enable these proteins to interact together permanently. To test our hypothesis, we studied whether a correlation exists between ePPID and the number of binding interfaces from [22]. As it turned out, the correlation is highly significant (Spearman rank correlation rho = 0.408, P = 4.40e-8). Considering the difficulties in obtaining protein structure data, this result suggests that the ePPID measure is a good predictor of the number of binding interfaces of a protein.

**Figure 4:**
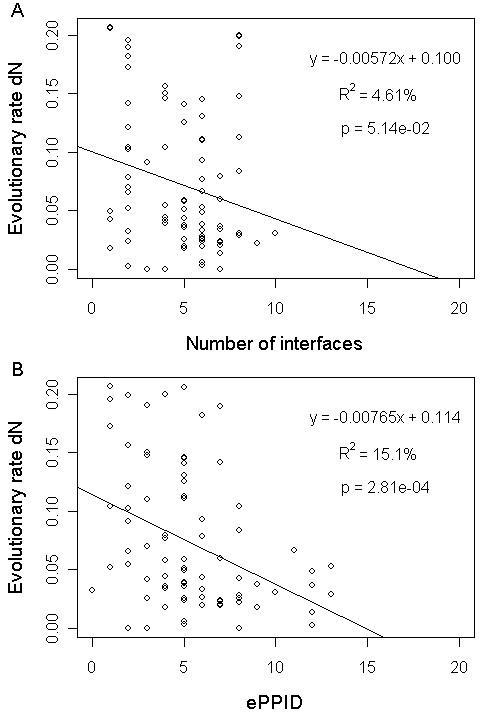
**Relationship of protein evolutionary rate with the number of interfaces and ePPID**. Scatter plots of protein evolutionary rate dN versus (A) the number of interfaces and (B) ePPID together with the linear regression fit in the "SIN" dataset.

It is reported that protein evolutionary rate is actually more reflective of the number of distinct binding interfaces [22]. Yet we found that the correlation between the number of binding interfaces and protein evolutionary rate for hub proteins is not significant (Spearman rank correlation rho = -0.211, P = 0.0561) at the statistical significance level of 0.05. On the other hand, the correlation between ePPID and protein evolutionary rate for hub proteins is highly significant (Spearman rank correlation rho = -0.399, P = 1.89e-4). Similar results were obtained when statistical significance of the correlations is assessed by linear regression (Figure 4). Since ePPID explains the variance of protein evolutionary rate over three times higher than does the number of binding interfaces, we conclude that ePPID predicts protein evolutionary rate better than the number of binding interfaces. These results also implied that it is important to clearly distinguish permanent interfaces from transient interfaces when counting the number of a protein's distinct binding interfaces, because permanent and transient interfaces may contribute differently to protein evolutionary rate [37,38].

**Figure 5:**
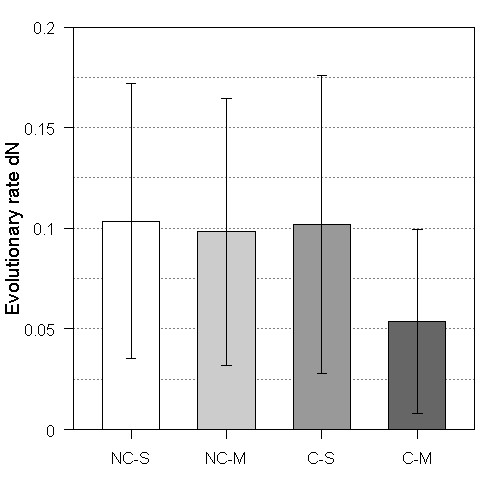
**Effects of permanent and transient interfaces on protein evolutionary rate**. Mean and standard deviation of protein evolutionary rate dN in the "SIN" dataset are shown for non-co-expressed proteins with single interface (NC-S, in a total of 10 proteins), non-co-expressed proteins with multiple interfaces (NC-M, in a total of 12 proteins), co-expressed proteins with single interface (C-S, in a total of 10 proteins) and co-expressed proteins with multiple interfaces (C-M, in a total of 51 proteins). C-M evolve significantly more slowly than NC-S (P = 1.05e-02), NC-M (P = 1.34e-02) and C-S (P = 2.29e-02). Other comparisons did not yield significant results (i.e., P > 0.05). P is calculated by one-sided Wilcoxon rank sum test.

It is also reported that multi-interface hubs have lower evolutionary rate than singlish-interface hubs (one-sided Wilcoxon rank sum test, P = 8.66e-3) [22]. Therefore, we next studied the effect of permanent and transient interfaces on protein evolutionary rate by integrating co-expression data. The hub proteins in the "SIN" dataset are grouped (see Methods) into four classes: non-co-expressed singlish-interface hubs, non-co-expressed multi-interface hubs, co-expressed singlish-interface hubs and co-expressed multi-interface hubs. The interfaces of co-expressed and non-co-expressed hubs are assumed to be permanent and transient, respectively. We found that non-co-expressed singlish-interface hubs, non-co-expressed multi-interface hubs and co-expressed singlish-interface hubs evolve at a similar rate. On the other hand, co-expressed multi-interface hubs evolve at a significantly lower rate (Figure 5), indicating that hubs with more permanent interfaces are subject to higher evolutionary constraints and thus evolve more slowly. This result is in clear contrast with the finding of [23] where the evolutionary rate difference between multi-iMotif hubs and singlish-iMotif hubs is not found to be significant. Thus, we conclude that the difference in evolutionary rate between singlish-interface (singlish-iMotif) hubs and multi-interface (multi-iMotif) hubs is better clarified by the ePPID measure. In fact, more significant results were obtained when using the "Updated-SIN" dataset, which has a relatively larger size than the "SIN" dataset (see Additional file 1, Text S8). Finally, we note that all the above results can be replicated if we define protein evolutionary rate as dN/dS or dN/dS' (ratio of non-synonymous substitutions to adjusted synonymous substitutions; data not shown).

### Application of ePPID in human data

**Figure 6:**
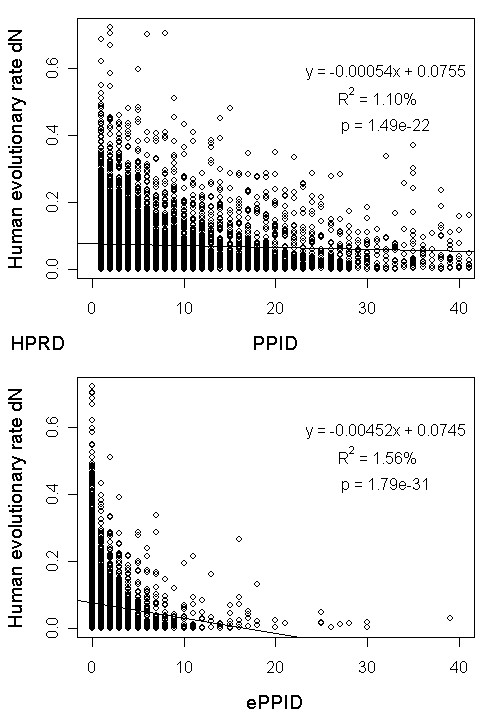
**Relationship of human protein evolutionary rate dN with PPID and ePPID**. Scatter plots of human protein evolutionary rate dN versus PPID (upper panel) and ePPID (bottom panel) together with the linear regression fit in the "HPRD" dataset.

To see whether our result for yeast can be obtained in other species, we obtained the relevant data for human and computed ePPID for each protein (see Additional file 1, Text S9 for details). As a result, we found that the percent variance of evolutionary rate explained by ePPID is higher than that explained by PPID (Figure 6). The Spearman rank correlation between PPID and protein evolutionary rate is -0.172 and its P-value is 1.24e-58, while the Spearman rank correlation between ePPID and protein evolutionary rate is -0.206 and its P-value is 2.71e-83. Thus, we concluded that our result also holds in human and will study if it can be obtained in more species in the future.

## Discussion

DNA mutations, especially those in protein-coding regions, are a driving force of biological novelties. Understanding protein evolutionary rate is thus an important topic. Along with rapid progress in high-throughput methods in recent years, it is possible to study protein evolutionary rate from many perspectives. Protein interactions, which are believed to exert an important selective pressure on protein evolution at the functional level, have been heavily studied in recent years. However, owing to the complexity in experimental setup and the biological system itself, controversial results have led investigators to debate the association between protein-protein interaction degree (PPID) and protein evolutionary rate.

Proteins with higher PPID are assumed to have a greater proportion of residues involved in interactions and thus evolve more slowly than proteins with lower PPID [6,9]. This assumption was supported by the fact that ePPID, which measures the number of a protein's permanent interaction partners, could better predict protein evolutionary rate. In Y2 H datasets which are enriched for transient physical interactions, ePPID mainly filters out a protein's transient physical interactions and thus reflects the number of the protein's permanent physical interactions. Though the filtered interactions of a protein contribute to the PPID of the protein, they may not contribute to the proportion of the protein's residues involved in interactions (i.e., the protein tends to interact with its different filtered partners through the same binding interface). As demonstrated by our results, transient physical interactions on average indeed exert lower selective constraints on protein evolution. On the other hand, in AP/MS-related datasets, the protein pairs may not physically interact in nature; rather, they appear in the same protein complexes. In such datasets, ePPID mainly filters out a protein's transient co-complex associations and thus reflects the number of the protein's permanent co-complex associations. Though the filtered interactions of a protein may contribute to the proportion of the protein's residues involved in interactions (i.e., the protein may interact with its filtered partners through multiple distinct transient interfaces), they do not contribute to the proportion of the protein's residues involved in permanent interactions. Thus, they should also be filtered out because they may not exert higher selective constraints on protein evolution, which is also demonstrated by our results. However, Y2 H datasets are more likely to contain false negatives (incompleteness of Y2 H datasets) and ePPID in such datasets may be underestimated, whereas AP/MS-related datasets are more likely to contain false positives (indirect non-physical interactions) and ePPID in such datasets may be overestimated. We hope to study this effect when more reliable and complete protein interaction data become available in the future. Despite this slight difference, our results demonstrated a clearer role of protein interaction degree as a constraint on protein evolution.

## Conclusions

In this work, we performed extensive studies to identify how protein interactions, as measured by PPID, affect protein evolutionary rate. By carefully comparing experimental setups, we observed that Y2 H assays may have introduced a considerable amount of transient protein interactions. On this basis, we hypothesized that the difference in experimental methods contributes to our inability to clearly define how PPID affects protein evolutionary rate. This hypothesis was confirmed by introducing a new protein interaction degree measure, the co-expressed protein-protein interaction degree (ePPID). Since ePPID is a measure that integrates protein interactions with gene co-expression information, it can filter out many transient protein interactions. As a result, ePPID gives a better prediction of protein evolutionary rate than PPID in the various protein interaction datasets tested. The relationship between ePPID and protein evolutionary rate is also robustly significant in all protein interaction datasets, which was not possible when using PPID in previous studies. We also investigated the redundancy between several variables that may affect protein evolutionary rate against the contribution of ePPID and found that ePPID makes an independent contribution to protein evolutionary rate. This result suggests the novelty of ePPID as an important determinant of protein evolutionary rate. Moreover, the application on hub proteins in the Structural Interaction Network provides further support that ePPID also gives a better prediction of protein evolutionary rate than the number of distinct binding interfaces and clarified the slower evolution of co-expressed multi-interface hub proteins over that of other hub proteins.

In summary, our work provides a new protein interaction degree measure by integrating protein interaction datasets with gene expression datasets. This new measure has, at least in part, resolved the longstanding debates on the role of protein interactions in affecting protein evolutionary rate. Finally, we have found that this result also holds in human. We will study if this can be observed in more species in the future.

## Methods

### Protein interaction datasets

To study the effect of experimental bias, reliability and completeness, we collected nine different yeast protein interaction datasets. We used the "Y2H-union", "Combined-AP/MS" and "LC-multiple" datasets to represent typical protein interaction datasets obtained from Y2 H assays, the AP/MS method and literature curation, respectively. To account for data quality (confidence), we also used the filtered yeast interactome ("FYI"), the Structural Interaction Network ("SIN"), "DIP-CORE" and the updated high-confidence dataset ("Updated-HC") as high-confidence datasets. In addition, the "SIN" dataset was also used to study the evolutionary rate of hub proteins through a mechanistic perspective. In contrast, we used the "DIP-FULL" and "Eight-union" datasets to account for completeness. The nine datasets are listed below.

1) Y2H-union: the union of three high-throughput Y2 H datasets: Uetz-screen [57], Ito-core [58] and CCSBYI1 [32].

2) Combined-AP/MS: an integrated dataset [59] of two high-throughput AP/MS datasets [60,61].

3) LC-multiple: a protein interaction dataset based on the literature. Each protein interaction must have been curated from ≥2 different publications [62].

4) FYI: the filtered yeast interactome obtained from [24].

5) SIN: the Structural Interaction Network dataset obtained from [22].

6) Updated-HC: the updated high-confidence dataset obtained from [21].

7) DIP-CORE: the core dataset derived from the database of interacting proteins (DIP) [42,43].

8) DIP-FULL: the full dataset derived from the database of interacting proteins (DIP) [43].

9) Eight-union: the union of the above eight datasets.

The network properties of the nine datasets are summarized in Additional file 1, Table S4. The overlap of interactions between the nine datasets was shown in Additional file 1, Table S5A-C. We analyzed the six protein interaction datasets 1), 2), 3), 6), 7) and 8) in the main text and the analysis results of the other three 4), 5) and 9) were provided in Additional file 1, Text S1.

### Data sources for correlation and principal component regression analyses

Evolutionary rate data (non-synonymous substitutions per site dN), which is based on the four-way yeast species alignments for 3,036 *Saccharomyces cerevisiae *genes, were obtained from [25]. Specifically, orthologous genes were aligned by using ClustalW [63] and dN was then estimated using PAML [64]. Three other protein evolutionary rate data (corresponding to different out-group controls, including *S.cer *vs *S.par*, *S.cer *vs *S.mik *and *S.cer *vs *S.bay*) were obtained from [49]. mRNA abundance data were obtained from [35]. Protein abundance data were obtained from [33]. Codon adaptation index (CAI), which measures synonymous codon usage bias [65], was obtained from [36]. Gene dispensability, measured by the average growth rates of homozygous deletion strains, was obtained from [66]. The associated number of GO biological process terms of a gene [67], used as a measure of gene pleiotropy, was obtained from the *Saccharomyces *Genome Database [68]. Protein betweenness, measured by the total number of shortest paths going through a protein in a protein interaction network [46,47], was calculated by using R [69] with the package "igraph" [70].

Partial correlation analysis is frequently used to determine the confounding effect of variables such as protein abundance on the relationship between PPID and protein evolutionary rate [6,9,13-15,25,51,71]. It is also reported that principal component regression analysis can provide a complementary analysis to partial correlation analysis [72] and that the relative contributions of the transformed predictors to the overall regression model can be evaluated independently and reliably [16]. In this paper, we performed both principal component regression and partial correlation analyses to understand protein evolutionary rate. Principal component regression was performed by using R with the package "pls" [73]. Before carrying out the principal component analysis, all variables were log transformed, except dispensability, and all predictor variables were standardized to zero mean and unit variance. It should be noted that a small constant of 0.001 was added to protein evolutionary rate dN as described in [25] to avoid zero values. A small constant of 0.1 was added to ePPID and betweenness to avoid zero values and we demonstrated that our results were not sensitive to these constants (see Additional file 1, Text S10). The statistical significance levels were determined according to Drummond et al. [16]. Partial correlation analysis was performed by using R with the function provided by Kim and Yi [72]. The method for computing the percentage variance of protein evolutionary rate explained by ePPID when protein abundance is controlled for was as follows. First, we performed a linear regression of protein evolutionary rate dN against Log(protein abundance) and obtained dN = f(Log(protein abundance)). We then computed the residue, i.e., dN_residue = dN-f(Log(protein abundance)). Finally, we performed a linear regression of the residue against ePPID: dN_residue = g(ePPID), and obtained the explained variance.

### Gene expression datasets

We collected ten gene expression datasets [74-83], each with more than 50 samples (conditions), from the Yeast Functional Genomics Database [84] and the *Saccharomyces *Genome Database [68]. Genes with missing value in >30% of the samples in a dataset were removed. Remaining missing values were imputed by the KNN impute algorithm with K = 10 using Euclidean distance [85], and technical replicates (i.e., spot repeats and dye swaps) were averaged.

### Construction of gene co-expression networks

Pearson correlation coefficient (PCC) *r *is used as a similarity measure between the expression profiles of two genes. The PCC *r *was then converted into *z*-score using Fisher transformation:

$$z(r)=\frac{\sqrt{n-3}}{2}\log\frac{1+r}{1-r}$$

which approximately follows a standard normal distribution under the hypothesis of independence, where *n *is the sample size. We only considered positive correlations since they are reported to be more reflective of functional similarity than negative correlations [86]. Next, P-values were obtained for the null hypothesis of no positive correlations and were corrected for multiple hypothesis testing by using false discovery rate (FDR) control procedure [87], and the adjusted P-values were set at the threshold of 0.001 per dataset (FDR = 0.001). In addition, we only considered those pairs that are among the top 10 percent of all possible correlations (PER = 10%) to avoid introducing too many high correlations. Our two-stage threshold selection procedure is similar to the procedure that controls both statistical significance and biological significance in [86,88] and we demonstrated that our results were not sensitive to different thresholds of FDR and PER (see Additional file 1, Text S11). For each gene expression dataset, two genes are declared to be co-expressed if their correlation coefficient is above the thresholds of both FDR and PER.

### Definition of co-expressed protein-protein interaction degree (ePPID) and classification of proteins

In a given protein interaction dataset, let PPI_g _denote the set of interaction partners of a given protein g. By filtering out the potential transient interaction partners in PPI_g_, we want to integrate gene co-expression in a way that allows us to identify the partners with which protein g can permanently interact. To explain, we can calculate the z-scores of co-expression between protein g and all genes in PPI_g _for each gene expression dataset. Let ePPI_g_(i) denote the number of genes that are found to be significantly co-expressed with gene g in gene expression dataset i (i = 1,2,...,10; see previous paragraph). The co-expressed protein-protein interaction degree (ePPID) of protein g is then defined as ePPID_g _= max(ePPI_g_(i); i = 1,2,...,10). In addition, we tried other co-expressed protein-protein interaction degree measures to demonstrate the robustness of our results (see Additional file 1, Text S4).

To study whether evolutionary rate differences between different types of proteins can be better clarified by distinguishing permanent interactions from transient interactions, we divided proteins into low, medium and high PPID bins, with the high-PPID bin containing about 20% of the total number of proteins (also called hubs) in each protein interaction dataset. Similar to the concept behind the date and party hub definition in [24], we further grouped proteins into two classes. A protein was defined as co-expressed if the ratio of ePPID to PPID (ePPID/PPID) is ≥0.5; otherwise, it was defined as a non-co-expressed protein.

To study the contribution of permanent and transient interfaces to protein evolutionary rate in the "SIN" dataset, the ePPID of hub proteins in the "SIN" dataset was calculated similarly, and the hub proteins were further grouped into four classes: non-co-expressed singlish-interface hubs, non-co-expressed multi-interface hubs, co-expressed singlish-interface hubs and co-expressed multi-interface hubs by taking intersections.

## Abbreviations

APCC: average Pearson correlation coefficient; AP/MS: affinity purifications followed by mass spectrometry; CAI: codon adaptation index; dN: non-synonymous substitution rate; ePPID: co-expressed protein-protein interaction degree; FDR: false discovery rate; GO: Gene Ontology; PCC: Pearson correlation coefficient; PER: percent of all possible correlations; PPID: protein-protein interaction degree; Y2H: yeast two-hybrid.

## Authors' contributions

KP and XM conceived and designed the experiment; HS and XM supervised the experiment; KP, CC, ZX, HS and XM conducted the experiment and wrote the manuscript. All authors have read and approved the final manuscript.

## Supplementary Material

Additional file 1 **Supplementary texts, figures and tables**. This file contains Supplementary Texts S1-S11, Figures S1-S4 and Tables S1-S38. Click here for file
