# Supplementary material for: Understanding protein evolutionary rate by integrating gene co-expression with protein interactions
Source: BMC Syst Biol. 2010 Dec 30;4:179. doi: 10.1186/1752-0509-4-179 (PMC3022652; doi:10.1186/1752-0509-4-179)
Supplement: Additional file 1 — Supplementary texts, figures and tables. This file contains Supplementary Texts S1-S11, Figures S1-S4 and Tables S1-S38. [file 1752-0509-4-179-S1.DOC]

# Additional file 1 contains:

**Supplementary Texts S1-S11**

**Supplementary References**

**Supplementary Figures S1-S4**

**Supplementary Tables S1-S38**

# Supplementary Texts

# Text S1 – The analysis results of the “FYI”, “SIN” and “Eight-union” datasets

Besides the six protein interaction datasets we analyzed in the main text, we also analyzed the “FYI”, “SIN” and “Eight-union” datasets. For the three protein interaction datasets, statistically significant results of the negative correlations between PPID and protein evolutionary rate were also found by both linear regression fit (upper panel of Additional file 1, Figure S1) and Spearman rank correlation (column 3 of Additional file 1, Table S10). For the three datasets, considerable percent variances of evolutionary rate explained by PPID remain when protein abundance is controlled for (column 4 of Additional file 1, Table S11), and the partial Spearman correlation coefficients between PPID and protein evolutionary rate are still significant after controlling for protein abundance (column 4 of Additional file 1, Table S12).

For the three protein interaction datasets, it can be seen from the bottom panels of Additional file 1, Figure S1 that the ePPID measure has statistically significant negative correlation coefficients with protein evolutionary rate. As shown in Additional file 1, Table S10 (column 4 versus column 3), it is clear that the statistical significance obtained by ePPID is better than that obtained by PPID. Accordingly, ePPID explains a higher percent variance of protein evolutionary rate than PPID (Additional file 1, Figure S1 and Table S1). For the three datasets, considerable percent variances of evolutionary rate explained by ePPID remain when protein abundance is controlled for (the last column of Additional file 1, Table S11), and the partial Spearman correlation coefficients between PPID and protein evolutionary rate are still significant after controlling for protein abundance (the last column of Additional file 1, Table S12).

In the “Eight-union” dataset, the contribution of ePPID to the first principal component is more than that of all other variables (Additional file 1, Table S6F). In the “FYI” dataset, the ePPID contribution is slightly less than mRNA abundance and protein abundance, but more than the other three variables (Additional file 1, Table S6D). In the “SIN” dataset, the ePPID contribution is less than mRNA abundance and protein abundance, but more than the other three variables (Additional file 1, Table S6E).

For the three protein interaction datasets, co-expressed proteins were found to have a significantly lower evolutionary rate than non-co-expressed proteins in each bin (Additional file 1, Figure S2 and Table S8A-C). At the same time, it should be noted that no significant difference in the medium-PPID bin was observed for the “FYI” dataset.

# Text S2 – Correlation of PPID and ePPID with protein abundance

We next studied if the protein abundance effect may account for the above significant difference between the Y2H and other datasets. We computed Spearman rank correlations between PPID and protein abundance. We found that significant correlation coefficients exist in the “Combined-AP/MS”, “Updated-HC”, “DIP-CORE”, “DIP-FULL”, “FYI” and “Eight-union” datasets (column 3 of Additional file 1, Table S13), which at least partly contain protein interactions detected by the AP/MS method and thus may have abundance effects. In contrast, no significant correlation was found in the “Y2H-union” dataset, which is not subject to abundance effect. This result is consistent with [S1-S4] and suggests that protein abundance may be a confounding factor for the significant relationship between PPID and protein evolutionary rate in AP/MS-related datasets. However, in the “LC-multiple” and “SIN” datasets, which are less affected by protein abundance, no significant correlation coefficients can be found between PPID and protein abundance. This result suggests that the PPID measure may contain independent information for protein evolutionary rate.

Since ePPID is derived from PPID, which is found to be correlated with protein abundance, we also investigated the relationship between ePPID and protein abundance. Results showed that correlations between ePPID and protein abundance are all statistically significant (P<0.05, column 4 of Additional file 1, Table S13). Also, all of these correlations are more significant than those between PPID and protein abundance (Additional file 1, Table S13, column 4 versus column 3), suggesting that proteins with high ePPID scores are generally more abundant, possibly reflecting their important roles in cellular functions [S5].

# Text S3 – Some proteins which tend to have high ePPID but low APCC in real data

To further compare ePPID and APCC, some hub proteins (with ≥7 protein interaction partners) in the “Updated-HC” dataset were shown in Additional file 1, Table S14. These hub proteins tend to have high ePPID but low APCC in gene expression dataset [S6]. When using APCC to classify hubs, these hub proteins will be classified as date hubs. However, the ePPID of these hub proteins are high and these hubs are more like party hubs, indicating that the APCC score of a protein is sensitive to a high variance of Pearson correlation coefficient (PCC) scores between the protein and its interaction partners.

# Text S4 – The analysis results of another two co-expressed protein-protein interaction degree measures

As described in Methods, the co-expressed protein-protein interaction degree (ePPID) of protein g is defined as ePPID_g_=max(ePPI_g_(i); i=1,2,…,10). Here, we defined another two co-expressed protein-protein interaction degree measures. The first one is defined as ePPID_avg_=mean(ePPI_g_(i); i=1,2,…,10). The second one is defined as ePPID_sec_=sec-max(ePPI_g_(i); i=1,2,…,10), where sec-max denotes the second maximal value of ePPI_g_(i) (i=1,2,…,10). The two measures are more robust against the noise inherent in co-expression information, and similar results were obtained when using them to perform analysis (Additional file 1, Tables S15 and S16).

# Text S5 – Non-co-expressed protein-protein interaction degree (nePPID) tend to positively correlate with protein evolutionary rate

To further study the effect of transient protein interactions on evolutionary rate, we then proposed a non-co-expressed protein-protein interaction degree (nePPID) to approximately estimate the number of partners with which a protein may transiently interact. Specifically, from each protein interaction dataset, we removed protein interactions in which both partners were co-expressed in any of the ten gene expression datasets. The remaining protein interactions are termed non-co-expressed protein interactions, and the resultant protein interaction dataset is used to approximate the transient protein interaction network. The non-co-expressed protein-protein interaction degree (nePPID) is then defined as the number of interaction partners a protein has in this transient protein interaction network. Interestingly, while the negative correlations between nePPID and evolutionary rate are less significant than those between PPID and evolutionary rate in the “DIP-FULL” and “Eight-Union” datasets, we even found positive correlations between nePPID and evolutionary rate in the“Y2H-union”, “Combined-AP/MS”, “LC-multiple”, “FYI”, “SIN”, “Updated-HC” and “DIP-CORE” datasets (Additional file 1, Table S17). This finding further suggests that enriched transient interactions of a protein may have been a confounding factor leading other investigators to draw inconsistent conclusions about their effect on evolutionary rate.

# Text S6 – The results of three other principal component regression analyses

Besides a principal component regression analysis using two expression-related variables of mRNA abundance and protein abundance, we also used codon adaptation index (CAI) instead of mRNA abundance or protein abundance to perform analysis. The results of principal component regression analysis using mRNA abundance and CAI for the nine datasets are summarized in Additional file 1, Table S18A-I. Results show that the first principal component explains much more variance of protein evolutionary rate than the other components in all the nine datasets. In the “LC-multiple”, “Updated-HC”, “DIP-CORE”, “DIP-FULL” and “Eight-union” datasets, the contribution of ePPID to the first principal component is more than that of all other variables. In the “Combined-AP/MS” dataset, the ePPID contribution is slightly less than mRNA abundance, but more than the other four variables (Additional file 1, Table S18B). In the “FYI” dataset, the ePPID contribution is slightly less than mRNA abundance and CAI, but more than the other three variables (Additional file 1, Table S18G). In the “SIN” dataset, the ePPID contribution is less than mRNA abundance and CAI, but more than the other three variables (Additional file 1, Table S18H). In the “Y2H-union” dataset, the ePPID contribution is more than betweenness, but less than the other four variables (Additional file 1, Table S18A). Consistently, the independent contribution of ePPID to the total variance of protein evolutionary rate dN explained by all the six principal components in most datasets is comparable to that of the expression-related variables of mRNA abundance and CAI (Additional file 1, Table S19).

The results of principal component regression analysis using protein abundance and CAI for the nine datasets are summarized in Additional file 1, Table S20A-I. Results show that the first principal component explains much more variance of protein evolutionary rate than the other components in all the nine datasets. In the “LC-multiple”, “Updated-HC”, “DIP-CORE”, “DIP-FULL” and “Eight-union” datasets, the contribution of ePPID to the first principal component is more than that of all other variables. In the “Combined-AP/MS” dataset, the ePPID contribution is slightly less than protein abundance, but more than the other four variables (Additional file 1, Table S20B). In the “FYI” dataset, the ePPID contribution is slightly less than protein abundance and CAI, but more than the other three variables (Additional file 1, Table S20G). In the “SIN” dataset, the ePPID contribution is less than protein abundance and CAI, but more than the other three variables (Additional file 1, Table S20H). In the “Y2H-union” dataset, the ePPID contribution is more than betweenness, but less than the other four variables (Additional file 1, Table S20A). Consistently, the independent contribution of ePPID to the total variance of protein evolutionary rate dN explained by all the six principal components in most datasets is comparable to that of the expression-related variables of protein abundance and CAI (Additional file 1, Table S21).

In addition, we also used three expression-related variables of mRNA abundance, protein abundance and CAI to carry out a principal component regression analysis. The results for the nine datasets are summarized in Additional file 1, Table S22A-I. Results show that the first principal component explains much more variance of protein evolutionary rate than the other components in all the nine datasets. In all the nine datasets except “Y2H-union”, the contribution of ePPID to the first principal component is less than that of mRNA abundance, protein abundance and CAI, but more than that of the other three variables. In the “Y2H-union” dataset, the ePPID contribution is more than betweenness, but less than the other five variables (Additional file 1, Table S22A). Consistently, the independent contribution of ePPID to the total variance of protein evolutionary rate dN explained by all the seven principal components in most datasets is slightly less than or less than that of the expression-related variables of mRNA abundance, protein abundance and CAI, but more than that of the other three variables (Additional file 1, Table S23).

As pointed out in [S7, S8], principal component regression analysis tends to overestimate the contribution of correlated predictor variables and underestimate that of other predictor variables. Since three expression-related variables of mRNA abundance, protein abundance and CAI are highly correlated with each other, principal component regression analysis may overestimate the contribution of these three expression-related variables and underestimate that of the other four predictor variables. This may explain why we observed that ePPID has a slightly less independent contribution to protein evolutionary rate when using three expression-related variables to perform analysis. Nevertheless, ePPID still has an important and independent effect on protein evolutionary rate. We note that *n* in Additional file 1, Table S7, S19, S21 and S23 is the number of proteins for which all seven predictor variables and protein evolutionary rate are available. In each protein interaction dataset, we used the same *n* to perform analysis, because we want to show that different results obtained by principal component regression analysis are only due to different expression-related variables used but not due to different numbers of proteins used.

# Text S7 – The results of analysis of covariance of protein evolutionary rate

Although co-expressed proteins were found to have a significantly lower evolutionary rate than non-co-expressed proteins in high, medium and low bins, we note that in each bin co-expressed proteins may have higher PPID than non-co-expressed proteins. Thus, PPID could be a confounding factor for the difference in evolutionary rate between co-expressed and non-co-expressed proteins. To study this issue, we performed analysis of covariance (ANCOVA) of protein evolutionary rate dN, predicted by co-expressed versus non-co-expressed proteins with PPID as a covariate. It can be seen from the last column of Additional file 1, Table S24A-C that the statistical significance for the effect of co-expressed versus non-co-expressed proteins is still significant in each bin in most datasets when controlling for the covariate PPID. We note, on the other hand, some exceptions where statistical difference is not significant: both high- and medium-PPID bins in the “Y2H-union” dataset; the high-PPID bin in the “LC-multiple” dataset; the high-PPID bin in the “DIP-CORE” dataset; the medium-PPID bin in the “FYI” dataset; and the low-PPID bin in the “SIN” dataset. Notwithstanding, these results further indicate that proteins with more permanent interaction partners are under higher evolutionary pressure and thus evolve more slowly.

# Text S8 – The analysis results of the “Updated-SIN” dataset

In addition to the “SIN” dataset, we also analyzed the relationship of protein evolutionary rate with PPID and ePPID in the “Updated-SIN” dataset, which has a relatively larger size, including 1,178 proteins and 2,195 interactions. The “Updated-SIN” dataset was obtained from http://networks.gersteinlab.org/structint. The Additional file 1, Tables S25 to S29 and Figure S3 show that the results of the “Updated-SIN” dataset are similar to those of the “SIN” dataset.

Based on the information given for the “Updated-SIN” dataset in Additional file 1, Table S30, non-co-expressed hubs correspond mostly to singlish-interface hubs, whereas co-expressed hubs correspond mostly to multi-interface hubs (Fisher’s exact test, P=6.08e-8). This result is more significant than the corresponding result in the “SIN” dataset. We also computed the average evolutionary rate of non-co-expressed singlish-interface hubs, non-co-expressed multi-interface hubs, co-expressed singlish-interface hubs and co-expressed multi-interface hubs in the “Updated-SIN” dataset. We found that non-co-expressed singlish-interface hubs, non-co-expressed multi-interface hubs and co-expressed singlish-interface hubs evolve at a similar rate. On the other hand, co-expressed multi-interface hubs evolve at a significantly lower rate (Additional file 1, Figure S4), and this result is more significant than the corresponding result in the “SIN” dataset.

# Text S9 – Human data

The human protein interaction dataset with 9,514 proteins and 36,985 interactions, was obtained from the Human Protein Reference Database (HPRD) [S9]. The human protein evolutionary rate (the non-synonymous substitution rate dN) based on the human and mouse homologs was obtained from NCBI HomoloGene [S10]. To prevent possible contamination by paralogous genes, we only considered one-to-one mapped orthologous pairs as Tu et al. [S11]. Two gene expression datasets of normal human tissues [S12] and the human cell cycle [S13] were obtained from the Stanford Microarray Database [S14]. Technical replicates were averaged, and genes with missing value in >30% of the samples in a dataset were removed. Two human gene co-expression networks were constructed by using thresholds FDR=0.001 and PER=0.1, and ePPID was then defined as the maximal number of co-expressed interaction partners of a given protein in the two gene expression datasets.

# Text S10 – The results of principal component regression analysis are not sensitive to different constants added to avoid zero values

In this work, a small constant of 0.1 was added to ePPID and betweenness to avoid zero values when performing principal component regression analyses of protein evolutionary rate dN against the six predictor variables. To study how our conclusions could be affected by this parameter, we tried to use constants 0.2 and 0.05. As can be seen from Additional file 1, Tables S31A-C to S36A-C, our conclusion is not affected by such a parameter. In addition, we also found that our conclusion is not affected by the log-transform (Additional file 1, Tables S31D to S36D).

# Text S11 – The effect of co-expression thresholds on the relationship between ePPID and protein evolutionary rate

In the main text, we used FDR=0.001 and PER=0.1 to construct ten gene co-expression networks to define ePPID. To study how these thresholds affect our conclusion, here we tried different thresholds: FDR=0.002 and PER=0.2; FDR=0.0005 and PER=0.05. As can be seen from Additional file 1, Tables S37 and S38, the correlation coefficients with their statistical significances, and the percent variances of evolutionary rate explained by ePPID are essentially the same, indicating that our conclusion is not sensitive to thresholds of FDR and PER.

# Supplementary References

S1. Bloom JD, Adami C: **Apparent dependence of protein evolutionary rate on number of interactions is linked to biases in protein-protein interactions data sets.** *BMC Evol Biol* 2003, **3:**21.

S2. von Mering C, Krause R, Snel B, Cornell M, Oliver SG, Fields S, Bork P: **Comparative assessment of large-scale data sets of protein-protein interactions.** *Nature* 2002, **417:**399-403.

S3. Bjorklund AK, Light S, Hedin L, Elofsson A: **Quantitative assessment of the structural bias in protein-protein interaction assays.** *Proteomics* 2008, **8:**4657-4667.

S4. Ivanic J, Yu X, Wallqvist A, Reifman J: **Influence of protein abundance on high-throughput protein-protein interaction detection.** *PLoS One* 2009, **4:**e5815.

S5. Newman JR, Ghaemmaghami S, Ihmels J, Breslow DK, Noble M, DeRisi JL, Weissman JS: **Single-cell proteomic analysis of S. cerevisiae reveals the architecture of biological noise.** *Nature* 2006, **441:**840-846.

S6. Gasch AP, Spellman PT, Kao CM, Carmel-Harel O, Eisen MB, Storz G, Botstein D, Brown PO: **Genomic expression programs in the response of yeast cells to environmental changes.** *Mol Biol Cell* 2000, **11:**4241-4257.

S7. Lin YS, Hsu WL, Hwang JK, Li WH: **Proportion of solvent-exposed amino acids in a protein and rate of protein evolution.** *Mol Biol Evol* 2007, **24:**1005-1011.

S8. Plotkin JB, Fraser HB: **Assessing the determinants of evolutionary rates in the presence of noise.** *Mol Biol Evol* 2007, **24:**1113-1121.

S9. Peri S, Navarro JD, Amanchy R, Kristiansen TZ, Jonnalagadda CK, Surendranath V, Niranjan V, Muthusamy B, Gandhi TK, Gronborg M, et al: **Development of human protein reference database as an initial platform for approaching systems biology in humans.** *Genome Res* 2003, **13:**2363-2371.

S10. Wheeler DL, Barrett T, Benson DA, Bryant SH, Canese K, Chetvernin V, Church DM, DiCuccio M, Edgar R, Federhen S, et al: **Database resources of the National Center for Biotechnology Information.** *Nucleic Acids Res* 2007, **35:**D5-12.

S11. Tu Z, Wang L, Xu M, Zhou X, Chen T, Sun F: **Further understanding human disease genes by comparing with housekeeping genes and other genes.** *BMC Genomics* 2006, **7:**31.

S12. Shyamsundar R, Kim YH, Higgins JP, Montgomery K, Jorden M, Sethuraman A, van de Rijn M, Botstein D, Brown PO, Pollack JR: **A DNA microarray survey of gene expression in normal human tissues.** *Genome Biol* 2005, **6:**R22.

S13. Whitfield ML, Sherlock G, Saldanha AJ, Murray JI, Ball CA, Alexander KE, Matese JC, Perou CM, Hurt MM, Brown PO, Botstein D: **Identification of genes periodically expressed in the human cell cycle and their expression in tumors.** *Mol Biol Cell* 2002, **13:**1977-2000.

S14. Demeter J, Beauheim C, Gollub J, Hernandez-Boussard T, Jin H, Maier D, Matese JC, Nitzberg M, Wymore F, Zachariah ZK, et al: **The Stanford Microarray Database: implementation of new analysis tools and open source release of software.** *Nucleic Acids Res* 2007, **35:**D766-770.

S15. Kellis M, Patterson N, Endrizzi M, Birren B, Lander ES: **Sequencing and comparison of yeast species to identify genes and regulatory elements.** *Nature* 2003, **423:**241-254.

# Supplementary Figures

## Figure S1 - Relationship of protein evolutionary rate dN with PPID and ePPID.

Scatter plots of protein evolutionary rate dN versus PPID (upper panel) and ePPID (bottom panel) together with the linear regression fit in the three protein interaction datasets: (A) “FYI” (B) “SIN” (C) “Eight-union”.


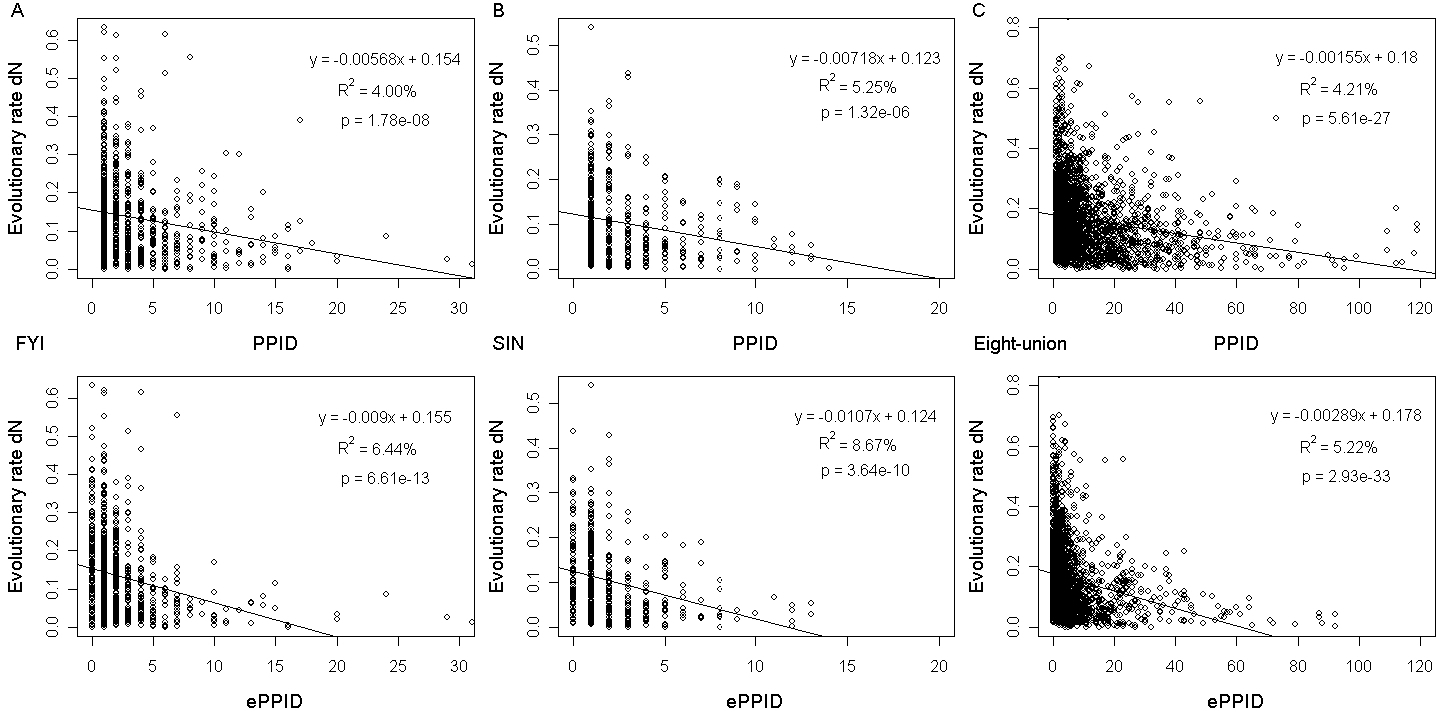


## Figure S2 - Co-expressed proteins evolve more slowly than non-co-expressed proteins.

Protein evolutionary rate dN (y-axis) of non-co-expressed proteins (gray bars) and co-expressed proteins (dark gray bars) is shown as a function of PPID (x-axis). For the purpose of a detailed comparison, non-co-expressed and co-expressed proteins are further grouped into three bins (see Methods for details) according to their PPID values. (A) “FYI” (B) “SIN” (C) “Eight-union”.


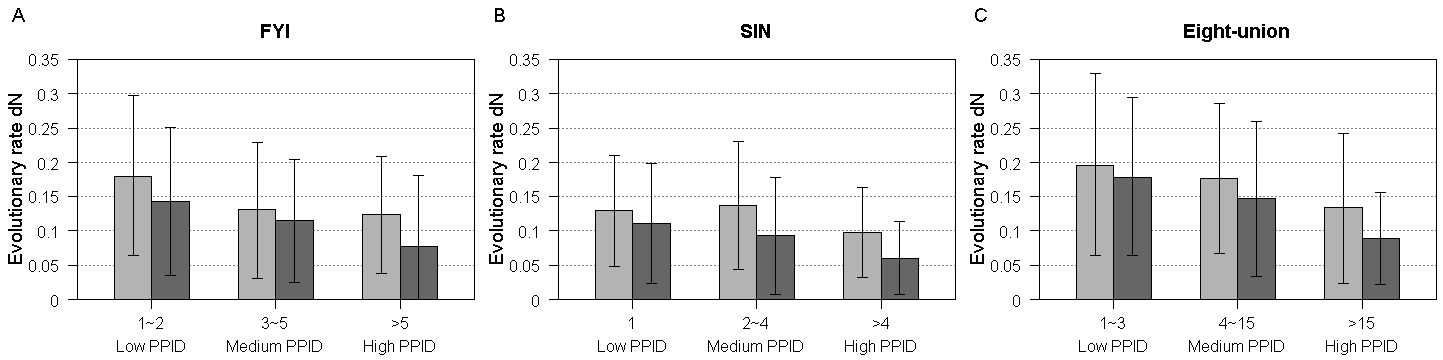


## Figure S3 - Relationship of protein evolutionary rate with PPID and ePPID.

Scatter plots of protein evolutionary rate dN versus PPID (upper panel) and ePPID (bottom panel) together with the linear regression fit in the “Updated-SIN” dataset.


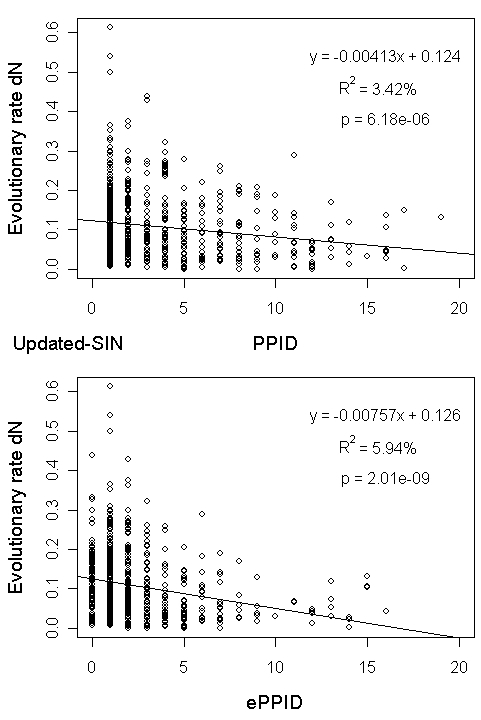


## Figure S4 - Effects of permanent and transient interfaces on protein evolutionary rate.

Mean and standard deviation of protein evolutionary rate dN in the “Updated-SIN” dataset are shown for non-co-expressed proteins with single interface (NC-S, in a total of 30 proteins), non-co-expressed proteins with multiple interfaces (NC-M, in a total of 27 proteins), co-expressed proteins with single interface (C-S, in a total of 20 proteins) and co-expressed proteins with multiple interfaces (C-M, in a total of 88 proteins). C-M evolve significantly more slowly than NC-S (P=2.01e-03), NC-M (P=6.79e-04) and C-S (P=8.55e-03). Other comparisons did not yield significant results (i.e., P>0.05). P is calculated by one-sided Wilcoxon rank sum test.


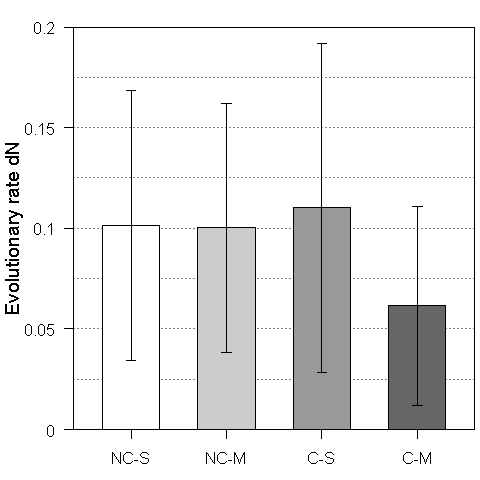


# Supplementary Tables

## Table S1 - The variance of protein evolutionary rate explained by PPID and ePPID in the nine protein interaction datasets.

| **Protein interaction datasets** | *n* | **Percent variance explained in dN** | |
| --- | --- | --- | --- |
|  |  | PPID | ePPID |
| Y2H-union | 1,104 | 0.278 | 1.14 |
| Combined-AP/MS | 922 | 2.80 | 4.63 |
| LC-multiple | 894 | 1.63 | 4.40 |
| Updated-HC | 2,245 | 1.13 | 4.36 |
| DIP-CORE | 1,342 | 1.18 | 2.62 |
| DIP-FULL | 2,572 | 2.37 | 4.44 |
| FYI | 779 | 4.00 | 6.44 |
| SIN | 436 | 5.25 | 8.67 |
| Eight-union | 2,695 | 4.21 | 5.22 |

dN represents protein evolutionary rate measured by non-synonymous substitutions. *n* is the number of proteins for which both PPID and protein evolutionary rate are available.

## Table S2 - The variance of protein evolutionary rate explained by PPID and ePPID in the nine protein interaction datasets by using three other evolutionary rate data [S15].

| **Protein interaction datasets** | **Percent variance explained in dN of *S.cer* vs *S.par*** | | | **Percent variance explained in dN of *S.cer* vs *S.mik*** | | | **Percent variance explained in dN of *S.cer* vs *S.bay*** | | |
| --- | --- | --- | --- | --- | --- | --- | --- | --- | --- |
|  | *n* | PPID | ePPID | *n* | PPID | ePPID | *n* | PPID | ePPID |
| Y2H-union | 1,072 | 0.382 | 0.892 | 807 | 0.595 | 1.31 | 800 | 0.745 | 1.69 |
| Combined-AP/MS | 829 | 1.72 | 3.38 | 643 | 0.995 | 2.67 | 650 | 3.66 | 5.02 |
| LC-multiple | 799 | 1.19 | 3.40 | 618 | 0.787 | 2.47 | 620 | 1.99 | 5.28 |
| Updated-HC | 2,096 | 0.755 | 2.88 | 1,584 | 0.71 | 3.24 | 1,562 | 1.71 | 4.56 |
| DIP-CORE | 1,255 | 2.59 | 3.74 | 982 | 1.46 | 3.40 | 963 | 2.69 | 5.90 |
| DIP-FULL | 2,450 | 2.06 | 3.77 | 1,858 | 2.05 | 3.88 | 1,807 | 3.04 | 4.71 |
| FYI | 714 | 4.41 | 6.66 | 559 | 4.48 | 7.90 | 563 | 4.69 | 7.98 |
| SIN | 440 | 4.56 | 9.07 | 355 | 3.82 | 6.54 | 357 | 5.53 | 10.10 |
| Eight-union | 2,549 | 3.37 | 4.33 | 1,928 | 3.56 | 4.63 | 1,886 | 5.94 | 6.42 |

dN represents protein evolutionary rate measured by non-synonymous substitutions. *n* is the number of proteins for which both PPID and protein evolutionary rate are available.

## Table S3 - Spearman correlation of PPID and ePPID with protein evolutionary rate by using three other evolutionary rate data [S15].

| **Protein interaction datasets** | **dN of *S.cer* vs *S.par*** | | | **dN of *S.cer* vs *S.mik*** | | | **dN of *S.cer* vs *S.bay*** | | |
| --- | --- | --- | --- | --- | --- | --- | --- | --- | --- |
|  | *n* | PPID vs. dN | ePPID vs. dN | *n* | PPID vs. dN | ePPID vs. dN | *n* | PPID vs. dN | ePPID vs. dN |
|  |  | *rho*(*p*) | *rho*(*p*) |  | *rho*(*p*) | *rho*(*p*) |  | *rho*(*p*) | *rho*(*p*) |
| Y2H-union | 1,072 | -0.0431  (1.59e-01) | **-0.0936**  **(2.17e-03)** | 807 | -0.0584  (9.72e-02) | **-0.106**  **(2.52e-03)** | 800 | **-0.0712**  **(4.40e-02)** | **-0.141**  **(6.42e-05)** |
| Combined-AP/MS | 829 | **-0.0966**  **(5.37e-03)** | **-0.196**  **(1.26e-08)** | 643 | **-0.0798**  **(4.32e-02)** | **-0.203**  **(2.05e-07)** | 650 | **-0.167**  **(1.85e-05)** | **-0.258**  **(2.34e-11)** |
| LC-multiple | 799 | **-0.137**  **(9.95e-05)** | **-0.247**  **(1.47e-12)** | 618 | **-0.0980**  **(1.48e-02)** | **-0.229**  **(8.54e-09)** | 620 | **-0.149**  **(1.88e-04)** | **-0.251**  **(2.30e-10)** |
| Updated-HC | 2,096 | **-0.143**  **(4.12e-11)** | **-0.200**  **(2.68e-20)** | 1,584 | **-0.158**  **(2.69e-10)** | **-0.214**  **(8.42e-18)** | 1,562 | **-0.196**  **(6.17e-15)** | **-0.246**  **(6.89e-23)** |
| DIP-CORE | 1,255 | **-0.175**  **(4.41e-10)** | **-0.247**  **(6.80e-19)** | 982 | **-0.144**  **(5.86e-06)** | **-0.254**  **(5.77e-16)** | 963 | **-0.180**  **(1.98e-08)** | **-0.291**  **(3.02e-20)** |
| DIP-FULL | 2,450 | **-0.231**  **(5.06e-31)** | **-0.256**  **(4.40e-38)** | 1,858 | **-0.216**  **(4.41e-21)** | **-0.244**  **(1.24e-26)** | 1,807 | **-0.241**  **(2.66e-25)** | **-0.277**  **(3.01e-33)** |
| FYI | 714 | **-0.239**  **(9.08e-11)** | **-0.294**  **(1.07e-15)** | 559 | **-0.253**  **(1.31e-09)** | **-0.335**  **(4.40e-16)** | 563 | **-0.234**  **(1.94e-08)** | **-0.343**  **(5.47e-17)** |
| SIN | 440 | **-0.232**  **(8.31e-07)** | **-0.337**  **(3.70e-13)** | 355 | **-0.195**  **(2.12e-04)** | **-0.288**  **(3.40e-08)** | 357 | **-0.284**  **(4.89e-08)** | **-0.360**  **(2.18e-12)** |
| Eight-union | 2,549 | **-0.244**  **(8.85e-36)** | **-0.278**  **(1.41e-46)** | 1,928 | **-0.241**  **(6.94e-27)** | **-0.276**  **(4.78e-35)** | 1,886 | **-0.297**  **(8.09e-40)** | **-0.340**  **(4.28e-52)** |

dN represents protein evolutionary rate measured by non-synonymous substitutions. *n* is the number of proteins for which both PPID and protein evolutionary rate are available. *rho* is Spearman rank correlation coefficient, and *p* is the corresponding statistical significance. Bold indicates that *p* is significant at the statistical significance level of 0.05.

## Table S4 - The network properties of the nine protein interaction datasets.

| **Protein interaction datasets** | # proteins | # interactions | # non-co-expressed interactions (percentage) | Average degree | Average clustering coefficient |
| --- | --- | --- | --- | --- | --- |
| Y2H-union | 1,966 | 2,705 | 1,248(46.1%) | 2.75 | 0.097 |
| Combined-AP/MS | 1,622 | 9,070 | 1,304(14.4%) | 11.18 | 0.74 |
| LC-multiple | 1,528 | 2,844 | 866(30.5%) | 3.72 | 0.46 |
| Updated-HC | 4,008 | 9,857 | 3,798(38.5%) | 4.92 | 0.24 |
| DIP-CORE | 2,327 | 4,319 | 1,562(36.2%) | 3.71 | 0.31 |
| DIP-FULL | 4,644 | 17,029 | 7,235(42.5%) | 7.33 | 0.13 |
| FYI | 1,379 | 2,493 | 585(23.5%) | 3.62 | 0.54 |
| SIN | 873 | 1,269 | 270(21.3%) | 2.91 | 0.59 |
| Eight-union | 5,043 | 27,396 | 9,323(34.0%) | 10.86 | 0.20 |

The value in the parentheses shown in column 4 indicates the percentage of non-co-expressed protein interactions in the corresponding dataset.

## Table S5A - The overlap of interactions between the six protein interaction datasets analyzed in the main text.

| Y2H-union(2,705) | 260(9.6%) | 251(9.3%) | 1,110(41.0%) | 796(29.4%) | 1,445(53.4%) |
| --- | --- | --- | --- | --- | --- |
| 260(2.9%) | Combined-AP/MS(9,070) | 1,298(14.3%) | 3,149(34.7%) | 1,059(11.7%) | 2,373(26.2%) |
| 251(8.8%) | 1,298(45.6%) | LC-multiple(2,844) | 2,785(97.9%) | 1,494(52.5%) | 1,813(63.7%) |
| 1,110(11.3%) | 3,149(31.9%) | 2,785(28.3%) | Updated-HC(9,857) | 2,407(24.4%) | 6,995(71.0%) |
| 796(18.4%) | 1,059(24.5%) | 1,494(34.6%) | 2,407(55.7%) | DIP-CORE(4,319) | 4,319(100.0%) |
| 1,445(8.5%) | 2,373(13.9%) | 1,813(10.6%) | 6,995(41.1%) | 4,319(25.4%) | DIP-FULL(17,029) |

Each row corresponds to a dataset and shows a fraction of its interactions contained in other datasets. For example, 260 (9.6%) interactions in the “Y2H-union” dataset are also present in the “Combined-AP/MS” dataset.

## Table S5B - The overlap of interactions between the three protein interaction datasets analyzed in the supplementary text.

| FYI(2,493) | 337(13.5%) | 2493(100.0%) |
| --- | --- | --- |
| 337(26.6%) | SIN(1,269) | 1269(100.0%) |
| 2493(9.1%) | 1269(4.6%) | Eight-union(27,396) |

Each row corresponds to a dataset and shows a fraction of its interactions contained in other datasets. For example, 337 (13.5%) interactions in the “FYI” dataset are also present in the “SIN” dataset.

## Table S5C - The overlap of interactions between the three datasets analyzed in the supplementary text and the six datasets analyzed in the main text.

|  | Y2H-union | Combined-AP/MS | LC-multiple | Updated-HC | DIP-CORE | DIP-FULL |
| --- | --- | --- | --- | --- | --- | --- |
| FYI(2,493) | 257(10.3%) | 1295(51.9%) | 952(38.2%) | 1492(59.8%) | 1261(50.6%) | 1733(69.5%) |
| SIN(1,269) | 93(7.3%) | 413(32.5%) | 259(20.4%) | 497(39.2%) | 405(31.9%) | 607(47.8%) |
| Eight-union(27,396) | 2705(9.9%) | 9070(33.1%) | 2844(10.4%) | 9857(36.0%) | 4319(15.8%) | 17029(62.2%) |

Each row corresponds to a dataset and shows a fraction of its interactions contained in other datasets. For example, 257 (10.3%) interactions in the “FYI” dataset are also present in the “Y2H-union” dataset.

## Table S6A - Principal component regression analysis on six predictor variables and protein evolutionary rate for 1,502 yeast proteins in the “Updated-HC” dataset.

|  | Principal Components | | | | | | |
| --- | --- | --- | --- | --- | --- | --- | --- |
|  | 1 | 2 | 3 | 4 | 5 | 6 | All |
| Percent variance explained in dN | 20.73*** | 13.01*** | 1.32** | 0.19 | 0.17 | 0.15 | 35.57*** |
| Percent contributions |  | | | | | | |
| mRNA abundance | 11.6 | **37.8** | 0.2 | 0.8 | **49.6** | 0.0 |  |
| protein abundance | 14.2 | **33.4** | 1.1 | 0.3 | **49.5** | 1.5 |  |
| gene dispensability | 15.0 | 1.0 | **33.9** | **46.1** | 0.0 | 3.9 |  |
| gene pleiotropy | 12.1 | 6.6 | **61.8** | 19.1 | 0.4 | 0.0 |  |
| ePPID | **28.3** | 6.0 | 2.9 | 7.2 | 0.3 | **55.2** |  |
| betweenness | 18.7 | 15.1 | 0.1 | **26.5** | 0.3 | **39.3** |  |

Note: ^#^P<0.01; ^*^P<10^-3^; ^**^P<10^-6^; ^***^P<10^-9^. Bold indicates that the predictor variable contributes at least 20% to the corresponding principal component.

## Table S6B - Principal component regression analysis on six predictor variables and protein evolutionary rate for 913 yeast proteins in the “DIP-CORE” dataset.

|  | Principal Components | | | | | | |
| --- | --- | --- | --- | --- | --- | --- | --- |
|  | 1 | 2 | 3 | 4 | 5 | 6 | All |
| Percent variance explained in dN | 24.10*** | 9.64*** | 0.88* | 0.54^#^ | 0.30 | 0.20 | 35.66*** |
| Percent contributions |  | | | | | | |
| mRNA abundance | 17.8 | **29.7** | **47.8** | 1.5 | 1.9 | 1.3 |  |
| protein abundance | 19.9 | **26.1** | **38.6** | 14.9 | 0.6 | 0.0 |  |
| gene dispensability | 13.8 | 0.7 | 0.4 | 0.3 | **50.3** | **34.4** |  |
| gene pleiotropy | 9.0 | 10.4 | 0.1 | 0.0 | **46.3** | **34.2** |  |
| ePPID | **25.2** | 7.6 | 7.5 | **45.0** | 0.0 | 14.6 |  |
| betweenness | 14.2 | **25.6** | 5.5 | **38.2** | 0.9 | 15.5 |  |

Note: ^#^P<0.01; ^*^P<10^-3^; ^**^P<10^-6^; ^***^P<10^-9^. Bold indicates that the predictor variable contributes at least 20% to the corresponding principal component.

## Table S6C - Principal component regression analysis on six predictor variables and protein evolutionary rate for 1,698 yeast proteins in the “DIP-FULL” dataset.

|  | Principal Components | | | | | | |
| --- | --- | --- | --- | --- | --- | --- | --- |
|  | 1 | 2 | 3 | 4 | 5 | 6 | All |
| Percent variance explained in dN | 24.82*** | 9.09*** | 1.61*** | 0.24 | 0.14 | 0.01 | 35.91*** |
| Percent contributions |  | | | | | | |
| mRNA abundance | 15.3 | **34.2** | 0.5 | 15.4 | 0.8 | **33.9** |  |
| protein abundance | 17.1 | **30.4** | 1.1 | 8.7 | 0.2 | **42.6** |  |
| gene dispensability | 10.8 | 0.6 | **29.0** | 1.7 | **57.7** | 0.2 |  |
| gene pleiotropy | 10.7 | 9.2 | **63.6** | 0.0 | 16.1 | 0.4 |  |
| ePPID | **26.4** | 8.2 | 4.9 | **40.2** | 7.7 | 12.5 |  |
| betweenness | 19.7 | 17.5 | 0.8 | **34.1** | 17.6 | 10.4 |  |

Note: ^#^P<0.01; ^*^P<10^-3^; ^**^P<10^-6^; ^***^P<10^-9^. Bold indicates that the predictor variable contributes at least 20% to the corresponding principal component.

## Table S6D - Principal component regression analysis on six predictor variables and protein evolutionary rate for 566 yeast proteins in the “FYI” dataset.

|  | Principal Components | | | | | | |
| --- | --- | --- | --- | --- | --- | --- | --- |
|  | 1 | 2 | 3 | 4 | 5 | 6 | All |
| Percent variance explained in dN | 31.81*** | 2.70** | 2.59* | 1.38* | 0.63 | 0.03 | 39.13*** |
| Percent contributions |  | | | | | | |
| mRNA abundance | **32.6** | 11.0 | 0.7 | **54.4** | 0.4 | 1.0 |  |
| protein abundance | **28.7** | 11.8 | 7.3 | **40.2** | 11.7 | 0.3 |  |
| gene dispensability | 7.2 | 11.3 | 5.2 | 0.1 | 0.2 | **75.9** |  |
| gene pleiotropy | 0.2 | **27.4** | **54.5** | 0.5 | 0.3 | 17.1 |  |
| ePPID | **25.3** | 5.8 | 11.4 | 4.5 | **49.5** | 3.5 |  |
| betweenness | 6.1 | **32.8** | **21.0** | 0.2 | **37.9** | 2.1 |  |

Note: ^#^P<0.01; ^*^P<10^-3^; ^**^P<10^-6^; ^***^P<10^-9^. Bold indicates that the predictor variable contributes at least 20% to the corresponding principal component.

## Table S6E - Principal component regression analysis on six predictor variables and protein evolutionary rate for 306 yeast proteins in the “SIN” dataset.

|  | Principal Components | | | | | | |
| --- | --- | --- | --- | --- | --- | --- | --- |
|  | 1 | 2 | 3 | 4 | 5 | 6 | All |
| Percent variance explained in dN | 30.02*** | 1.56^#^ | 0.59 | 0.42 | 0.36 | 0.02 | 32.98*** |
| Percent contributions |  | | | | | | |
| mRNA abundance | **40.9** | 3.7 | **52.5** | 0.4 | 0.5 | 2.0 |  |
| protein abundance | **36.5** | 5.0 | **44.5** | 8.3 | 0.1 | 5.6 |  |
| gene dispensability | 7.7 | **40.0** | 0.0 | 0.2 | **51.8** | 0.3 |  |
| gene pleiotropy | 0.4 | **40.7** | 0.1 | 3.2 | **28.4** | **27.3** |  |
| ePPID | 13.5 | 0.1 | 2.9 | **55.9** | 2.4 | **25.2** |  |
| betweenness | 1.1 | 10.5 | 0.1 | **32.0** | 16.7 | **39.7** |  |

Note: ^#^P<0.01; ^*^P<10^-3^; ^**^P<10^-6^; ^***^P<10^-9^. Bold indicates that the predictor variable contributes at least 20% to the corresponding principal component.

## Table S6F - Principal component regression analysis on six predictor variables and protein evolutionary rate for 1,776 yeast proteins in the “Eight-union” dataset.

|  | Principal Components | | | | | | |
| --- | --- | --- | --- | --- | --- | --- | --- |
|  | 1 | 2 | 3 | 4 | 5 | 6 | All |
| Percent variance explained in dN | 23.63*** | 10.03*** | 2.21*** | 0.19 | 0.09 | 0.03 | 36.18*** |
| Percent contributions |  | | | | | | |
| mRNA abundance | 14.2 | **35.4** | 0.4 | 13.0 | 0.4 | **36.7** |  |
| protein abundance | 15.7 | **31.6** | 1.4 | 4.7 | 0.0 | **46.7** |  |
| gene dispensability | 11.6 | 1.7 | 15.1 | 1.8 | **69.7** | 0.1 |  |
| gene pleiotropy | 11.0 | 11.0 | **72.7** | 0.1 | 4.8 | 0.5 |  |
| ePPID | **27.6** | 4.9 | 7.5 | **45.0** | 6.5 | 8.5 |  |
| betweenness | **20.1** | 15.5 | 3.0 | **35.4** | 18.6 | 7.5 |  |

Note: ^#^P<0.01; ^*^P<10^-3^; ^**^P<10^-6^; ^***^P<10^-9^. Bold indicates that the predictor variable contributes at least 20% to the corresponding principal component.

## Table S7 - The contribution of six predictor variables to the total variance of protein evolutionary rate explained by all six principal components in the nine protein interaction datasets.

| **Percent variance explained in dN** | *n* | mRNA abundance | protein abundance | gene dispensability | gene pleiotropy | ePPID | betweenness | All |
| --- | --- | --- | --- | --- | --- | --- | --- | --- |
| Y2H-union | 752 | 12.618 | 13.018 | 3.824 | 4.123 | 1.596 | 0.562 | 35.74 |
| Combined-AP/MS | 723 | 7.922 | 7.975 | 3.327 | 3.514 | 8.102 | 7.059 | 37.90 |
| LC-multiple | 639 | 8.134 | 7.862 | 4.251 | 2.903 | 8.042 | 6.048 | 37.24 |
| Updated-HC | 1,502 | 7.416 | 7.401 | 3.796 | 4.224 | 6.788 | 5.947 | 35.57 |
| DIP-CORE | 913 | 7.577 | 7.727 | 3.627 | 3.387 | 7.155 | 6.186 | 35.66 |
| DIP-FULL | 1,698 | 6.946 | 7.051 | 3.293 | 4.539 | 7.494 | 6.589 | 35.91 |
| FYI | 566 | 11.425 | 10.262 | 2.754 | 2.217 | 8.872 | 3.602 | 39.13 |
| SIN | 306 | 12.651 | 11.334 | 3.117 | 0.872 | 4.322 | 0.686 | 32.98 |
| Eight-union | 1,776 | 6.939 | 6.925 | 3.305 | 5.296 | 7.267 | 6.448 | 36.18 |

As can be seen, the contribution of ePPID to the total variance is more than all other predictor variables in the “Combined-AP/MS”, “DIP-FULL” and “Eight-union” datasets. In the “LC-multiple” dataset, the ePPID contribution is slightly less than that of mRNA abundance, but more than all other variables. In the “Updated-HC”, “DIP-CORE” and “FYI” datasets, the ePPID contribution is slightly less than mRNA abundance and protein abundance. However, in the “Y2H-union” and “SIN” datasets, the ePPID contribution is less than mRNA abundance and protein abundance.

## Table S8A - Statistical significance of the differences in protein evolutionary rate between non-co-expressed and co-expressed proteins in the high-PPID bin.

| **Protein interaction datasets** | CPHB | NCPHB | P_wilcoxon_ |
| --- | --- | --- | --- |
| Y2H-union | 0.131 | 0.154 | 9.15e-02 |
| Combined-AP/MS | 0.0938 | 0.169 | **4.48e-05** |
| LC-multiple | 0.103 | 0.124 | **3.04e-02** |
| Updated-HC | 0.0996 | 0.157 | **1.61e-10** |
| DIP-CORE | 0.120 | 0.143 | **8.65e-03** |
| DIP-FULL | 0.0845 | 0.132 | **1.74e-07** |
| FYI | 0.0776 | 0.124 | **2.13e-05** |
| SIN | 0.0615 | 0.101 | **5.33e-03** |
| Eight-union | 0.0881 | 0.133 | **4.91e-07** |

The mean evolutionary rate dN are calculated for non-co-expressed and co-expressed proteins in the high-PPID bin. P_wilcoxon_ is the statistical significance indicating whether co-expressed proteins evolve more slowly than non-co-expressed proteins in the high-PPID bin, as calculated by one-sided Wilcoxon rank sum test. Bold indicates that the P_wilcoxon_ is significant at the statistical significance level of 0.05. CPHB represents co-expressed proteins in the high-PPID bin, and NCPHB represents non-co-expressed proteins in the high-PPID bin.

## Table S8B - Statistical significance of the differences in protein evolutionary rate between non-co-expressed and co-expressed proteins in the medium-PPID bin.

| **Protein interaction datasets** | CPMB | NCPMB | P_wilcoxon_ |
| --- | --- | --- | --- |
| Y2H-union | 0.159 | 0.172 | 1.05e-01 |
| Combined-AP/MS | 0.102 | 0.170 | **9.42e-10** |
| LC-multiple | 0.125 | 0.179 | **6.27e-04** |
| Updated-HC | 0.152 | 0.180 | **2.19e-05** |
| DIP-CORE | 0.130 | 0.170 | **4.74e-05** |
| DIP-FULL | 0.147 | 0.174 | **1.79e-05** |
| FYI | 0.115 | 0.130 | 1.59e-01 |
| SIN | 0.0937 | 0.137 | **2.08e-03** |
| Eight-union | 0.147 | 0.177 | **5.96e-08** |

The mean evolutionary rate dN are calculated for non-co-expressed and co-expressed proteins in the medium-PPID bin. P_wilcoxon_ is the statistical significance indicating whether co-expressed proteins evolve more slowly than non-co-expressed proteins in the medium-PPID bin, as calculated by one-sided Wilcoxon rank sum test. Bold indicates that the P_wilcoxon_ is significant at the statistical significance level of 0.05. CPMB represents co-expressed proteins in the medium-PPID bin, and NCPMB represents non-co-expressed proteins in the medium-PPID bin.

## Table S8C - Statistical significance of the differences in protein evolutionary rate between non-co-expressed and co-expressed proteins in the low-PPID bin.

| **Protein interaction datasets** | CPLB | NCPLB | P_wilcoxon_ |
| --- | --- | --- | --- |
| Y2H-union | 0.143 | 0.186 | **7.12e-05** |
| Combined-AP/MS | 0.138 | 0.189 | **3.11e-05** |
| LC-multiple | 0.144 | 0.189 | **6.14e-05** |
| Updated-HC | 0.173 | 0.191 | **7.57e-03** |
| DIP-CORE | 0.151 | 0.193 | **1.02e-05** |
| DIP-FULL | 0.177 | 0.191 | **4.16e-02** |
| FYI | 0.143 | 0.180 | **2.79e-04** |
| SIN | 0.111 | 0.129 | **4.53e-02** |
| Eight-union | 0.179 | 0.197 | **3.77e-02** |

The mean evolutionary rate dN are calculated for non-co-expressed and co-expressed proteins in the low-PPID bin. P_wilcoxon_ is the statistical significance indicating whether co-expressed proteins evolve more slowly than non-co-expressed proteins in the low-PPID bin, as calculated by one-sided Wilcoxon rank sum test. Bold indicates that the P_wilcoxon_ is significant at the statistical significance level of 0.05. CPLB represents co-expressed proteins in the low-PPID bin, and NCPLB represents non-co-expressed proteins in the low-PPID bin.

## Table S9 - Correspondence of singlish-interface and multi-interface hubs to non-co-expressed and co-expressed hubs in the “SIN” dataset.

| **Hub class** | Non-co-expressed hubs (47) | Co-expressed hubs (120) |
| --- | --- | --- |
| Singlish-interface hubs (36) | 18 | 18 |
| Multi-interface hubs (131) | 29 | 102 |

The number in parentheses indicates the number of the corresponding hub class.

## Table S10 - Spearman correlation of PPID, ePPID and betweenness with protein evolutionary rate in the “FYI”, “SIN” and “Eight-union” datasets.

| **Protein interaction datasets** | *n* | PPID vs. dN | ePPID vs. dN | betweenness vs. dN |
| --- | --- | --- | --- | --- |
|  |  | *rho*(*p*) | *rho*(*p*) | *rho*(*p*) |
| FYI | 779 | **-0.239(1.47e-11)** | **-0.314(2.44e-19)** | **-0.100(5.12e-03)** |
| SIN | 436 | **-0.234(8.10e-07)** | **-0.339(3.30e-13)** | **-0.107(2.53e-02)** |
| Eight-union | 2,695 | **-0.258(3.86e-42)** | **-0.294(5.52e-55)** | **-0.204(9.88e-27)** |

dN represents protein evolutionary rate measured by non-synonymous substitutions. *n* is the number of proteins for which both PPID and protein evolutionary rate are available. *rho* is Spearman rank correlation coefficient, and *p* is the corresponding statistical significance. Bold indicates that *p* is significant at the statistical significance level of 0.05.

## Table S11 - The variance of protein evolutionary rate explained by PPID and ePPID when controlling for protein abundance in the “FYI”, “SIN” and “Eight-union” datasets.

| **Protein interaction datasets** | *n* | **Percent variance explained in dN** | | | |
| --- | --- | --- | --- | --- | --- |
|  |  | PPID(*p*) | PPID control for Log(abundance)(*p*) | ePPID(*p*) | ePPID control for Log(abundance)(*p*) |
| FYI | 606 | **2.73(4.37e-05)** | **1.97(5.36e-04)** | **5.56(4.23e-09)** | **2.44(1.13e-04)** |
| SIN | 320 | **3.52(7.39e-04)** | **5.28(3.33e-05)** | **6.58(3.33e-06)** | **5.71(1.56e-05)** |
| Eight-union | 1,874 | **3.21(5.58e-15)** | **1.32(6.14e-07)** | **4.53(1.20e-20)** | **1.19(2.11e-06)** |

dN represents protein evolutionary rate measured by non-synonymous substitutions. *n* is the number of proteins for which PPID, protein evolutionary rate and abundance data are all available. *p* in column 3 and 5 is the statistical significance of the linear regression of protein evolutionary rate against PPID and ePPID, respectively. *p* in column 4 and 6 is the statistical significance of the linear regression of protein evolutionary rate against PPID and ePPID when controlling for protein abundance, respectively. Bold indicates that *p* is significant at the statistical significance level of 0.05.

## Table S12 - Spearman correlation and partial Spearman correlation of PPID and ePPID with protein evolutionary rate in the “FYI”, “SIN” and “Eight-union” datasets.

| **Protein interaction datasets** | *N* | PPID vs. dN | PPID vs. dN control for abundance | ePPID vs. dN | ePPID vs. dN control for abundance |
| --- | --- | --- | --- | --- | --- |
|  |  | *rho*(*p*) | *rho*(*p*) | *rho*(*p*) | *rho*(*p*) |
| FYI | 606 | **-0.206(3.25e-07)** | **-0.164(4.35e-05)** | **-0.293(1.72e-13)** | **-0.194(1.23e-06)** |
| SIN | 320 | **-0.146(8.72e-03)** | **-0.163(3.35e-03)** | **-0.253(4.61e-06)** | **-0.189(6.12e-04)** |
| Eight-union | 1,874 | **-0.218(1.12e-21)** | **-0.146(1.92e-10)** | **-0.252(1.40e-28)** | **-0.146(1.67e-10)** |

dN represents protein evolutionary rate measured by non-synonymous substitutions. *n* is the number of proteins for which PPID, protein evolutionary rate and abundance data are all available. *rho* is Spearman rank correlation coefficient, and *p* is the corresponding statistical significance. Bold indicates that *p* is significant at the statistical significance level of 0.05.

## Table S13 - Spearman correlation of PPID and ePPID with protein abundance.

| **Protein interaction datasets** | *n* | PPID vs. Abundance | ePPID vs. Abundance |
| --- | --- | --- | --- |
|  |  | *rho*(*p*) | *rho*(*p*) |
| Y2H-union | 793 | 0.00385(9.14e-01) | **0.0731(3.96e-02)** |
| Combined-AP/MS | 763 | **0.160(8.67e-06)** | **0.296(6.31e-17)** |
| LC-multiple | 680 | 0.0651(8.96e-02) | **0.219(7.91e-09)** |
| Updated-HC | 1,587 | **0.111(1.01e-05)** | **0.216(2.84e-18)** |
| DIP-CORE | 968 | **0.0994(1.95e-03)** | **0.245(1.15e-14)** |
| DIP-FULL | 1,792 | **0.173(1.60e-13)** | **0.251(4.12e-27)** |
| FYI | 606 | **0.130(1.30e-03)** | **0.267(2.29e-11)** |
| SIN | 320 | 0.0138(8.06e-01) | **0.182(1.09e-03)** |
| Eight-union | 1,874 | **0.196(1.25e-17)** | **0.271(6.32e-33)** |

*n* is the number of proteins for which PPID, protein evolutionary rate and abundance data are all available. *rho* is Spearman rank correlation coefficient, and *p* is the corresponding statistical significance. Bold indicates that *p* is significant at the statistical significance level of 0.05.

## Table S14 - Some protein examples which tend to have high ePPID but low APCC.

| **Protein** | **PPID** | **ePPID** | **ePPID/PPID** | **APCC** | **Protein’s interaction partners** | **PCC between a protein and its interaction partners** |
| --- | --- | --- | --- | --- | --- | --- |
| YBR217W | 10 | 6 | 0.6 | 0.191 | YLR031W,YMR159C,YHR171W,YLL042C,  YNR007C,YLR423C,YPL149W,YDR465C,  YML055W,YMR047C | 0.592, 0.571, 0.547, 0.482,  0.455, 0.386, 0866, -0.299,  -0.371, -0.542 |
| YLR449W | 7 | 4 | 0.571 | 0.217 | YDR496C,YNL061W,YMR049C,YPL093W,  YLR453C,YIL050W,YJL023C | 0.867, 0.834, 0.792, 0.634,  -0.232, -0.683, -0.691 |
| YFR049W | 10 | 6 | 0.6 | 0.259 | YDR148C,YOL082W,YOL083W,YFL018C,  YOR049C,YMR147W,YOL062C,YPL049C,  YPL255W,YOR047C | 0.820, 0.762, 0.682, 0.519,  0.479, 0.407, -0.090, -0.198,  -0.326, -0.465 |
| YDR507C | 8 | 5 | 0.625 | 0.264 | YLR314C,YCR002C,YDL225W,YJR076C,  YHR107C,YBR160W,YKR048C,YMR139W | 0.566, 0.546, 0.539, 0.482,  0.418, 0.280, -0.106, -0.611 |
| YCR052W | 16 | 9 | 0.563 | 0.283 | YMR033W,YKR008W,YOR232W,YMR091C,  YLR357W,YDR303C,YLR321C,YLR033W,  YGR056W,YPR034W,YGR275W,YFR037C,  YIL126W,YIL084C,YML127W,YNL202W | 0.603, 0.544, 0.519, 0.513,  0.510, 0.478, 0.459, 0.426,  0.413, 0.337, 0.222, 0.182,  0.116, -0.093, -0.197, -0.501 |
| YDR303C | 15 | 9 | 0.6 | 0.303 | YMR033W,YMR091C,YKR008W,YGR056W,  YCR052W,YBR009C,YLR357W,YLR033W,  YLR321C,YPR034W,YIL126W,YML127W,  YGR275W,YFR037C,YHR056C | 0.706, 0.670, 0.518, 0.486,  0.478, 0.460, 0.453, 0.435,  0.406, 0.334, 0.090, -0.047,  -0.074, -0.123, -0.249 |
| YLR357W | 16 | 9 | 0.563 | 0.316 | YMR033W,YKR008W,YMR091C,YGR056W,  YCR052W,YLR033W,YLR321C,YDR303C,  YER148W,YGR275W,YPR034W,YIL126W,  YFR037C,YMR224C,YML127W,YHR056C | 0.593, 0.572, 0.527, 0.514,  0.510, 0.473, 0.457, 0.453,  0.412, 0.202, 0.172, 0.169,  0.125, 0.110, -0.118, -0.123 |
| YBR060C | 11 | 7 | 0.636 | 0.318 | YHR118C,YNL261W,YGR103W,YML065W,  YBR160W,YLL004W,YDR052C,YPR162C,  YLR117C,YGR024C,YAL051W | 0.526, 0.526, 0.495, 0.488,  0.429, 0.393, 0.392, 0.377,  0.083, -0.018, -0.191 |
| YHR187W | 8 | 5 | 0.625 | 0.321 | YPL101W,YPL086C,YGR200C,YMR312W,  YKL110C,YLR384C,YMR047C,YBL021C | 0.598, 0.528, 0.479, 0.453,  0.447, 0.164, 0.089, -0.187 |
| YGL233W | 7 | 4 | 0.571 | 0.330 | YLR166C,YBR102C,YPR055W,YIL068C,  YFL005W,YDR166C,YBR200W | 0.724, 0.630, 0.617, 0.397,  0.033, -0.020, -0.069 |

## Table S15 - Spearman correlation of ePPID_avg_ and ePPID_sec_ with protein evolutionary rate in the nine protein interaction datasets.

| **Protein interaction datasets** | *n* | PPID vs. dN | ePPID vs. dN | ePPID_avg_ vs. dN | ePPID_sec_ vs. dN |
| --- | --- | --- | --- | --- | --- |
|  |  | *rho*(*p*) | *rho*(*p*) | *rho*(*p*) | *rho*(*p*) |
| Y2H-union | 1,104 | -0.0487(1.06e-01) | **-0.142(2.25e-06)** | **-0.197(4.00e-11)** | **-0.168(2.02e-08)** |
| Combined-AP/MS | 922 | **-0.158(1.46e-06)** | **-0.251(1.03e-14)** | **-0.311(4.03e-22)** | **-0.275(1.99e-17)** |
| LC-multiple | 894 | **-0.172(2.46e-07)** | **-0.267(4.72e-16)** | **-0.339(2.02e-25)** | **-0.296(1.54e-19)** |
| Updated-HC | 2,245 | **-0.183(2.62e-18)** | **-0.242(2.58e-31)** | **-0.266(8.49e-38)** | **-0.248(1.06e-32)** |
| DIP-CORE | 1,342 | **-0.152(2.33e-08)** | **-0.254(3.69e-21)** | **-0.330(1.66e-35)** | **-0.300(2.79e-29)** |
| DIP-FULL | 2,572 | **-0.233(4.56e-33)** | **-0.271(1.40e-44)** | **-0.294(1.47e-52)** | **-0.284(6.30e-49)** |
| FYI | 779 | **-0.239(1.47e-11)** | **-0.314(2.44e-19)** | **-0.409(1.05e-32)** | **-0.362(1.42e-25)** |
| SIN | 436 | **-0.234(8.10e-07)** | **-0.339(3.30e-13)** | **-0.460(3.62e-24)** | **-0.400(3.53e-18)** |
| Eight-union | 2,695 | **-0.258(3.86e-42)** | **-0.294(5.52e-55)** | **-0.315(5.07e-63)** | **-0.298(1.65e-56)** |

dN represents protein evolutionary rate measured by non-synonymous substitutions. *n* is the number of proteins for which both PPID and protein evolutionary rate are available. *rho* is Spearman rank correlation coefficient, and *p* is the corresponding statistical significance. Bold indicates that *p* is significant at the statistical significance level of 0.05.

## Table S16 - The variance of protein evolutionary rate explained by ePPID_avg_ and ePPID_sec_ in the nine protein interaction datasets.

| **Protein interaction datasets** | *n* | **Percent variance explained in dN** | | | |
| --- | --- | --- | --- | --- | --- |
|  |  | PPID | ePPID | ePPID_avg_ | ePPID_sec_ |
| Y2H-union | 1,104 | 0.278 | 1.14 | 1.62 | 1.19 |
| Combined-AP/MS | 922 | 2.80 | 4.63 | 4.91 | 5.07 |
| LC-multiple | 894 | 1.63 | 4.40 | 5.92 | 5.60 |
| Updated-HC | 2,245 | 1.13 | 4.36 | 4.68 | 4.61 |
| DIP-CORE | 1,342 | 1.18 | 2.62 | 4.57 | 3.97 |
| DIP-FULL | 2,572 | 2.37 | 4.44 | 4.57 | 4.94 |
| FYI | 779 | 4.00 | 6.44 | 6.67 | 7.57 |
| SIN | 436 | 5.25 | 8.67 | 11.30 | 10.01 |
| Eight-union | 2,695 | 4.21 | 5.22 | 4.45 | 5.06 |

dN represents protein evolutionary rate measured by non-synonymous substitutions. *n* is the number of proteins for which both PPID and protein evolutionary rate are available.

## Table S17 - Spearman correlation between nePPID and protein evolutionary rate.

| **Protein interaction datasets** | *n* | nePPID vs. dN |
| --- | --- | --- |
|  |  | *rho*(*p*) |
| Y2H-union | 1,104 | 0.0543(7.13e-02) |
| Combined-AP/MS | 922 | **0.169(2.30e-07)** |
| LC-multiple | 894 | **0.105(1.75e-03)** |
| Updated-HC | 2,245 | 0.00761(7.18e-01) |
| DIP-CORE | 1,342 | **0.0644(1.82e-02)** |
| DIP-FULL | 2,572 | **-0.114(6.37e-09)** |
| FYI | 779 | **0.113(1.63e-03)** |
| SIN | 436 | **0.167(4.47e-04)** |
| Eight-union | 2,695 | **-0.112(5.00e-09)** |

dN represents protein evolutionary rate measured by non-synonymous substitutions. *n* is the number of proteins for which both nePPID and protein evolutionary rate are available. *rho* is Spearman rank correlation coefficient, and *p* is the corresponding statistical significance. Bold indicates that *p* is significant at the statistical significance level of 0.05.

## Table S18A - Principal component regression analysis on six predictor variables and protein evolutionary rate for 752 yeast proteins in the “Y2H-union” dataset.

|  | Principal Components | | | | | | |
| --- | --- | --- | --- | --- | --- | --- | --- |
|  | 1 | 2 | 3 | 4 | 5 | 6 | All |
| Percent variance explained in dN | 40.82*** | 0.31 | 0.12 | 0.03 | 0.01 | 0.01 | 41.29*** |
| Percent contributions |  | | | | | | |
| mRNA abundance | **44.4** | **47.2** | 0.5 | 2.6 | 3.6 | 1.7 |  |
| CAI | **39.4** | **47.0** | 1.8 | 0.6 | 10.9 | 0.3 |  |
| gene dispensability | 5.2 | 2.8 | 0.3 | 0.1 | **47.6** | **44.1** |  |
| gene pleiotropy | 8.6 | 0.0 | 0.3 | 0.4 | **37.3** | **53.4** |  |
| ePPID | 2.3 | 0.6 | **47.3** | **49.4** | 0.0 | 0.3 |  |
| betweenness | 0.1 | 2.3 | **49.7** | **46.8** | 0.7 | 0.3 |  |

Note: ^#^P<0.01; ^*^P<10^-3^; ^**^P<10^-6^; ^***^P<10^-9^. Bold indicates that the predictor variable contributes at least 20% to the corresponding principal component.

## Table S18B - Principal component regression analysis on six predictor variables and protein evolutionary rate for 723 yeast proteins in the “Combined-AP/MS” dataset.

|  | Principal Components | | | | | | |
| --- | --- | --- | --- | --- | --- | --- | --- |
|  | 1 | 2 | 3 | 4 | 5 | 6 | All |
| Percent variance explained in dN | 30.00*** | 8.81*** | 1.66* | 0.45 | 0.01 | 0.00 | 40.93*** |
| Percent contributions |  | | | | | | |
| mRNA abundance | **26.4** | 6.1 | 12.5 | 3.9 | 2.9 | **48.2** |  |
| CAI | **22.5** | 12.0 | 18.2 | 0.2 | 4.2 | **42.9** |  |
| gene dispensability | 6.4 | 0.4 | **27.4** | **58.6** | 6.9 | 0.3 |  |
| gene pleiotropy | 0.0 | **63.2** | **28.3** | 6.0 | 2.4 | 0.0 |  |
| ePPID | **24.1** | 12.8 | 6.8 | 2.2 | **48.7** | 5.4 |  |
| betweenness | **20.6** | 5.4 | 6.9 | **29.0** | **34.8** | 3.2 |  |

Note: ^#^P<0.01; ^*^P<10^-3^; ^**^P<10^-6^; ^***^P<10^-9^. Bold indicates that the predictor variable contributes at least 20% to the corresponding principal component.

## Table S18C - Principal component regression analysis on six predictor variables and protein evolutionary rate for 639 yeast proteins in the “LC-multiple” dataset.

|  | Principal Components | | | | | | |
| --- | --- | --- | --- | --- | --- | --- | --- |
|  | 1 | 2 | 3 | 4 | 5 | 6 | All |
| Percent variance explained in dN | 28.74*** | 7.64*** | 1.32* | 0.46 | 0.34 | 0.24 | 38.73*** |
| Percent contributions |  | | | | | | |
| mRNA abundance | **22.2** | **22.6** | 1.9 | 2.9 | **48.7** | 1.8 |  |
| CAI | **20.3** | **22.8** | 0.3 | 6.9 | **42.7** | 7.0 |  |
| gene dispensability | 11.1 | 0.2 | 15.4 | **70.1** | 0.1 | 3.1 |  |
| gene pleiotropy | 2.5 | **26.3** | **50.7** | 19.6 | 0.0 | 0.8 |  |
| ePPID | **26.0** | 10.4 | 5.2 | 0.1 | 4.5 | **53.7** |  |
| betweenness | 17.9 | 17.7 | **26.5** | 0.4 | 3.9 | **33.6** |  |

Note: ^#^P<0.01; ^*^P<10^-3^; ^**^P<10^-6^; ^***^P<10^-9^. Bold indicates that the predictor variable contributes at least 20% to the corresponding principal component.

## Table S18D - Principal component regression analysis on six predictor variables and protein evolutionary rate for 1,502 yeast proteins in the “Updated-HC” dataset.

|  | Principal Components | | | | | | |
| --- | --- | --- | --- | --- | --- | --- | --- |
|  | 1 | 2 | 3 | 4 | 5 | 6 | All |
| Percent variance explained in dN | 20.97*** | 17.00*** | 1.10** | 0.23 | 0.21 | 0.15 | 39.67*** |
| Percent contributions |  | | | | | | |
| mRNA abundance | 11.4 | **38.0** | 0.1 | 0.0 | **47.4** | 3.0 |  |
| CAI | 9.7 | **39.6** | 1.2 | 0.7 | **41.7** | 7.1 |  |
| gene dispensability | 15.0 | 1.7 | 19.2 | **58.4** | 0.0 | 5.6 |  |
| gene pleiotropy | 13.3 | 4.7 | **73.7** | 7.9 | 0.3 | 0.1 |  |
| ePPID | **29.6** | 5.2 | 4.8 | 5.4 | 6.4 | **48.5** |  |
| betweenness | **21.0** | 10.7 | 1.0 | **27.6** | 4.1 | **35.7** |  |

Note: ^#^P<0.01; ^*^P<10^-3^; ^**^P<10^-6^; ^***^P<10^-9^. Bold indicates that the predictor variable contributes at least 20% to the corresponding principal component.

## Table S18E - Principal component regression analysis on six predictor variables and protein evolutionary rate for 913 yeast proteins in the “DIP-CORE” dataset.

|  | Principal Components | | | | | | |
| --- | --- | --- | --- | --- | --- | --- | --- |
|  | 1 | 2 | 3 | 4 | 5 | 6 | All |
| Percent variance explained in dN | 26.10*** | 11.51*** | 1.35* | 0.49^#^ | 0.10 | 0.00 | 39.56*** |
| Percent contributions |  | | | | | | |
| mRNA abundance | 19.7 | **28.0** | 0.4 | 0.2 | 0.1 | **51.6** |  |
| CAI | 15.7 | **32.3** | 9.5 | 0.2 | 1.1 | **41.3** |  |
| gene dispensability | 12.9 | 2.1 | 0.0 | **41.6** | **43.1** | 0.2 |  |
| gene pleiotropy | 9.6 | 9.0 | 0.1 | **26.5** | **54.8** | 0.1 |  |
| ePPID | **27.1** | 6.5 | **47.6** | 13.9 | 0.1 | 4.7 |  |
| betweenness | 15.0 | **22.1** | **42.3** | 17.6 | 0.7 | 2.2 |  |

Note: ^#^P<0.01; ^*^P<10^-3^; ^**^P<10^-6^; ^***^P<10^-9^. Bold indicates that the predictor variable contributes at least 20% to the corresponding principal component.

## Table S18F - Principal component regression analysis on six predictor variables and protein evolutionary rate for 1,698 yeast proteins in the “DIP-FULL” dataset.

|  | Principal Components | | | | | | |
| --- | --- | --- | --- | --- | --- | --- | --- |
|  | 1 | 2 | 3 | 4 | 5 | 6 | All |
| Percent variance explained in dN | 26.91*** | 10.74*** | 1.03** | 0.40* | 0.24^#^ | 0.11 | 39.44*** |
| Percent contributions |  | | | | | | |
| mRNA abundance | 16.9 | **32.5** | 0.1 | 0.0 | 10.1 | **40.4** |  |
| CAI | 15.0 | **34.6** | 1.4 | 0.3 | 15.5 | **33.3** |  |
| gene dispensability | 9.8 | 2.4 | **22.8** | **61.8** | 3.1 | 0.0 |  |
| gene pleiotropy | 11.2 | 7.6 | **69.3** | 11.3 | 0.6 | 0.1 |  |
| ePPID | **26.6** | 8.4 | 5.7 | 7.3 | **36.9** | 15.1 |  |
| betweenness | **20.5** | 14.5 | 0.7 | 19.5 | **33.8** | 11.1 |  |

Note: ^#^P<0.01; ^*^P<10^-3^; ^**^P<10^-6^; ^***^P<10^-9^. Bold indicates that the predictor variable contributes at least 20% to the corresponding principal component.

## Table S18G - Principal component regression analysis on six predictor variables and protein evolutionary rate for 566 yeast proteins in the “FYI” dataset.

|  | Principal Components | | | | | | |
| --- | --- | --- | --- | --- | --- | --- | --- |
|  | 1 | 2 | 3 | 4 | 5 | 6 | All |
| Percent variance explained in dN | 35.25*** | 3.46** | 1.80* | 1.11^#^ | 0.12 | 0.09 | 41.84*** |
| Percent contributions |  | | | | | | |
| mRNA abundance | **36.1** | 1.3 | 7.6 | 0.5 | **54.3** | 0.2 |  |
| CAI | **30.0** | 4.1 | 11.7 | 10.3 | **41.6** | 2.2 |  |
| gene dispensability | 4.6 | 18.8 | 15.0 | 0.1 | 0.3 | **61.3** |  |
| gene pleiotropy | 0.0 | **39.5** | **25.0** | 0.2 | 0.3 | **35.0** |  |
| ePPID | **24.4** | 11.5 | 8.1 | **52.3** | 3.3 | 0.5 |  |
| betweenness | 4.9 | **24.9** | **32.6** | **36.6** | 0.2 | 0.8 |  |

Note: ^#^P<0.01; ^*^P<10^-3^; ^**^P<10^-6^; ^***^P<10^-9^. Bold indicates that the predictor variable contributes at least 20% to the corresponding principal component.

## Table S18H - Principal component regression analysis on six predictor variables and protein evolutionary rate for 306 yeast proteins in the “SIN” dataset.

|  | Principal Components | | | | | | |
| --- | --- | --- | --- | --- | --- | --- | --- |
|  | 1 | 2 | 3 | 4 | 5 | 6 | All |
| Percent variance explained in dN | 31.41*** | 1.50^#^ | 0.72 | 0.63 | 0.02 | 0.0 | 34.29*** |
| Percent contributions |  | | | | | | |
| mRNA abundance | **44.2** | 1.3 | 0.0 | 0.6 | **52.5** | 1.3 |  |
| CAI | **37.5** | 5.4 | 1.3 | 5.6 | **43.8** | 6.4 |  |
| gene dispensability | 3.7 | 17.9 | **70.7** | 3.0 | 1.5 | 3.2 |  |
| gene pleiotropy | 0.8 | **62.3** | 7.5 | 2.7 | 0.0 | **26.7** |  |
| ePPID | 13.0 | 1.0 | 1.2 | **56.4** | 2.2 | **26.3** |  |
| betweenness | 0.9 | 12.1 | 19.2 | **31.7** | 0.0 | **36.0** |  |

Note: ^#^P<0.01; ^*^P<10^-3^; ^**^P<10^-6^; ^***^P<10^-9^. Bold indicates that the predictor variable contributes at least 20% to the corresponding principal component.

## Table S18I - Principal component regression analysis on six predictor variables and protein evolutionary rate for 1,776 yeast proteins in the “Eight-union” dataset.

|  | Principal Components | | | | | | |
| --- | --- | --- | --- | --- | --- | --- | --- |
|  | 1 | 2 | 3 | 4 | 5 | 6 | All |
| Percent variance explained in dN | 25.47*** | 11.65*** | 1.79*** | 0.31^#^ | 0.27^#^ | 0.05 | 39.54*** |
| Percent contributions |  | | | | | | |
| mRNA abundance | 15.6 | **33.5** | 0.0 | 0.2 | 12.8 | **37.9** |  |
| CAI | 14.0 | **35.2** | 1.5 | 0.2 | **22.1** | **27.0** |  |
| gene dispensability | 10.5 | 4.2 | 7.5 | **74.6** | 3.2 | 0.0 |  |
| gene pleiotropy | 11.3 | 8.9 | **77.9** | 1.0 | 0.8 | 0.0 |  |
| ePPID | **27.9** | 5.1 | 9.2 | 4.7 | **32.9** | **20.2** |  |
| betweenness | **20.7** | 13.1 | 3.9 | 19.3 | **28.1** | 14.9 |  |

Note: ^#^P<0.01; ^*^P<10^-3^; ^**^P<10^-6^; ^***^P<10^-9^. Bold indicates that the predictor variable contributes at least 20% to the corresponding principal component.

## Table S19 - The contribution of six predictor variables to the total variance of protein evolutionary rate explained by all six principal components in the nine protein interaction datasets.

| **Percent variance explained in dN** | *n* | mRNA abundance | CAI | gene dispensability | gene pleiotropy | ePPID | betweenness | All |
| --- | --- | --- | --- | --- | --- | --- | --- | --- |
| Y2H-union | 752 | 18.273 | 16.238 | 2.125 | 3.523 | 1.016 | 0.121 | 41.29 |
| Combined-AP/MS | 723 | 8.672 | 8.111 | 2.683 | 6.070 | 8.480 | 6.909 | 40.93 |
| LC-multiple | 639 | 8.299 | 7.766 | 3.750 | 3.486 | 8.491 | 6.938 | 38.73 |
| Updated-HC | 1,502 | 8.968 | 8.870 | 3.796 | 4.422 | 7.253 | 6.360 | 39.67 |
| DIP-CORE | 913 | 8.369 | 7.944 | 3.859 | 3.718 | 8.538 | 7.132 | 39.56 |
| DIP-FULL | 1,698 | 8.113 | 7.854 | 3.403 | 4.578 | 8.248 | 7.240 | 39.44 |
| FYI | 566 | 12.991 | 11.108 | 2.597 | 1.853 | 9.723 | 3.566 | 41.84 |
| SIN | 306 | 13.905 | 11.904 | 1.957 | 1.246 | 4.461 | 0.812 | 34.29 |
| Eight-union | 1,776 | 7.929 | 7.764 | 3.541 | 5.318 | 7.980 | 7.012 | 39.54 |

As can be seen, the contribution of ePPID to the total variance is more than all other predictor variables in the “LC-multiple”, “DIP-CORE”, “DIP-FULL” and “Eight-union” datasets. In the “Combined-AP/MS” dataset, the ePPID contribution is slightly less than that of mRNA abundance, but more than all other variables. In the “Updated-HC” and “FYI” datasets, the ePPID contribution is slightly less than mRNA abundance and CAI. However, in the “Y2H-union” and “SIN” datasets, the ePPID contribution is less than mRNA abundance and CAI.

## Table S20A - Principal component regression analysis on six predictor variables and protein evolutionary rate for 752 yeast proteins in the “Y2H-union” dataset.

|  | Principal Components | | | | | | |
| --- | --- | --- | --- | --- | --- | --- | --- |
|  | 1 | 2 | 3 | 4 | 5 | 6 | All |
| Percent variance explained in dN | 37.18*** | 1.50* | 0.03 | 0.02 | 0.01 | 0.01 | 38.74*** |
| Percent contributions |  | | | | | | |
| protein abundance | **44.2** | **48.0** | 3.8 | 1.5 | 0.2 | 2.4 |  |
| CAI | **37.9** | **46.4** | 14.4 | 0.1 | 0.8 | 0.4 |  |
| gene dispensability | 6.5 | 3.2 | **46.9** | **43.1** | 0.1 | 0.1 |  |
| gene pleiotropy | 10.2 | 0.0 | **34.3** | **54.5** | 0.6 | 0.3 |  |
| ePPID | 1.2 | 0.6 | 0.0 | 0.4 | **48.6** | **49.3** |  |
| betweenness | 0.0 | 1.7 | 0.7 | 0.3 | **49.7** | **47.5** |  |

Note: ^#^P<0.01; ^*^P<10^-3^; ^**^P<10^-6^; ^***^P<10^-9^. Bold indicates that the predictor variable contributes at least 20% to the corresponding principal component.

## Table S20B - Principal component regression analysis on six predictor variables and protein evolutionary rate for 723 yeast proteins in the “Combined-AP/MS” dataset.

|  | Principal Components | | | | | | |
| --- | --- | --- | --- | --- | --- | --- | --- |
|  | 1 | 2 | 3 | 4 | 5 | 6 | All |
| Percent variance explained in dN | 28.07*** | 7.13*** | 1.81* | 0.95* | 0.03 | 0.01 | 37.99*** |
| Percent contributions |  | | | | | | |
| protein abundance | **24.8** | 8.8 | 13.8 | **48.7** | 0.2 | 3.6 |  |
| CAI | **22.4** | 8.7 | **20.6** | **48.1** | 0.2 | 0.1 |  |
| gene dispensability | 7.5 | 0.2 | **25.1** | 2.0 | 5.7 | **59.5** |  |
| gene pleiotropy | 0.1 | **59.5** | **26.8** | 0.0 | 2.2 | 11.5 |  |
| ePPID | **23.6** | 15.1 | 7.5 | 0.3 | **52.2** | 1.3 |  |
| betweenness | **21.6** | 7.7 | 6.3 | 0.9 | **39.5** | **24.0** |  |

Note: ^#^P<0.01; ^*^P<10^-3^; ^**^P<10^-6^; ^***^P<10^-9^. Bold indicates that the predictor variable contributes at least 20% to the corresponding principal component.

## Table S20C - Principal component regression analysis on six predictor variables and protein evolutionary rate for 639 yeast proteins in the “LC-multiple” dataset.

|  | Principal Components | | | | | | |
| --- | --- | --- | --- | --- | --- | --- | --- |
|  | 1 | 2 | 3 | 4 | 5 | 6 | All |
| Percent variance explained in dN | 25.00*** | 8.12*** | 0.53 | 0.45 | 0.35 | 0.11 | 34.56*** |
| Percent contributions |  | | | | | | |
| protein abundance | 19.1 | **25.2** | 3.6 | 3.4 | **48.4** | 0.3 |  |
| CAI | 18.0 | **25.6** | 0.2 | 7.5 | **46.6** | 2.2 |  |
| gene dispensability | 12.2 | 0.0 | 16.6 | **67.1** | 1.0 | 3.0 |  |
| gene pleiotropy | 3.8 | **23.9** | **49.5** | **21.4** | 0.6 | 0.7 |  |
| ePPID | **26.8** | 9.6 | 6.1 | 0.2 | 0.6 | **56.7** |  |
| betweenness | **20.0** | 15.7 | **23.9** | 0.3 | 2.8 | **37.3** |  |

Note: ^#^P<0.01; ^*^P<10^-3^; ^**^P<10^-6^; ^***^P<10^-9^. Bold indicates that the predictor variable contributes at least 20% to the corresponding principal component.

## Table S20D - Principal component regression analysis on six predictor variables and protein evolutionary rate for 1,502 yeast proteins in the “Updated-HC” dataset.

|  | Principal Components | | | | | | |
| --- | --- | --- | --- | --- | --- | --- | --- |
|  | 1 | 2 | 3 | 4 | 5 | 6 | All |
| Percent variance explained in dN | 21.11*** | 15.87*** | 0.75* | 0.61* | 0.28^#^ | 0.14 | 38.76*** |
| Percent contributions |  | | | | | | |
| protein abundance | 14.1 | **34.6** | 0.0 | **46.2** | 5.0 | 0.0 |  |
| CAI | 9.7 | **40.5** | 0.4 | **38.6** | 9.6 | 1.2 |  |
| gene dispensability | 14.5 | 2.0 | 18.4 | 0.0 | 5.8 | **59.3** |  |
| gene pleiotropy | 13.3 | 4.3 | **75.2** | 0.0 | 0.0 | 7.2 |  |
| ePPID | **28.4** | 6.3 | 5.0 | 9.6 | **45.6** | 5.2 |  |
| betweenness | 19.9 | 12.3 | 1.0 | 5.6 | **34.0** | **27.1** |  |

Note: ^#^P<0.01; ^*^P<10^-3^; ^**^P<10^-6^; ^***^P<10^-9^. Bold indicates that the predictor variable contributes at least 20% to the corresponding principal component.

## Table S20E - Principal component regression analysis on six predictor variables and protein evolutionary rate for 913 yeast proteins in the “DIP-CORE” dataset.

|  | Principal Components | | | | | | |
| --- | --- | --- | --- | --- | --- | --- | --- |
|  | 1 | 2 | 3 | 4 | 5 | 6 | All |
| Percent variance explained in dN | 24.20*** | 11.33*** | 0.94* | 0.52^#^ | 0.21 | 0.14 | 37.32*** |
| Percent contributions |  | | | | | | |
| protein abundance | **20.4** | **26.5** | **51.2** | 1.8 | 0.1 | 0.0 |  |
| CAI | 14.3 | **34.5** | **47.1** | 0.8 | 2.1 | 1.3 |  |
| gene dispensability | 14.1 | 1.0 | 1.5 | 0.0 | **45.6** | **37.7** |  |
| gene pleiotropy | 10.0 | 8.3 | 0.0 | 0.1 | **51.5** | **30.1** |  |
| ePPID | **25.5** | 7.7 | 0.0 | **52.3** | 0.0 | 14.4 |  |
| betweenness | 15.7 | **22.1** | 0.2 | **45.0** | 0.6 | 16.4 |  |

Note: ^#^P<0.01; ^*^P<10^-3^; ^**^P<10^-6^; ^***^P<10^-9^. Bold indicates that the predictor variable contributes at least 20% to the corresponding principal component.

## Table S20F - Principal component regression analysis on six predictor variables and protein evolutionary rate for 1,698 yeast proteins in the “DIP-FULL” dataset.

|  | Principal Components | | | | | | |
| --- | --- | --- | --- | --- | --- | --- | --- |
|  | 1 | 2 | 3 | 4 | 5 | 6 | All |
| Percent variance explained in dN | 26.07*** | 10.95*** | 0.86* | 0.43* | 0.26^#^ | 0.21 | 38.78*** |
| Percent contributions |  | | | | | | |
| protein abundance | 17.8 | **30.8** | 0.1 | **39.7** | 11.6 | 0.0 |  |
| CAI | 14.1 | **36.6** | 0.9 | **32.3** | 15.2 | 0.8 |  |
| gene dispensability | 10.1 | 1.9 | **22.5** | 0.0 | 3.5 | **62.0** |  |
| gene pleiotropy | 11.6 | 6.5 | **70.2** | 0.0 | 0.4 | 11.4 |  |
| ePPID | **25.8** | 9.5 | 5.6 | 16.1 | **35.9** | 7.0 |  |
| betweenness | **20.6** | 14.7 | 0.7 | 11.8 | **33.4** | 18.8 |  |

Note: ^#^P<0.01; ^*^P<10^-3^; ^**^P<10^-6^; ^***^P<10^-9^. Bold indicates that the predictor variable contributes at least 20% to the corresponding principal component.

## Table S20G - Principal component regression analysis on six predictor variables and protein evolutionary rate for 566 yeast proteins in the “FYI” dataset.

|  | Principal Components | | | | | | |
| --- | --- | --- | --- | --- | --- | --- | --- |
|  | 1 | 2 | 3 | 4 | 5 | 6 | All |
| Percent variance explained in dN | 33.04*** | 2.33* | 1.68* | 0.71 | 0.29 | 0.20 | 38.24*** |
| Percent contributions |  | | | | | | |
| protein abundance | **33.4** | 10.2 | 3.3 | **49.8** | 2.8 | 0.5 |  |
| CAI | **30.8** | 13.7 | 0.6 | **49.1** | 3.2 | 2.6 |  |
| gene dispensability | 6.4 | 13.0 | **26.2** | 1.0 | 0.0 | **53.4** |  |
| gene pleiotropy | 0.3 | **22.6** | **33.2** | 0.0 | 0.8 | **43.2** |  |
| ePPID | **23.4** | 8.3 | 17.1 | 0.0 | **51.1** | 0.0 |  |
| betweenness | 5.7 | 32.2 | 19.7 | 0.0 | **42.2** | 0.2 |  |

Note: ^#^P<0.01; ^*^P<10^-3^; ^**^P<10^-6^; ^***^P<10^-9^. Bold indicates that the predictor variable contributes at least 20% to the corresponding principal component.

## Table S20H - Principal component regression analysis on six predictor variables and protein evolutionary rate for 306 yeast proteins in the “SIN” dataset.

|  | Principal Components | | | | | | |
| --- | --- | --- | --- | --- | --- | --- | --- |
|  | 1 | 2 | 3 | 4 | 5 | 6 | All |
| Percent variance explained in dN | 30.06*** | 1.40 | 0.53 | 0.39 | 0.38 | 0.08 | 32.83*** |
| Percent contributions |  | | | | | | |
| protein abundance | **44.2** | 2.0 | 0.1 | **50.1** | 1.3 | 2.3 |  |
| CAI | **41.5** | 5.1 | 2.0 | **47.7** | 2.7 | 0.9 |  |
| gene dispensability | 4.1 | 19.1 | **68.1** | 2.2 | 3.8 | 2.6 |  |
| gene pleiotropy | 1.5 | **64.3** | 9.9 | 0.0 | **22.6** | 1.7 |  |
| ePPID | 8.3 | 1.5 | 1.5 | 0.0 | **32.8** | **55.8** |  |
| betweenness | 0.3 | 8.0 | 18.3 | 0.0 | **36.7** | **36.6** |  |

Note: ^#^P<0.01; ^*^P<10^-3^; ^**^P<10^-6^; ^***^P<10^-9^. Bold indicates that the predictor variable contributes at least 20% to the corresponding principal component.

## Table S20I - Principal component regression analysis on six predictor variables and protein evolutionary rate for 1,776 yeast proteins in the “Eight-union” dataset.

|  | Principal Components | | | | | | |
| --- | --- | --- | --- | --- | --- | --- | --- |
|  | 1 | 2 | 3 | 4 | 5 | 6 | All |
| Percent variance explained in dN | 24.69*** | 11.97*** | 1.38*** | 0.58* | 0.17 | 0.07 | 38.86*** |
| Percent contributions |  | | | | | | |
| protein abundance | 16.2 | **32.2** | 0.0 | **39.0** | 0.3 | 12.4 |  |
| CAI | 13.1 | **37.5** | 0.7 | **42.8** | 0.6 | 5.2 |  |
| gene dispensability | 10.9 | 3.6 | 8.2 | 2.8 | **73.7** | 0.9 |  |
| gene pleiotropy | 11.8 | 7.8 | **78.6** | 0.2 | 1.4 | 0.2 |  |
| ePPID | **27.1** | 5.9 | 9.0 | 8.0 | 5.1 | **44.9** |  |
| betweenness | **20.9** | 13.1 | 3.5 | 7.2 | 18.9 | **36.4** |  |

Note: ^#^P<0.01; ^*^P<10^-3^; ^**^P<10^-6^; ^***^P<10^-9^. Bold indicates that the predictor variable contributes at least 20% to the corresponding principal component.

## Table S21 - The contribution of six predictor variables to the total variance of protein evolutionary rate explained by all six principal components in the nine protein interaction datasets.

| **Percent variance explained in dN** | *n* | protein abundance | CAI | gene dispensability | gene pleiotropy | ePPID | betweenness | All |
| --- | --- | --- | --- | --- | --- | --- | --- | --- |
| Y2H-union | 752 | 17.142 | 14.779 | 2.489 | 3.819 | 0.469 | 0.043 | 38.74 |
| Combined-AP/MS | 723 | 8.318 | 7.730 | 2.603 | 4.737 | 7.857 | 6.745 | 37.99 |
| LC-multiple | 639 | 7.037 | 6.769 | 3.458 | 3.263 | 7.574 | 6.457 | 34.56 |
| Updated-HC | 1,502 | 8.772 | 8.751 | 3.616 | 4.059 | 7.230 | 6.336 | 38.76 |
| DIP-CORE | 913 | 8.428 | 7.806 | 3.688 | 3.513 | 7.338 | 6.551 | 37.32 |
| DIP-FULL | 1,698 | 8.215 | 7.871 | 3.171 | 4.362 | 7.996 | 7.161 | 38.78 |
| FYI | 566 | 11.688 | 10.868 | 2.971 | 1.256 | 8.361 | 3.094 | 38.24 |
| SIN | 306 | 13.528 | 12.765 | 1.890 | 1.485 | 2.688 | 0.477 | 32.83 |
| Eight-union | 1,776 | 8.088 | 7.991 | 3.361 | 4.939 | 7.605 | 6.881 | 38.86 |

As can be seen, the contribution of ePPID to the total variance is more than all other predictor variables in the “LC-multiple” dataset. In the “Combined-AP/MS” and “DIP-FULL” datasets, the ePPID contribution is slightly less than that of protein abundance, but more than all other variables. In the “Updated-HC”, “DIP-CORE”, “FYI” and “Eight-union” datasets, the ePPID contribution is slightly less than protein abundance and CAI. However, in the “Y2H-union” and “SIN” datasets, the ePPID contribution is less than protein abundance and CAI.

## Table S22A - Principal component regression analysis on seven predictor variables and protein evolutionary rate for 752 yeast proteins in the “Y2H-union” dataset.

|  | Principal Components | | | | | | | |
| --- | --- | --- | --- | --- | --- | --- | --- | --- |
|  | 1 | 2 | 3 | 4 | 5 | 6 | 7 | All |
| Percent variance explained in dN | 39.91*** | 0.77^#^ | 0.55^#^ | 0.42 | 0.11 | 0.06 | 0.00 | 41.81*** |
| Percent contributions |  | | | | | | | |
| mRNA abundance | **31.6** | **21.5** | **43.6** | 1.3 | 0.7 | 0.0 | 1.3 |  |
| protein abundance | **31.8** | 11.1 | **55.6** | 0.4 | 0.4 | 0.0 | 0.6 |  |
| CAI | **29.7** | **60.6** | 0.5 | 6.5 | 0.9 | 0.6 | 1.3 |  |
| gene dispensability | 2.6 | 3.8 | 0.1 | **49.8** | **43.6** | 0.1 | 0.1 |  |
| gene pleiotropy | 3.7 | 0.0 | 0.1 | **41.1** | **53.9** | 0.8 | 0.4 |  |
| ePPID | 0.6 | 0.6 | 0.0 | 0.0 | 0.2 | **49.0** | **49.6** |  |
| betweenness | 0.0 | 2.4 | 0.0 | 0.9 | 0.4 | **49.5** | **46.8** |  |

Note: ^#^P<0.01; ^*^P<10^-3^; ^**^P<10^-6^; ^***^P<10^-9^. Bold indicates that the predictor variable contributes at least 20% to the corresponding principal component.

## Table S22B - Principal component regression analysis on seven predictor variables and protein evolutionary rate for 723 yeast proteins in the “Combined-AP/MS” dataset.

|  | Principal Components | | | | | | | |
| --- | --- | --- | --- | --- | --- | --- | --- | --- |
|  | 1 | 2 | 3 | 4 | 5 | 6 | 7 | All |
| Percent variance explained in dN | 34.48*** | 5.47*** | 0.85^#^ | 0.44 | 0.04 | 0.03 | 0.01 | 41.33*** |
| Percent contributions |  | | | | | | | |
| mRNA abundance | **24.6** | 1.4 | **22.6** | 5.5 | 0.5 | 6.8 | **38.6** |  |
| protein abundance | **23.1** | 4.1 | **61.6** | 5.4 | 1.5 | 2.3 | 2.0 |  |
| CAI | **22.5** | 3.6 | 10.0 | 10.2 | 0.9 | 1.6 | **51.2** |  |
| gene dispensability | 3.5 | 1.4 | 2.1 | **27.0** | **60.1** | 5.4 | 0.5 |  |
| gene pleiotropy | 0.0 | **64.7** | 0.0 | 19.6 | 13.2 | 2.5 | 0.0 |  |
| ePPID | 14.3 | 14.9 | 3.0 | 16.4 | 0.8 | **45.9** | 4.7 |  |
| betweenness | 11.9 | 9.8 | 0.7 | 15.8 | **23.2** | **35.6** | 3.0 |  |

Note: ^#^P<0.01; ^*^P<10^-3^; ^**^P<10^-6^; ^***^P<10^-9^. Bold indicates that the predictor variable contributes at least 20% to the corresponding principal component.

## Table S22C - Principal component regression analysis on seven predictor variables and protein evolutionary rate for 639 yeast proteins in the “LC-multiple” dataset.

|  | Principal Components | | | | | | | |
| --- | --- | --- | --- | --- | --- | --- | --- | --- |
|  | 1 | 2 | 3 | 4 | 5 | 6 | 7 | All |
| Percent variance explained in dN | 37.38*** | 0.59 | 0.47 | 0.46 | 0.13 | 0.12 | 0.04 | 39.19*** |
| Percent contributions |  | | | | | | | |
| mRNA abundance | **28.8** | 7.1 | 0.1 | **56.7** | 2.8 | 1.0 | 3.5 |  |
| protein abundance | **27.2** | **63.0** | 3.0 | 2.0 | 0.4 | 1.0 | 3.4 |  |
| CAI | **27.0** | **26.8** | 0.3 | **33.2** | 5.1 | 3.6 | 3.9 |  |
| gene dispensability | 5.4 | 1.2 | 16.9 | 0.0 | 2.7 | **69.3** | 4.5 |  |
| gene pleiotropy | 0.0 | 0.9 | **49.5** | 0.1 | 0.6 | **24.6** | **24.3** |  |
| ePPID | 8.1 | 0.0 | 6.0 | 4.6 | **53.1** | 0.2 | **27.9** |  |
| betweenness | 3.4 | 0.9 | **24.2** | 3.4 | **35.3** | 0.3 | **32.5** |  |

Note: ^#^P<0.01; ^*^P<10^-3^; ^**^P<10^-6^; ^***^P<10^-9^. Bold indicates that the predictor variable contributes at least 20% to the corresponding principal component.

## Table S22D - Principal component regression analysis on seven predictor variables and protein evolutionary rate for 1,502 yeast proteins in the “Updated-HC” dataset.

|  | Principal Components | | | | | | | |
| --- | --- | --- | --- | --- | --- | --- | --- | --- |
|  | 1 | 2 | 3 | 4 | 5 | 6 | 7 | All |
| Percent variance explained in dN | 36.72*** | 1.91*** | 0.99** | 0.29^#^ | 0.28^#^ | 0.21 | 0.14 | 40.55*** |
| Percent contributions |  | | | | | | | |
| mRNA abundance | **23.8** | 9.5 | 0.2 | **28.8** | 0.3 | 0.0 | **37.3** |  |
| protein abundance | **25.2** | 6.6 | 0.0 | 6.3 | 3.1 | 0.0 | **58.7** |  |
| CAI | **22.7** | 11.4 | 0.9 | **52.5** | 10.2 | 0.8 | 1.3 |  |
| gene dispensability | 6.5 | 9.9 | 19.5 | 0.1 | 5.8 | **58.2** | 0.0 |  |
| gene pleiotropy | 4.4 | 13.6 | **73.6** | 0.2 | 0.0 | 8.0 | 0.3 |  |
| ePPID | 11.8 | **22.6** | 4.8 | 7.6 | **46.2** | 5.4 | 1.6 |  |
| betweenness | 5.5 | **26.3** | 0.9 | 4.5 | **34.4** | **27.5** | 0.8 |  |

Note: ^#^P<0.01; ^*^P<10^-3^; ^**^P<10^-6^; ^***^P<10^-9^. Bold indicates that the predictor variable contributes at least 20% to the corresponding principal component.

## Table S22E - Principal component regression analysis on seven predictor variables and protein evolutionary rate for 913 yeast proteins in the “DIP-CORE” dataset.

|  | Principal Components | | | | | | | |
| --- | --- | --- | --- | --- | --- | --- | --- | --- |
|  | 1 | 2 | 3 | 4 | 5 | 6 | 7 | All |
| Percent variance explained in dN | 37.01*** | 1.17* | 0.99* | 0.57^#^ | 0.12 | 0.33 | 0.02 | 40.21*** |
| Percent contributions |  | | | | | | | |
| mRNA abundance | **26.1** | 6.5 | 18.0 | 4.0 | 0.2 | 0.5 | **44.8** |  |
| protein abundance | **26.6** | 4.4 | **60.9** | 6.9 | 0.0 | 0.1 | 1.1 |  |
| CAI | **23.9** | 9.9 | 13.8 | 2.9 | 1.2 | 0.4 | **47.8** |  |
| gene dispensability | 6.4 | 8.0 | 1.3 | 0.0 | **42.7** | **41.3** | 0.3 |  |
| gene pleiotropy | 2.7 | 15.8 | 0.1 | 0.0 | **55.1** | **26.4** | 0.0 |  |
| ePPID | 11.2 | **21.5** | 3.1 | **45.6** | 0.1 | 14.4 | 4.2 |  |
| betweenness | 3.1 | **33.9** | 3.0 | **40.5** | 0.8 | 17.0 | 1.8 |  |

Note: ^#^P<0.01; ^*^P<10^-3^; ^**^P<10^-6^; ^***^P<10^-9^. Bold indicates that the predictor variable contributes at least 20% to the corresponding principal component.

## Table S22F - Principal component regression analysis on seven predictor variables and protein evolutionary rate for 1,698 yeast proteins in the “DIP-FULL” dataset.

|  | Principal Components | | | | | | | |
| --- | --- | --- | --- | --- | --- | --- | --- | --- |
|  | 1 | 2 | 3 | 4 | 5 | 6 | 7 | All |
| Percent variance explained in dN | 36.49*** | 2.21*** | 1.03** | 0.37^#^ | 0.30^#^ | 0.14 | 0.0 | 40.53*** |
| Percent contributions |  | | | | | | | |
| mRNA abundance | **22.2** | 11.1 | 0.1 | 1.2 | 0.0 | **30.4** | **34.9** |  |
| protein abundance | **23.0** | 8.4 | 0.0 | **27.6** | 0.1 | 3.2 | **37.8** |  |
| CAI | **21.4** | 13.4 | 1.4 | **20.1** | 0.5 | **43.1** | 0.2 |  |
| gene dispensability | 5.6 | 6.3 | **22.8** | 3.2 | **61.6** | 0.0 | 0.4 |  |
| gene pleiotropy | 5.1 | 13.5 | **69.3** | 0.2 | 11.4 | 0.1 | 0.4 |  |
| ePPID | 13.8 | **21.0** | 5.7 | **24.3** | 7.3 | 13.7 | 14.3 |  |
| betweenness | 8.9 | **26.2** | 0.7 | **23.4** | 19.2 | 9.5 | 12.1 |  |

Note: ^#^P<0.01; ^*^P<10^-3^; ^**^P<10^-6^; ^***^P<10^-9^. Bold indicates that the predictor variable contributes at least 20% to the corresponding principal component.

## Table S22G - Principal component regression analysis on seven predictor variables and protein evolutionary rate for 566 yeast proteins in the “FYI” dataset.

|  | Principal Components | | | | | | | |
| --- | --- | --- | --- | --- | --- | --- | --- | --- |
|  | 1 | 2 | 3 | 4 | 5 | 6 | 7 | All |
| Percent variance explained in dN | 39.31*** | 1.71* | 0.87^#^ | 0.32 | 0.17 | 0.13 | 0.00 | 42.52*** |
| Percent contributions |  | | | | | | | |
| mRNA abundance | **30.7** | 0.0 | 9.1 | 0.1 | **58.9** | 0.1 | 1.1 |  |
| protein abundance | **27.7** | 3.4 | **63.0** | 3.6 | 0.6 | 0.2 | 1.6 |  |
| CAI | **27.8** | 0.7 | **26.1** | 3.8 | **36.3** | 2.0 | 3.4 |  |
| gene dispensability | 1.6 | **25.9** | 0.9 | 0.0 | 0.2 | **53.7** | 17.7 |  |
| gene pleiotropy | 0.2 | **33.3** | 0.2 | 0.8 | 0.3 | **43.7** | **21.4** |  |
| ePPID | 11.4 | 17.0 | 0.8 | **49.7** | 3.5 | 0.0 | 17.5 |  |
| betweenness | 0.6 | 19.7 | 0.0 | **41.9** | 0.2 | 0.3 | **37.3** |  |

Note: ^#^P<0.01; ^*^P<10^-3^; ^**^P<10^-6^; ^***^P<10^-9^. Bold indicates that the predictor variable contributes at least 20% to the corresponding principal component.

## Table S22H - Principal component regression analysis on seven predictor variables and protein evolutionary rate for 306 yeast proteins in the “SIN” dataset.

|  | Principal Components | | | | | | | |
| --- | --- | --- | --- | --- | --- | --- | --- | --- |
|  | 1 | 2 | 3 | 4 | 5 | 6 | 7 | All |
| Percent variance explained in dN | 32.56*** | 0.94 | 0.56 | 0.50 | 0.29 | 0.11 | 0.02 | 34.97*** |
| Percent contributions |  | | | | | | | |
| mRNA abundance | **32.9** | 0.3 | 0.0 | 0.0 | 5.9 | 0.1 | **60.7** |  |
| protein abundance | **29.4** | 0.8 | 0.2 | 1.0 | **63.5** | 2.8 | 2.3 |  |
| CAI | **29.2** | 3.2 | 1.9 | 2.3 | **28.6** | 1.3 | **33.4** |  |
| gene dispensability | 2.3 | **20.0** | **68.5** | 4.0 | 1.5 | 2.6 | 1.1 |  |
| gene pleiotropy | 0.8 | **65.4** | 9.7 | **22.3** | 0.0 | 1.8 | 0.0 |  |
| ePPID | 5.3 | 1.9 | 1.5 | **33.5** | 0.4 | **55.0** | 2.4 |  |
| betweenness | 0.1 | 8.3 | 18.3 | **36.9** | 0.0 | **36.4** | 0.0 |  |

Note: ^#^P<0.01; ^*^P<10^-3^; ^**^P<10^-6^; ^***^P<10^-9^. Bold indicates that the predictor variable contributes at least 20% to the corresponding principal component.

## Table S22I - Principal component regression analysis on seven predictor variables and protein evolutionary rate for 1,776 yeast proteins in the “Eight-union” dataset.

|  | Principal Components | | | | | | | |
| --- | --- | --- | --- | --- | --- | --- | --- | --- |
|  | 1 | 2 | 3 | 4 | 5 | 6 | 7 | All |
| Percent variance explained in dN | 35.31*** | 3.19*** | 1.59*** | 0.35^#^ | 0.16 | 0.07 | 0.04 | 40.71*** |
| Percent contributions |  | | | | | | | |
| mRNA abundance | **20.9** | 12.5 | 0.1 | 0.3 | 0.0 | **32.1** | **34.1** |  |
| protein abundance | **21.4** | 9.7 | 0.1 | 15.7 | 0.3 | 1.7 | **51.2** |  |
| CAI | **20.1** | 14.5 | 1.1 | **28.2** | 0.7 | **35.1** | 0.3 |  |
| gene dispensability | 6.0 | 8.3 | 8.2 | 3.7 | **73.8** | 0.0 | 0.0 |  |
| gene pleiotropy | 5.4 | 14.5 | **77.8** | 0.5 | 1.4 | 0.0 | 0.4 |  |
| ePPID | 16.3 | 16.2 | 9.1 | **27.8** | 5.1 | 18.1 | 7.5 |  |
| betweenness | 9.8 | **24.4** | 3.6 | **23.8** | 18.9 | 13.0 | 6.5 |  |

Note: ^#^P<0.01; ^*^P<10^-3^; ^**^P<10^-6^; ^***^P<10^-9^. Bold indicates that the predictor variable contributes at least 20% to the corresponding principal component.

## Table S23 - The contribution of seven predictor variables to the total variance of protein evolutionary rate explained by all seven principal components in the nine protein interaction datasets.

| **Percent variance explained in dN** | *n* | mRNA abundance | protein abundance | CAI | gene dispensability | gene pleiotropy | ePPID | betweenness | All |
| --- | --- | --- | --- | --- | --- | --- | --- | --- | --- |
| Y2H-union | 752 | 13.014 | 13.099 | 12.359 | 1.320 | 1.703 | 0.259 | 0.057 | 41.81 |
| Combined-AP/MS | 723 | 8.787 | 8.745 | 8.104 | 1.454 | 3.644 | 5.845 | 4.745 | 41.33 |
| LC-multiple | 639 | 11.058 | 10.583 | 10.428 | 2.195 | 0.283 | 3.166 | 1.476 | 39.19 |
| Updated-HC | 1,502 | 9.077 | 9.484 | 8.765 | 2.908 | 2.606 | 4.995 | 2.715 | 40.55 |
| DIP-CORE | 913 | 9.946 | 10.553 | 9.141 | 2.672 | 1.328 | 4.721 | 1.849 | 40.21 |
| DIP-FULL | 1,698 | 8.411 | 8.671 | 8.254 | 2.622 | 2.892 | 5.694 | 3.986 | 40.53 |
| FYI | 566 | 12.231 | 11.506 | 11.241 | 1.152 | 0.721 | 4.947 | 0.720 | 42.52 |
| SIN | 306 | 10.757 | 9.763 | 9.654 | 1.345 | 1.028 | 1.988 | 0.435 | 34.97 |
| Eight-union | 1,776 | 7.813 | 7.944 | 7.710 | 2.657 | 3.610 | 6.545 | 4.427 | 40.71 |

As can be seen, the contribution of ePPID to the total variance is slightly less than or less than mRNA abundance, protein abundance and CAI, but more than the other three predictor variables in all the nine datasets except “Y2H-union”.

## Table S24A - Statistical significance for the effect of co-expressed versus non-co-expressed proteins in the high-PPID bin.

| **Protein interaction datasets** | CPHB | NCPHB | P_wilcoxon_ | P_ancova_ |
| --- | --- | --- | --- | --- |
| Y2H-union | 0.131 | 0.154 | 9.15e-02 | 6.77e-02 |
| Combined-AP/MS | 0.0938 | 0.169 | **4.48e-05** | **1.24e-05** |
| LC-multiple | 0.103 | 0.124 | **3.04e-02** | 1.59e-01 |
| Updated-HC | 0.0996 | 0.157 | **1.61e-10** | **7.62e-11** |
| DIP-CORE | 0.120 | 0.143 | **8.65e-03** | 6.21e-02 |
| DIP-FULL | 0.0845 | 0.132 | **1.74e-07** | **3.30e-07** |
| FYI | 0.0776 | 0.124 | **2.13e-05** | **8.62e-03** |
| SIN | 0.0615 | 0.101 | **5.33e-03** | **1.29e-02** |
| Eight-union | 0.0881 | 0.133 | **4.91e-07** | **4.06e-08** |

The mean evolutionary rate dN are calculated for non-co-expressed and co-expressed proteins in the high-PPID bin. P_wilcoxon_ is the statistical significance indicating whether co-expressed proteins evolve more slowly than non-co-expressed proteins in the high-PPID bin, as calculated by one-sided Wilcoxon rank sum test. P_ancova_ is the statistical significance for the effect of co-expressed versus non-co-expressed proteins in the high-PPID bin, as calculated by analysis of covariance (ANCOVA) when controlling for the covariate of PPID. Bold indicates that either the P_wilcoxon_ or P_ancova_ is significant at the statistical significance level of 0.05. CPHB represents co-expressed proteins in the high-PPID bin, and NCPHB represents non-co-expressed proteins in the high-PPID bin.

## Table S24B - Statistical significance for the effect of co-expressed versus non-co-expressed proteins in the medium-PPID bin.

| **Protein interaction datasets** | CPMB | NCPMB | P_wilcoxon_ | P_ancova_ |
| --- | --- | --- | --- | --- |
| Y2H-union | 0.159 | 0.172 | 1.05e-01 | 1.45e-01 |
| Combined-AP/MS | 0.102 | 0.170 | **9.42e-10** | **1.62e-11** |
| LC-multiple | 0.125 | 0.179 | **6.27e-04** | **4.68e-04** |
| Updated-HC | 0.152 | 0.180 | **2.19e-05** | **1.60e-03** |
| DIP-CORE | 0.130 | 0.170 | **4.74e-05** | **2.95e-03** |
| DIP-FULL | 0.147 | 0.174 | **1.79e-05** | **2.78e-04** |
| FYI | 0.115 | 0.130 | 1.59e-01 | 2.98e-01 |
| SIN | 0.0937 | 0.137 | **2.08e-03** | **8.31e-03** |
| Eight-union | 0.147 | 0.177 | **5.96e-08** | **1.98e-06** |

The mean evolutionary rate dN are calculated for non-co-expressed and co-expressed proteins in the medium-PPID bin. P_wilcoxon_ is the statistical significance indicating whether co-expressed proteins evolve more slowly than non-co-expressed proteins in the medium-PPID bin, as calculated by one-sided Wilcoxon rank sum test. P_ancova_ is the statistical significance for the effect of co-expressed versus non-co-expressed proteins in the medium-PPID bin, as calculated by analysis of covariance (ANCOVA) when controlling for the covariate of PPID. Bold indicates that either the P_wilcoxon_ or P_ancova_ is significant at the statistical significance level of 0.05. CPMB represents co-expressed proteins in the medium-PPID bin, and NCPMB represents non-co-expressed proteins in the medium-PPID bin.

## Table S24C - Statistical significance for the effect of co-expressed versus non-co-expressed proteins in the low-PPID bin.

| **Protein interaction datasets** | CPLB | NCPLB | P_wilcoxon_ | P_ancova_ |
| --- | --- | --- | --- | --- |
| Y2H-union | 0.143 | 0.186 | **7.12e-05** | **2.15e-05** |
| Combined-AP/MS | 0.138 | 0.189 | **3.11e-05** | **1.41e-05** |
| LC-multiple | 0.144 | 0.189 | **6.14e-05** | **9.33e-05** |
| Updated-HC | 0.173 | 0.191 | **7.57e-03** | **2.53e-02** |
| DIP-CORE | 0.151 | 0.193 | **1.02e-05** | **6.84e-07** |
| DIP-FULL | 0.177 | 0.191 | **4.16e-02** | **4.43e-02** |
| FYI | 0.143 | 0.180 | **2.79e-04** | **8.60e-04** |
| SIN | 0.111 | 0.129 | **4.53e-02** | 1.79e-01 |
| Eight-union | 0.179 | 0.197 | **3.77e-02** | **2.73e-02** |

The mean evolutionary rate dN are calculated for non-co-expressed and co-expressed proteins in the low-PPID bin. P_wilcoxon_ is the statistical significance indicating whether co-expressed proteins evolve more slowly than non-co-expressed proteins in the low-PPID bin, as calculated by one-sided Wilcoxon rank sum test. P_ancova_ is the statistical significance for the effect of co-expressed versus non-co-expressed proteins in the low-PPID bin, as calculated by analysis of covariance (ANCOVA) when controlling for the covariate of PPID. Bold indicates that either the P_wilcoxon_ or P_ancova_ is significant at the statistical significance level of 0.05. CPLB represents co-expressed proteins in the low-PPID bin, and NCPLB represents non-co-expressed proteins in the low-PPID bin.

## Table S25 - The network properties of the “SIN” and “Updated-SIN” datasets.

| **Protein interaction datasets** | # proteins | # interactions | # non-co-expressed interactions (percentage) | Average degree | Average clustering coefficient |
| --- | --- | --- | --- | --- | --- |
| SIN | 873 | 1,269 | 270(21.3%) | 2.91 | 0.59 |
| Updated-SIN | 1,178 | 2,195 | 564(25.7%) | 3.73 | 0.54 |

The value in the parentheses shown in column 4 indicates the percentage of non-co-expressed protein interactions in the corresponding dataset.

## Table S26 - Spearman correlation of PPID, ePPID, nePPID and betweenness with protein evolutionary rate.

| **Protein interaction datasets** | *n* | PPID vs. dN | ePPID vs. dN | nePPID vs. dN | betweenness vs. dN |
| --- | --- | --- | --- | --- | --- |
|  |  | *rho*(*p*) | *rho*(*p*) | *rho*(*p*) | *rho*(*p*) |
| SIN | 436 | **-0.234(8.10e-07)** | **-0.339(3.30e-13)** | **0.167(4.47e-04)** | **-0.107(2.53e-02)** |
| Updated-SIN | 590 | **-0.170(3.19e-05)** | **-0.280(4.35e-12)** | **0.134(1.10e-03)** | **-0.105(1.08e-02)** |

dN represents protein evolutionary rate measured by non-synonymous substitutions. *n* is the number of proteins for which both PPID and protein evolutionary rate are available. *rho* is Spearman rank correlation coefficient, and *p* is the corresponding statistical significance. Bold indicates that *p* is significant at the statistical significance level of 0.05.

## Table S27 - Spearman correlation and partial Spearman correlation of PPID and ePPID with protein evolutionary rate in the “SIN” and “Updated-SIN” datasets.

| **Protein interaction datasets** | *n* | PPID vs. dN | PPID vs. Abundance | PPID vs. dN control for abundance | ePPID vs. dN | ePPID vs. Abundance | ePPID vs. dN control for abundance |
| --- | --- | --- | --- | --- | --- | --- | --- |
|  |  | *rho*(*p*) | *rho*(*p*) | *rho*(*p*) | *rho*(*p*) | *rho*(*p*) | *rho*(*p*) |
| SIN | 320 | **-0.146**  **(8.72e-03)** | 0.0138  (8.06e-01) | **-0.163**  **(3.35e-03)** | **-0.253**  **(4.61e-06)** | **0.182**  **(1.09e-03)** | **-0.189**  **(6.12e-04)** |
| Updated-SIN | 439 | **-0.111**  **(2.02e-02)** | 0.00813  (8.65e-01) | **-0.125**  **(8.68e-03)** | **-0.220**  **(3.20e-06)** | **0.203**  **(1.86e-05)** | **-0.137**  **(3.77e-03)** |

dN represents protein evolutionary rate measured by non-synonymous substitutions. *n* is the number of proteins for which PPID, protein evolutionary rate and abundance data are all available. *rho* is Spearman rank correlation coefficient, and *p* is the corresponding statistical significance. Bold indicates that *p* is significant at the statistical significance level of 0.05.

## Table S28 - Principal component regression analysis on six predictor variables and protein evolutionary rate for 424 yeast proteins in the “Updated-SIN” dataset.

|  | Principal Components | | | | | | |
| --- | --- | --- | --- | --- | --- | --- | --- |
|  | 1 | 2 | 3 | 4 | 5 | 6 | All |
| Percent variance explained in dN | 28.11*** | 1.87* | 1.40^#^ | 0.93 | 0.51 | 0.05 | 32.86*** |
| Percent contributions |  | | | | | | |
| mRNA abundance | **35.4** | 8.2 | 4.1 | 0.2 | **52.0** | 0.0 |  |
| protein abundance | **31.8** | 11.9 | 6.6 | 0.0 | **46.3** | 3.3 |  |
| gene dispensability | 9.1 | 0.2 | **51.9** | **36.6** | 0.0 | 2.3 |  |
| gene pleiotropy | 0.0 | 17.9 | **35.6** | **46.3** | 0.0 | 0.1 |  |
| ePPID | 18.2 | **21.2** | 1.7 | 4.8 | 1.5 | **52.6** |  |
| betweenness | 5.4 | **40.6** | 0.1 | 12.1 | 0.1 | **41.7** |  |

Note: ^#^P<0.01; ^*^P<10^-3^; ^**^P<10^-6^; ^***^P<10^-9^. Bold indicates that the predictor variable contributes at least 20% to the corresponding principal component.

## Table S29 - The contribution of six predictor variables to the total variance of protein evolutionary rate explained by all the six principal components in the “SIN” and “Updated-SIN” datasets.

| **Percent variance explained in dN** | *n* | mRNA abundance | protein abundance | gene dispensability | gene pleiotropy | ePPID | betweenness | All |
| --- | --- | --- | --- | --- | --- | --- | --- | --- |
| SIN | 306 | 12.651 | 11.334 | 3.117 | 0.872 | 4.322 | 0.686 | 32.98 |
| Updated-SIN | 424 | 10.435 | 9.504 | 3.641 | 1.276 | 5.601 | 2.408 | 32.86 |

*n* is the number of proteins for which all the six predictor variables and protein evolutionary rate are available.

## Table S30 - Correspondence of singlish-interface and multi-interface hubs to non-co-expressed and co-expressed hubs in the “Updated-SIN” dataset.

| **Hub class** | Non-co-expressed hubs (113) | Co-expressed hubs (203) |
| --- | --- | --- |
| Singlish-interface hubs (98) | 57 | 41 |
| Multi-interface hubs (218) | 56 | 162 |

The number in parentheses indicates the number of the corresponding hub class.

## Table S31A - Principal component regression analysis on six predictor variables and protein evolutionary rate for 752 yeast proteins in the “Y2H-union” dataset.

|  | Principal Components | | | | | | |
| --- | --- | --- | --- | --- | --- | --- | --- |
|  | 1 | 2 | 3 | 4 | 5 | 6 | All |
| Percent variance explained in dN | 33.94*** | 0.74^#^ | 0.46 | 0.38 | 0.23 | 0.00 | 35.74*** |
| Percent contributions |  | | | | | | |
| mRNA abundance | **35.9** | 1.6 | **49.0** | 13.3 | 0.0 | 0.3 |  |
| protein abundance | **37.0** | 2.1 | **50.7** | 10.1 | 0.0 | 0.0 |  |
| gene dispensability | 10.3 | 2.0 | 0.0 | **37.3** | **50.0** | 0.4 |  |
| gene pleiotropy | 11.3 | 0.1 | 0.1 | **38.9** | **49.2** | 0.4 |  |
| ePPID | 4.4 | **45.0** | 0.0 | 0.2 | 0.3 | **49.9** |  |
| betweenness | 1.1 | **49.2** | 0.1 | 0.1 | 0.4 | **49.0** |  |

Note: ^#^P<0.01; ^*^P<10^-3^; ^**^P<10^-6^; ^***^P<10^-9^. Bold indicates that the predictor variable contributes at least 20% to the corresponding principal component. ePPID and betweenness are added by a smaller constant of 0.2.

## Table S31B - Principal component regression analysis on six predictor variables and protein evolutionary rate for 752 yeast proteins in the “Y2H-union” dataset.

|  | Principal Components | | | | | | |
| --- | --- | --- | --- | --- | --- | --- | --- |
|  | 1 | 2 | 3 | 4 | 5 | 6 | All |
| Percent variance explained in dN | 34.13*** | 0.55 | 0.46 | 0.38 | 0.23 | 0.00 | 35.74*** |
| Percent contributions |  | | | | | | |
| mRNA abundance | **36.2** | 1.2 | **49.1** | 13.3 | 0.0 | 0.2 |  |
| protein abundance | **37.3** | 1.7 | **50.7** | 10.2 | 0.0 | 0.0 |  |
| gene dispensability | 10.4 | 2.0 | 0.0 | **37.2** | **49.9** | 0.4 |  |
| gene pleiotropy | 11.3 | 0.0 | 0.1 | **38.9** | **49.2** | 0.4 |  |
| ePPID | 3.9 | **45.4** | 0.0 | 0.3 | 0.4 | **49.9** |  |
| betweenness | 0.8 | **49.6** | 0.1 | 0.1 | 0.4 | **49.0** |  |

Note: ^#^P<0.01; ^*^P<10^-3^; ^**^P<10^-6^; ^***^P<10^-9^. Bold indicates that the predictor variable contributes at least 20% to the corresponding principal component. ePPID and betweenness are added by a smaller constant of 0.1.

## Table S31C - Principal component regression analysis on six predictor variables and protein evolutionary rate for 752 yeast proteins in the “Y2H-union” dataset.

|  | Principal Components | | | | | | |
| --- | --- | --- | --- | --- | --- | --- | --- |
|  | 1 | 2 | 3 | 4 | 5 | 6 | All |
| Percent variance explained in dN | 34.25*** | 0.46 | 0.42 | 0.38 | 0.23 | 0.00 | 35.74*** |
| Percent contributions |  | | | | | | |
| mRNA abundance | **36.4** | **49.1** | 1.0 | 13.3 | 0.0 | 0.2 |  |
| protein abundance | **37.6** | **50.7** | 1.5 | 10.2 | 0.0 | 0.0 |  |
| gene dispensability | 10.5 | 0.0 | 2.0 | **37.1** | **49.9** | 0.5 |  |
| gene pleiotropy | 11.3 | 0.1 | 0.0 | **38.9** | **49.2** | 0.4 |  |
| ePPID | 3.6 | 0.0 | **45.7** | 0.3 | 0.5 | **49.9** |  |
| betweenness | 0.7 | 0.1 | **49.8** | 0.1 | 0.4 | **49.0** |  |

Note: ^#^P<0.01; ^*^P<10^-3^; ^**^P<10^-6^; ^***^P<10^-9^. Bold indicates that the predictor variable contributes at least 20% to the corresponding principal component. ePPID and betweenness are added by a smaller constant of 0.05.

## Table S31D - Principal component regression analysis on six predictor variables and protein evolutionary rate for 752 yeast proteins in the “Y2H-union” dataset.

|  | Principal Components | | | | | | |
| --- | --- | --- | --- | --- | --- | --- | --- |
|  | 1 | 2 | 3 | 4 | 5 | 6 | All |
| Percent variance explained in dN | 29.78*** | 4.91*** | 0.47 | 0.36 | 0.25 | 0.00 | 35.77*** |
| Percent contributions |  | | | | | | |
| mRNA abundance | **35.0** | 3.0 | **49.2** | 12.8 | 0.0 | 0.0 |  |
| protein abundance | **36.2** | 3.4 | **50.7** | 9.7 | 0.0 | 0.0 |  |
| gene dispensability | 9.5 | 2.0 | 0.0 | **37.8** | **50.6** | 0.0 |  |
| gene pleiotropy | 9.4 | 1.7 | 0.1 | **39.4** | **49.3** | 0.1 |  |
| ePPID | 4.0 | **45.9** | 0.0 | 0.1 | 0.1 | **50.0** |  |
| betweenness | 6.0 | **44.0** | 0.0 | 0.2 | 0.0 | **49.8** |  |

Note: ^#^P<0.01; ^*^P<10^-3^; ^**^P<10^-6^; ^***^P<10^-9^. Bold indicates that the predictor variable contributes at least 20% to the corresponding principal component. ePPID and betweenness are not log transformed.

## Table S32A - Principal component regression analysis on six predictor variables and protein evolutionary rate for 723 yeast proteins in the “Combined-AP/MS” dataset.

|  | Principal Components | | | | | | |
| --- | --- | --- | --- | --- | --- | --- | --- |
|  | 1 | 2 | 3 | 4 | 5 | 6 | All |
| Percent variance explained in dN | 27.22*** | 8.01*** | 0.97* | 0.90^#^ | 0.78^#^ | 0.03 | 37.91*** |
| Percent contributions |  | | | | | | |
| mRNA abundance | **24.3** | 9.5 | 14.1 | **46.1** | 0.3 | 5.8 |  |
| protein abundance | **22.7** | 16.5 | 12.9 | **39.7** | 0.0 | 8.1 |  |
| gene dispensability | 8.6 | 2.0 | **21.7** | 1.3 | **62.1** | 4.3 |  |
| gene pleiotropy | 0.0 | **36.9** | **40.7** | 0.0 | 19.8 | 2.6 |  |
| ePPID | **24.8** | 16.8 | 4.0 | 9.3 | 0.0 | **45.1** |  |
| betweenness | 19.5 | 18.3 | 6.7 | 3.6 | 17.8 | **34.1** |  |

Note: ^#^P<0.01; ^*^P<10^-3^; ^**^P<10^-6^; ^***^P<10^-9^. Bold indicates that the predictor variable contributes at least 20% to the corresponding principal component. ePPID and betweenness are added by a smaller constant of 0.2.

## Table S32B - Principal component regression analysis on six predictor variables and protein evolutionary rate for 723 yeast proteins in the “Combined-AP/MS” dataset.

|  | Principal Components | | | | | | |
| --- | --- | --- | --- | --- | --- | --- | --- |
|  | 1 | 2 | 3 | 4 | 5 | 6 | All |
| Percent variance explained in dN | 27.26*** | 7.77*** | 1.11* | 0.92^#^ | 0.83^#^ | 0.01 | 37.90*** |
| Percent contributions |  | | | | | | |
| mRNA abundance | **24.3** | 9.0 | 14.6 | **47.3** | 0.3 | 4.4 |  |
| protein abundance | **22.8** | 15.9 | 13.4 | **41.3** | 0.1 | 6.5 |  |
| gene dispensability | 8.7 | 2.5 | **20.8** | 1.0 | **63.2** | 3.8 |  |
| gene pleiotropy | 0.0 | **37.2** | **40.1** | 0.0 | **20.6** | 2.1 |  |
| ePPID | **24.5** | 16.6 | 4.3 | 7.5 | 0.0 | **47.0** |  |
| betweenness | 19.7 | 18.9 | 6.8 | 2.8 | 15.8 | **36.1** |  |

Note: ^#^P<0.01; ^*^P<10^-3^; ^**^P<10^-6^; ^***^P<10^-9^. Bold indicates that the predictor variable contributes at least 20% to the corresponding principal component. ePPID and betweenness are added by a smaller constant of 0.1.

## Table S32C - Principal component regression analysis on six predictor variables and protein evolutionary rate for 723 yeast proteins in the “Combined-AP/MS” dataset.

|  | Principal Components | | | | | | |
| --- | --- | --- | --- | --- | --- | --- | --- |
|  | 1 | 2 | 3 | 4 | 5 | 6 | All |
| Percent variance explained in dN | 27.34*** | 7.54*** | 1.21* | 0.93^#^ | 0.87^#^ | 0.00 | 37.89*** |
| Percent contributions |  | | | | | | |
| mRNA abundance | **24.4** | 8.6 | 14.9 | **48.5** | 0.4 | 3.3 |  |
| protein abundance | **22.9** | 15.3 | 13.8 | **42.9** | 0.1 | 5.0 |  |
| gene dispensability | 8.8 | 2.9 | **20.1** | 0.8 | **64.2** | 3.3 |  |
| gene pleiotropy | 0.0 | **37.4** | **39.7** | 0.0 | **21.2** | 1.7 |  |
| ePPID | **24.2** | 16.7 | 4.6 | 5.9 | 0.1 | **48.7** |  |
| betweenness | 19.7 | 19.2 | 6.9 | 2.0 | 14.0 | **38.2** |  |

Note: ^#^P<0.01; ^*^P<10^-3^; ^**^P<10^-6^; ^***^P<10^-9^. Bold indicates that the predictor variable contributes at least 20% to the corresponding principal component. ePPID and betweenness are added by a smaller constant of 0.05.

## Table S32D - Principal component regression analysis on six predictor variables and protein evolutionary rate for 723 yeast proteins in the “Combined-AP/MS” dataset.

|  | Principal Components | | | | | | |
| --- | --- | --- | --- | --- | --- | --- | --- |
|  | 1 | 2 | 3 | 4 | 5 | 6 | All |
| Percent variance explained in dN | 30.16*** | 4.73*** | 1.31* | 1.24* | 0.68^#^ | 0.12 | 38.24*** |
| Percent contributions |  | | | | | | |
| mRNA abundance | **34.1** | 6.9 | 1.3 | 4.3 | **53.1** | 0.2 |  |
| protein abundance | **29.8** | 10.4 | 0.6 | 13.2 | **43.7** | 2.3 |  |
| gene dispensability | 6.1 | **35.8** | **34.3** | 5.4 | 0.5 | 17.9 |  |
| gene pleiotropy | 0.7 | 19.4 | **60.4** | 0.4 | 0.0 | 19.2 |  |
| ePPID | **21.3** | **21.6** | 0.2 | 9.1 | 2.6 | **45.2** |  |
| betweenness | 8.0 | 5.9 | 3.1 | **67.7** | 0.1 | 15.3 |  |

Note: ^#^P<0.01; ^*^P<10^-3^; ^**^P<10^-6^; ^***^P<10^-9^. Bold indicates that the predictor variable contributes at least 20% to the corresponding principal component. ePPID and betweenness are not log transformed.

## Table S33A - Principal component regression analysis on six predictor variables and protein evolutionary rate for 639 yeast proteins in the “LC-multiple” dataset.

|  | Principal Components | | | | | | |
| --- | --- | --- | --- | --- | --- | --- | --- |
|  | 1 | 2 | 3 | 4 | 5 | 6 | All |
| Percent variance explained in dN | 26.88*** | 7.44*** | 1.44* | 0.97^#^ | 0.31 | 0.22 | 37.26*** |
| Percent contributions |  | | | | | | |
| mRNA abundance | **20.4** | **22.9** | **49.6** | 6.0 | 0.0 | 1.0 |  |
| protein abundance | 19.7 | **22.7** | **43.1** | 6.4 | 1.6 | 6.6 |  |
| gene dispensability | 13.0 | 0.0 | 0.5 | **65.1** | **20.0** | 1.4 |  |
| gene pleiotropy | 3.0 | **24.9** | 0.9 | **22.4** | **48.6** | 0.3 |  |
| ePPID | **27.0** | 9.8 | 4.8 | 0.1 | 5.9 | **52.4** |  |
| betweenness | 17.0 | 19.7 | 1.2 | 0.0 | **23.9** | **38.4** |  |

Note: ^#^P<0.01; ^*^P<10^-3^; ^**^P<10^-6^; ^***^P<10^-9^. Bold indicates that the predictor variable contributes at least 20% to the corresponding principal component. ePPID and betweenness are added by a smaller constant of 0.2.

## Table S33B - Principal component regression analysis on six predictor variables and protein evolutionary rate for 639 yeast proteins in the “LC-multiple” dataset.

|  | Principal Components | | | | | | |
| --- | --- | --- | --- | --- | --- | --- | --- |
|  | 1 | 2 | 3 | 4 | 5 | 6 | All |
| Percent variance explained in dN | 27.43*** | 6.90*** | 1.40* | 0.94^#^ | 0.32 | 0.26 | 37.24*** |
| Percent contributions |  | | | | | | |
| mRNA abundance | **21.4** | **22.0** | **50.1** | 6.0 | 0.0 | 0.5 |  |
| protein abundance | **20.7** | **21.7** | **44.3** | 6.3 | 1.6 | 5.3 |  |
| gene dispensability | 13.0 | 0.0 | 0.4 | **65.6** | 19.9 | 1.1 |  |
| gene pleiotropy | 2.8 | **25.6** | 0.8 | **22.0** | **48.6** | 0.2 |  |
| ePPID | **25.9** | 10.4 | 3.6 | 0.1 | 6.8 | **53.2** |  |
| betweenness | 16.3 | **20.3** | 0.7 | 0.0 | **23.0** | **39.7** |  |

Note: ^#^P<0.01; ^*^P<10^-3^; ^**^P<10^-6^; ^***^P<10^-9^. Bold indicates that the predictor variable contributes at least 20% to the corresponding principal component. ePPID and betweenness are added by a smaller constant of 0.1.

## Table S33C - Principal component regression analysis on six predictor variables and protein evolutionary rate for 639 yeast proteins in the “LC-multiple” dataset.

|  | Principal Components | | | | | | |
| --- | --- | --- | --- | --- | --- | --- | --- |
|  | 1 | 2 | 3 | 4 | 5 | 6 | All |
| Percent variance explained in dN | 28.00*** | 6.33*** | 1.36* | 0.91^#^ | 0.34 | 0.29 | 37.22*** |
| Percent contributions |  | | | | | | |
| mRNA abundance | **22.4** | **21.0** | **50.4** | 6.0 | 0.0 | 0.2 |  |
| protein abundance | **21.7** | **20.7** | **45.2** | 6.3 | 1.7 | 4.4 |  |
| gene dispensability | 13.1 | 0.0 | 0.3 | **66.1** | 19.8 | 0.7 |  |
| gene pleiotropy | 2.5 | **26.3** | 0.7 | **21.7** | **48.7** | 0.1 |  |
| ePPID | **24.8** | 11.0 | 2.9 | 0.0 | 8.0 | **53.3** |  |
| betweenness | 15.5 | **21.1** | 0.5 | 0.0 | **21.8** | **41.2** |  |

Note: ^#^P<0.01; ^*^P<10^-3^; ^**^P<10^-6^; ^***^P<10^-9^. Bold indicates that the predictor variable contributes at least 20% to the corresponding principal component. ePPID and betweenness are added by a smaller constant of 0.05.

## Table S33D - Principal component regression analysis on six predictor variables and protein evolutionary rate for 639 yeast proteins in the “LC-multiple” dataset.

|  | Principal Components | | | | | | |
| --- | --- | --- | --- | --- | --- | --- | --- |
|  | 1 | 2 | 3 | 4 | 5 | 6 | All |
| Percent variance explained in dN | 30.28*** | 2.58** | 1.87* | 1.33* | 0.33 | 0.20 | 36.59*** |
| Percent contributions |  | | | | | | |
| mRNA abundance | **31.9** | 11.0 | 0.7 | **50.5** | 5.6 | 0.2 |  |
| protein abundance | **30.4** | 12.1 | 3.2 | **46.3** | 4.5 | 3.5 |  |
| gene dispensability | 12.3 | 0.8 | 0.1 | 0.5 | **74.9** | 11.3 |  |
| gene pleiotropy | 0.0 | **27.0** | **62.6** | 0.7 | 3.9 | 5.8 |  |
| ePPID | 19.0 | **23.2** | 0.5 | 1.9 | 0.5 | **54.8** |  |
| betweenness | 6.3 | **25.8** | **32.8** | 0.1 | 10.6 | **24.4** |  |

Note: ^#^P<0.01; ^*^P<10^-3^; ^**^P<10^-6^; ^***^P<10^-9^. Bold indicates that the predictor variable contributes at least 20% to the corresponding principal component. ePPID and betweenness are not log transformed.

## Table S34A - Principal component regression analysis on six predictor variables and protein evolutionary rate for 1,502 yeast proteins in the “Updated-HC” dataset.

|  | Principal Components | | | | | | |
| --- | --- | --- | --- | --- | --- | --- | --- |
|  | 1 | 2 | 3 | 4 | 5 | 6 | All |
| Percent variance explained in dN | 20.76*** | 13.06*** | 1.33** | 0.25 | 0.16 | 0.03 | 35.59*** |
| Percent contributions |  | | | | | | |
| mRNA abundance | 11.4 | **38.0** | 0.2 | **44.4** | 0.8 | 5.2 |  |
| protein abundance | 13.9 | **33.7** | 1.1 | **38.2** | 0.3 | 12.7 |  |
| gene dispensability | 15.2 | 1.1 | **32.6** | 0.9 | **46.2** | 3.9 |  |
| gene pleiotropy | 12.0 | 6.5 | **63.0** | 0.3 | 18.1 | 0.0 |  |
| ePPID | **28.8** | 5.8 | 3.0 | 9.3 | 6.2 | **46.7** |  |
| betweenness | 18.6 | 14.9 | 0.1 | 6.8 | **28.3** | **31.3** |  |

Note: ^#^P<0.01; ^*^P<10^-3^; ^**^P<10^-6^; ^***^P<10^-9^. Bold indicates that the predictor variable contributes at least 20% to the corresponding principal component. ePPID and betweenness are added by a smaller constant of 0.2.

## Table S34B - Principal component regression analysis on six predictor variables and protein evolutionary rate for 1,502 yeast proteins in the “Updated-HC” dataset.

|  | Principal Components | | | | | | |
| --- | --- | --- | --- | --- | --- | --- | --- |
|  | 1 | 2 | 3 | 4 | 5 | 6 | All |
| Percent variance explained in dN | 20.73*** | 13.01*** | 1.32** | 0.19 | 0.17 | 0.15 | 35.57*** |
| Percent contributions |  | | | | | | |
| mRNA abundance | 11.6 | **37.8** | 0.2 | 0.8 | **49.6** | 0.0 |  |
| protein abundance | 14.2 | **33.4** | 1.1 | 0.3 | **49.5** | 1.5 |  |
| gene dispensability | 15.0 | 1.0 | **33.9** | **46.1** | 0.0 | 3.9 |  |
| gene pleiotropy | 12.1 | 6.6 | **61.8** | 19.1 | 0.4 | 0.0 |  |
| ePPID | **28.3** | 6.0 | 2.9 | 7.2 | 0.3 | **55.2** |  |
| betweenness | 18.7 | 15.1 | 0.1 | **26.5** | 0.3 | **39.3** |  |

Note: ^#^P<0.01; ^*^P<10^-3^; ^**^P<10^-6^; ^***^P<10^-9^. Bold indicates that the predictor variable contributes at least 20% to the corresponding principal component. ePPID and betweenness are added by a smaller constant of 0.1.

## Table S34C - Principal component regression analysis on six predictor variables and protein evolutionary rate for 1,502 yeast proteins in the “Updated-HC” dataset.

|  | Principal Components | | | | | | |
| --- | --- | --- | --- | --- | --- | --- | --- |
|  | 1 | 2 | 3 | 4 | 5 | 6 | All |
| Percent variance explained in dN | 20.79*** | 12.89*** | 1.31** | 0.22 | 0.21 | 0.15 | 35.57*** |
| Percent contributions |  | | | | | | |
| mRNA abundance | 11.9 | **37.5** | 0.2 | 0.8 | 0.4 | **49.2** |  |
| protein abundance | 14.6 | **33.0** | 1.1 | 0.3 | 0.6 | **50.4** |  |
| gene dispensability | 14.9 | 1.0 | **34.9** | **46.0** | 3.2 | 0.0 |  |
| gene pleiotropy | 12.2 | 6.7 | **60.9** | 19.9 | 0.0 | 0.4 |  |
| ePPID | **27.7** | 6.3 | 2.8 | 8.3 | **54.9** | 0.0 |  |
| betweenness | 18.6 | 15.5 | 0.1 | **24.8** | **41.0** | 0.0 |  |

Note: ^#^P<0.01; ^*^P<10^-3^; ^**^P<10^-6^; ^***^P<10^-9^. Bold indicates that the predictor variable contributes at least 20% to the corresponding principal component. ePPID and betweenness are added by a smaller constant of 0.05.

## Table S34D - Principal component regression analysis on six predictor variables and protein evolutionary rate for 1,502 yeast proteins in the “Updated-HC” dataset.

|  | Principal Components | | | | | | |
| --- | --- | --- | --- | --- | --- | --- | --- |
|  | 1 | 2 | 3 | 4 | 5 | 6 | All |
| Percent variance explained in dN | 27.69*** | 6.91*** | 1.14** | 0.13 | 0.06 | 0.01 | 35.94*** |
| Percent contributions |  | | | | | | |
| mRNA abundance | **21.4** | **25.5** | 0.3 | **48.2** | 3.2 | 1.4 |  |
| protein abundance | **22.9** | **24.1** | 1.2 | **44.7** | 1.0 | 6.1 |  |
| gene dispensability | 15.4 | 3.0 | **43.7** | 1.1 | **23.5** | 13.3 |  |
| gene pleiotropy | 7.1 | 4.2 | **49.6** | 0.7 | **38.2** | 0.2 |  |
| ePPID | **26.3** | 16.6 | 1.3 | 3.7 | 2.1 | **50.1** |  |
| betweenness | 7.0 | **26.6** | 3.8 | 1.7 | **32.0** | **28.9** |  |

Note: ^#^P<0.01; ^*^P<10^-3^; ^**^P<10^-6^; ^***^P<10^-9^. Bold indicates that the predictor variable contributes at least 20% to the corresponding principal component. ePPID and betweenness are not log transformed.

## Table S35A - Principal component regression analysis on six predictor variables and protein evolutionary rate for 913 yeast proteins in the “DIP-CORE” dataset.

|  | Principal Components | | | | | | |
| --- | --- | --- | --- | --- | --- | --- | --- |
|  | 1 | 2 | 3 | 4 | 5 | 6 | All |
| Percent variance explained in dN | 23.77*** | 10.03*** | 1.01* | 0.39 | 0.30 | 0.17 | 35.67*** |
| Percent contributions |  | | | | | | |
| mRNA abundance | 17.2 | **30.2** | **45.4** | 4.0 | 2.0 | 1.3 |  |
| protein abundance | 19.1 | **26.8** | **33.2** | **20.3** | 0.6 | 0.0 |  |
| gene dispensability | 13.8 | 0.6 | 0.4 | 0.3 | **51.0** | **34.0** |  |
| gene pleiotropy | 9.1 | 9.9 | 0.2 | 0.1 | **45.7** | **35.0** |  |
| ePPID | **26.1** | 7.4 | 12.0 | **41.1** | 0.0 | 13.3 |  |
| betweenness | 14.7 | **25.1** | 8.8 | **34.3** | 0.8 | 16.3 |  |

Note: ^#^P<0.01; ^*^P<10^-3^; ^**^P<10^-6^; ^***^P<10^-9^. Bold indicates that the predictor variable contributes at least 20% to the corresponding principal component. ePPID and betweenness are added by a smaller constant of 0.2.

## Table S35B - Principal component regression analysis on six predictor variables and protein evolutionary rate for 913 yeast proteins in the “DIP-CORE” dataset.

|  | Principal Components | | | | | | |
| --- | --- | --- | --- | --- | --- | --- | --- |
|  | 1 | 2 | 3 | 4 | 5 | 6 | All |
| Percent variance explained in dN | 24.10*** | 9.64*** | 0.88* | 0.54^#^ | 0.30 | 0.20 | 35.66*** |
| Percent contributions |  | | | | | | |
| mRNA abundance | 17.8 | **29.7** | **47.8** | 1.5 | 1.9 | 1.3 |  |
| protein abundance | 19.9 | **26.1** | **38.6** | 14.9 | 0.6 | 0.0 |  |
| gene dispensability | 13.8 | 0.7 | 0.4 | 0.3 | **50.3** | **34.4** |  |
| gene pleiotropy | 9.0 | 10.4 | 0.1 | 0.0 | **46.3** | **34.2** |  |
| ePPID | **25.2** | 7.6 | 7.5 | **45.0** | 0.0 | 14.6 |  |
| betweenness | 14.2 | **25.6** | 5.5 | **38.2** | 0.9 | 15.5 |  |

Note: ^#^P<0.01; ^*^P<10^-3^; ^**^P<10^-6^; ^***^P<10^-9^. Bold indicates that the predictor variable contributes at least 20% to the corresponding principal component. ePPID and betweenness are added by a smaller constant of 0.1.

## Table S35C - Principal component regression analysis on six predictor variables and protein evolutionary rate for 913 yeast proteins in the “DIP-CORE” dataset.

|  | Principal Components | | | | | | |
| --- | --- | --- | --- | --- | --- | --- | --- |
|  | 1 | 2 | 3 | 4 | 5 | 6 | All |
| Percent variance explained in dN | 24.43*** | 9.27*** | 0.77^#^ | 0.66^#^ | 0.30 | 0.22 | 35.65*** |
| Percent contributions |  | | | | | | |
| mRNA abundance | 18.3 | **29.1** | **48.9** | 0.5 | 1.9 | 1.2 |  |
| protein abundance | **20.6** | **25.3** | **42.3** | 11.2 | 0.6 | 0.0 |  |
| gene dispensability | 13.9 | 0.8 | 0.4 | 0.4 | **49.8** | **34.6** |  |
| gene pleiotropy | 8.9 | 10.9 | 0.1 | 0.0 | **46.7** | **33.4** |  |
| ePPID | **24.4** | 7.8 | 4.7 | **47.1** | 0.0 | 16.0 |  |
| betweenness | 13.8 | **26.0** | 3.6 | **40.8** | 1.0 | 14.8 |  |

Note: ^#^P<0.01; ^*^P<10^-3^; ^**^P<10^-6^; ^***^P<10^-9^. Bold indicates that the predictor variable contributes at least 20% to the corresponding principal component. ePPID and betweenness are added by a smaller constant of 0.05.

## Table S35D - Principal component regression analysis on six predictor variables and protein evolutionary rate for 913 yeast proteins in the “DIP-CORE” dataset.

|  | Principal Components | | | | | | |
| --- | --- | --- | --- | --- | --- | --- | --- |
|  | 1 | 2 | 3 | 4 | 5 | 6 | All |
| Percent variance explained in dN | 24.23*** | 9.68*** | 0.7^#^ | 0.38 | 0.24 | 0.08 | 35.31*** |
| Percent contributions |  | | | | | | |
| mRNA abundance | **20.9** | **24.5** | **46.2** | 2.1 | 2.6 | 3.6 |  |
| protein abundance | **21.0** | **25.8** | **39.4** | 0.9 | 12.7 | 0.3 |  |
| gene dispensability | 13.4 | 0.2 | 1.4 | **52.7** | 0.6 | **31.7** |  |
| gene pleiotropy | 8.4 | 9.0 | 0.6 | **43.8** | 1.3 | **36.9** |  |
| ePPID | **25.3** | 12.9 | 9.0 | 0.0 | **47.6** | 5.1 |  |
| betweenness | 11.0 | 27.6 | 3.4 | 0.5 | **35.1** | **22.4** |  |

Note: ^#^P<0.01; ^*^P<10^-3^; ^**^P<10^-6^; ^***^P<10^-9^. Bold indicates that the predictor variable contributes at least 20% to the corresponding principal component. ePPID and betweenness are not log transformed.

## Table S36A - Principal component regression analysis on six predictor variables and protein evolutionary rate for 1,698 yeast proteins in the “DIP-FULL” dataset.

|  | Principal Components | | | | | | |
| --- | --- | --- | --- | --- | --- | --- | --- |
|  | 1 | 2 | 3 | 4 | 5 | 6 | All |
| Percent variance explained in dN | 24.86*** | 9.11*** | 1.61*** | 0.19 | 0.13 | 0.03 | 35.93*** |
| Percent contributions |  | | | | | | |
| mRNA abundance | 15.0 | **34.5** | 0.5 | 9.5 | 0.7 | **39.7** |  |
| protein abundance | 16.7 | **30.7** | 1.1 | 3.9 | 0.1 | **47.4** |  |
| gene dispensability | 10.8 | 0.6 | **28.4** | 2.0 | **58.0** | 0.1 |  |
| gene pleiotropy | 10.5 | 9.2 | **64.3** | 0.0 | 15.6 | 0.3 |  |
| ePPID | **27.1** | 7.7 | 4.8 | **46.3** | 7.2 | 6.9 |  |
| betweenness | 19.9 | 17.2 | 0.9 | **38.2** | 18.3 | 5.6 |  |

Note: ^#^P<0.01; ^*^P<10^-3^; ^**^P<10^-6^; ^***^P<10^-9^. Bold indicates that the predictor variable contributes at least 20% to the corresponding principal component. ePPID and betweenness are added by a smaller constant of 0.2.

## Table S36B - Principal component regression analysis on six predictor variables and protein evolutionary rate for 1,698 yeast proteins in the “DIP-FULL” dataset.

|  | Principal Components | | | | | | |
| --- | --- | --- | --- | --- | --- | --- | --- |
|  | 1 | 2 | 3 | 4 | 5 | 6 | All |
| Percent variance explained in dN | 24.82*** | 9.09*** | 1.61*** | 0.24 | 0.14 | 0.01 | 35.91*** |
| Percent contributions |  | | | | | | |
| mRNA abundance | 15.3 | **34.2** | 0.5 | 15.4 | 0.8 | **33.9** |  |
| protein abundance | 17.1 | **30.4** | 1.1 | 8.7 | 0.2 | **42.6** |  |
| gene dispensability | 10.8 | 0.6 | **29.0** | 1.7 | **57.7** | 0.2 |  |
| gene pleiotropy | 10.7 | 9.2 | **63.6** | 0.0 | 16.1 | 0.4 |  |
| ePPID | **26.4** | 8.2 | 4.9 | **40.2** | 7.7 | 12.5 |  |
| betweenness | 19.7 | 17.5 | 0.8 | **34.1** | 17.6 | 10.4 |  |

Note: ^#^P<0.01; ^*^P<10^-3^; ^**^P<10^-6^; ^***^P<10^-9^. Bold indicates that the predictor variable contributes at least 20% to the corresponding principal component. ePPID and betweenness are added by a smaller constant of 0.1.

## Table S36C - Principal component regression analysis on six predictor variables and protein evolutionary rate for 1,698 yeast proteins in the “DIP-FULL” dataset.

|  | Principal Components | | | | | | |
| --- | --- | --- | --- | --- | --- | --- | --- |
|  | 1 | 2 | 3 | 4 | 5 | 6 | All |
| Percent variance explained in dN | 24.85*** | 9.02*** | 1.61*** | 0.27^#^ | 0.15 | 0.00 | 35.9*** |
| Percent contributions |  | | | | | | |
| mRNA abundance | 15.6 | **33.8** | 0.5 | **26.6** | 0.8 | **22.6** |  |
| protein abundance | 17.6 | **30.0** | 1.0 | 19.6 | 0.2 | **31.6** |  |
| gene dispensability | 10.8 | 0.5 | **29.6** | 1.2 | **57.3** | 0.5 |  |
| gene pleiotropy | 10.8 | 9.3 | **62.9** | 0.0 | 16.5 | 0.5 |  |
| ePPID | **25.7** | 8.6 | 5.1 | **28.0** | 8.3 | **24.1** |  |
| betweenness | 19.4 | 17.8 | 0.7 | **24.6** | 16.9 | **20.6** |  |

Note: ^#^P<0.01; ^*^P<10^-3^; ^**^P<10^-6^; ^***^P<10^-9^. Bold indicates that the predictor variable contributes at least 20% to the corresponding principal component. ePPID and betweenness are added by a smaller constant of 0.05.

## Table S36D - Principal component regression analysis on six predictor variables and protein evolutionary rate for 1,698 yeast proteins in the “DIP-FULL” dataset.

|  | Principal Components | | | | | | |
| --- | --- | --- | --- | --- | --- | --- | --- |
|  | 1 | 2 | 3 | 4 | 5 | 6 | All |
| Percent variance explained in dN | 28.22*** | 6.48*** | 1.20** | 0.10 | 0.03 | 0.00 | 36.03*** |
| Percent contributions |  | | | | | | |
| mRNA abundance | **22.3** | **22.7** | 0.5 | **48.3** | 4.6 | 1.6 |  |
| protein abundance | **23.1** | **22.6** | 0.8 | **42.3** | 2.0 | 9.1 |  |
| gene dispensability | 11.9 | 0.3 | **51.2** | 0.8 | **31.4** | 4.3 |  |
| gene pleiotropy | 7.1 | 2.0 | **45.9** | 0.3 | **44.5** | 0.1 |  |
| ePPID | **25.4** | 16.8 | 0.7 | 5.3 | 2.6 | **49.3** |  |
| betweenness | 10.1 | **35.6** | 0.9 | 2.9 | 14.9 | **35.6** |  |

Note: ^#^P<0.01; ^*^P<10^-3^; ^**^P<10^-6^; ^***^P<10^-9^. Bold indicates that the predictor variable contributes at least 20% to the corresponding principal component. ePPID and betweenness are not log transformed.

## Table S37 - The effect of co-expression thresholds on Spearman correlation between ePPID and protein evolutionary rate.

| **Protein interaction datasets** | *n* | PPID vs. dN | ePPID vs. dN (FDR=0.002 PER=20%) | ePPID vs. dN (FDR=0.001 PER=10%) | ePPID vs. dN (FDR=0.0005 PER=5%) |
| --- | --- | --- | --- | --- | --- |
|  |  | *rho*(*p*) | *rho*(*p*) | *rho*(*p*) | *rho*(*p*) |
| Y2H-union | 1,104 | -0.0487(1.06e-01) | **-0.109(2.69e-04)** | **-0.142(2.25e-06)** | **-0.138(3.92e-06)** |
| Combined-AP/MS | 922 | **-0.158(1.46e-06)** | **-0.233(8.72e-13)** | **-0.251(1.03e-14)** | **-0.268(1.30e-16)** |
| LC-multiple | 894 | **-0.172(2.46e-07)** | **-0.240(3.19e-13)** | **-0.267(4.72e-16)** | **-0.275(5.98e-17)** |
| Updated-HC | 2,245 | **-0.183(2.62e-18)** | **-0.232(6.71e-29)** | **-0.242(2.58e-31)** | **-0.245(3.88e-32)** |
| DIP-CORE | 1,342 | **-0.152(2.33e-08)** | **-0.234(3.82e-18)** | **-0.254(3.69e-21)** | **-0.267(2.79e-23)** |
| DIP-FULL | 2,572 | **-0.233(4.56e-33)** | **-0.267(3.64e-43)** | **-0.271(1.40e-44)** | **-0.282(2.71e-48)** |
| FYI | 779 | **-0.239(1.47e-11)** | **-0.285(4.60e-16)** | **-0.314(2.44e-19)** | **-0.328(5.51e-21)** |
| SIN | 436 | **-0.234(8.10e-07)** | **-0.324(4.41e-12)** | **-0.339(3.30e-13)** | **-0.339(3.64e-13)** |
| Eight-union | 2,695 | **-0.258(3.86e-42)** | **-0.290(3.39e-53)** | **-0.294(5.52e-55)** | **-0.300(4.77e-57)** |

dN represents protein evolutionary rate measured by non-synonymous substitutions. *n* is the number of proteins for which both PPID and protein evolutionary rate are available. *rho* is Spearman rank correlation coefficient, and *p* is the corresponding statistical significance. Bold indicates that *p* is significant at the statistical significance level of 0.05.

## Table S38 - The effect of co-expression thresholds on percent variance of protein evolutionary rate explained by ePPID across the nine protein interaction datasets.

| **Protein interaction datasets** | *n* | **Percent variance explained in dN** | | | |
| --- | --- | --- | --- | --- | --- |
|  |  | PPID | ePPID (FDR=0.002 PER=20%) | ePPID (FDR=0.001 PER=10%) | ePPID (FDR=0.0005 PER=5%) |
| Y2H-union | 1,104 | 0.278 | 0.76 | 1.14 | 1.09 |
| Combined-AP/MS | 922 | 2.80 | 4.53 | 4.63 | 4.86 |
| LC-multiple | 894 | 1.63 | 3.82 | 4.40 | 4.60 |
| Updated-HC | 2,245 | 1.13 | 3.99 | 4.36 | 4.43 |
| DIP-CORE | 1,342 | 1.18 | 2.10 | 2.62 | 2.75 |
| DIP-FULL | 2,572 | 2.37 | 4.23 | 4.44 | 4.63 |
| FYI | 779 | 4.00 | 5.91 | 6.44 | 6.67 |
| SIN | 436 | 5.25 | 7.98 | 8.67 | 8.53 |
| Eight-union | 2,695 | 4.21 | 5.37 | 5.22 | 5.24 |

dN represents protein evolutionary rate measured by non-synonymous substitutions. *n* is the number of proteins for which both PPID and protein evolutionary rate are available.
